# Construction of small RNA-mediated gene regulatory networks in the roots of rice (*Oryza sativa*)

**DOI:** 10.1186/1471-2164-14-510

**Published:** 2013-07-27

**Authors:** Xiaoxia Ma, Chaogang Shao, Huizhong Wang, Yongfeng Jin, Yijun Meng

**Affiliations:** 1 College of Life and Environmental Sciences, Hangzhou Normal University, Xuelin Street 16#, Xiasha, Hangzhou 310036, P. R. China; 2 College of Life Sciences, Huzhou Teachers College, Xueshi Road 1#, Huzhou 313000, P. R. China; 3 Institute of Biochemistry, College of Life Sciences, Zhejiang University, Hangzhou 310058, P. R. China

**Keywords:** Small RNA, Whole root, Root tip, Argonaute 1, Degradome, Network, Rice (*Oryza sativa*)

## Abstract

**Background:**

The root systems play essential roles for plants to anchorage to the soil, and to exploit the mineral and water resources. The molecular mechanisms underlying root development have been extensively studied to improve root system architecture, especially for the crops. Several microRNA (miRNA) families have been demonstrated to be involved in plant root development. However, whether the other small RNA (sRNA) species, which occupy a dominant portion of the plant endogenous sRNA population, possess potential roles in root development remains unclear.

**Results:**

In this study, by using sRNA high-throughput sequencing data, we made a comparison of the sRNA accumulation levels between the rice root tips and the whole roots. The sRNAs highly accumulated in the root tips and in the whole roots were extracted respectively. After Argonaute 1 (AGO1) enrichment analysis, the sRNAs with great potential of performing target cleavages were included for target prediction and degradome sequencing data-based validation. As a result, lists of the targets regulated by the AGO1-enriched sRNAs were obtained for both the root tips and the whole roots. Further evidences were identified from microarray data of the target genes to support some of the sRNA—target interactions. Specifically, the expression patterns of certain target genes in the root tips and the whole roots were contrary to those of the regulating sRNAs. Besides, several targets were indicated to play important roles in root development based on literature mining.

**Conclusions:**

Taken together, the regulatory networks mediated by the sRNAs highly accumulated in the root tips or in the whole roots could advance our current understanding of the sRNA-involved molecular mechanisms underlying rice root development. And, the sRNA—target lists could serve as the basis for further functional investigations.

## Background

As one of the important organs, the root system helps a plant to anchorage to the soil, and to absorb mineral nutrients and water for normal development and growth during its life cycle. Since the root system architecture greatly affects the efficiency of nutrient and water absorption, the molecular factors influencing root development have been extensively investigated in order to facilitate genetic modification of the agronomic traits of the plants, especially for the crops [1,2].

MicroRNAs (miRNAs), a small RNA (sRNA) species, were identified to be involved in various biological processes of plants during the past ten years [3,4]. Certain miRNA families are indispensable for the establishment of the root system architecture [5], and are implicated in the transduction of multiple signals, such as nutrition, hormone and stress [6]. For examples, miR160 is required for root cap formation by targeting *Auxin Response Factor* (*ARF*) *10* and *16* in *Arabidopsis*[7]. miR164-mediated regulation of *NAM*/*ATAF*/*CUC 1* (*NAC1*) is involved in *Arabidopsis* lateral root development through downregulating auxin signals [8]. Both miR160 and miR167 play important roles in adventitious rooting in *Arabidopsis*[9].

Based on high-throughput sequencing (HTS), a much more huge sRNA population was uncovered in plants in addition to the miRNAs. Whether some of these sRNAs play potential roles in root development remains unclear, although a few experimental hints point to this possibility recently [10]. To partially address this issue, we took advantage of the public sRNA HTS data originated from the root tips and the whole roots of rice (*Oryza sativa*). We made a comparison of the sRNA accumulation levels between the two biological samples to identify the sRNAs highly accumulated in the root tips and in the whole roots respectively. Then, these sRNAs were subjected to Argonaute 1 (AGO1) enrichment analysis. And, those enriched in AGO1 were considered to have potential of performing target cleavages, and were included in target prediction and degradome sequencing data-based validation. Based on the target lists, regulatory networks mediated by the AGO1-enriched sRNAs were established for both the root tips and the whole roots. Notably, within the networks, the expression patterns of several target genes, based on the microarray data, were contrary to those of the regulating sRNAs, supporting the interactions between the sRNAs and the targets. Besides, literature mining indicated that certain target genes played potential roles in root development. These evidences indicate the reliability of the networks. Our results could advance the current understanding of the regulatory roles of the sRNAs, in addition to the miRNAs, in plant root growth and development.

## Results and discussion

### Identification of the sRNAs highly accumulated in the root tips or in the whole roots of rice

**Figure 1:**
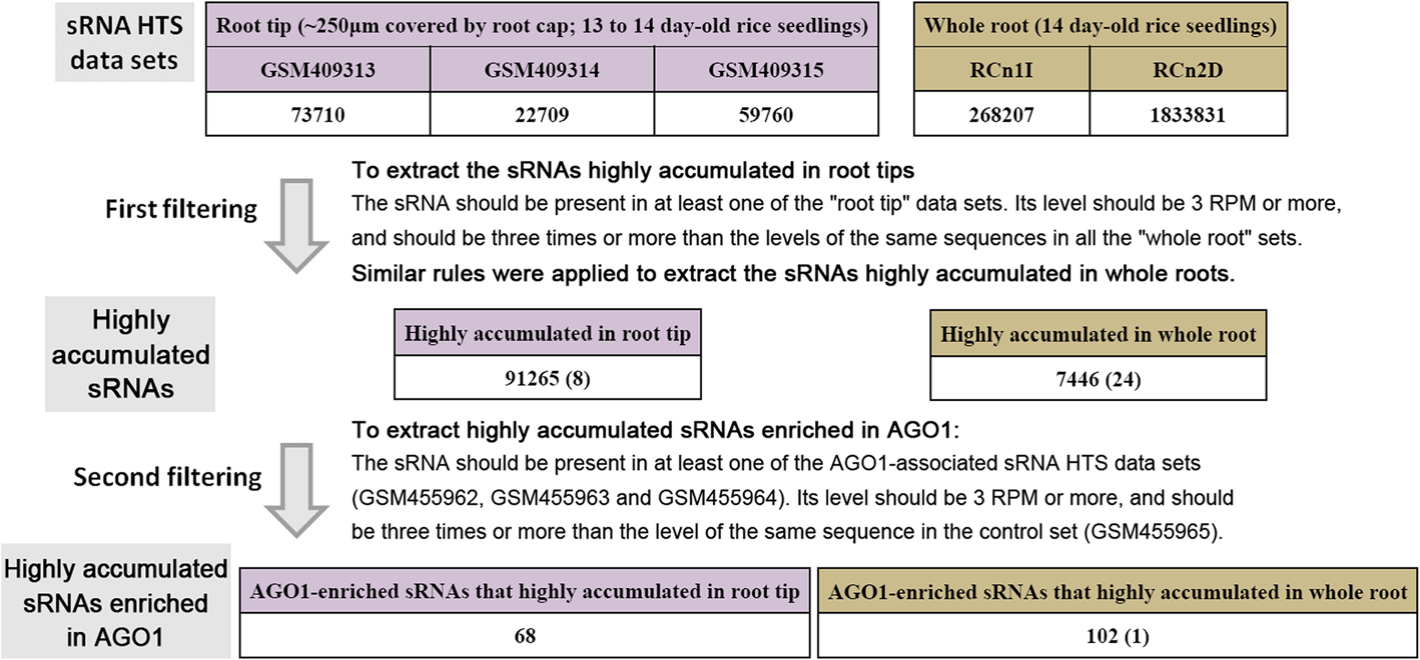
**Workflow for the identification of the rice small RNAs (sRNAs) highly accumulated in the root tips and the whole roots based on high-throughput sequencing data.** The total numbers of the identified sRNAs including microRNAs are shown in the corresponding boxes. The numbers in the brackets indicate the numbers of the microRNA sequences identified in this study. The rules for the two-step filtering are also shown.

We took advantage of the public sRNA HTS data of rice. The data sets could be divided into two groups: the ones (GSM409313, GSM409314 and GSM409315) prepared from the rice root tips (~250 μm) and the ones (RCn1I and RCn2D) from the whole roots (Figure 1). According to the previous reports [11,12], all of these data sets originated from ~14-day-old rice (*Oryza sativa* L. ssp. *japonica*) seedlings, thus making the two data groups more comparable. To extract the sRNAs highly accumulated in the root tips, the following rules were applied: (1) the sRNA should be present in at least one of the “root tip” data sets (GSM409313, GSM409314 and GSM409315); (2) its normalized accumulation levels should be 3 RPM (reads per million; the normalization step is similar to that for the degradome sequencing data; see Methods for detail) or higher, and should be three times or more than the levels of the same sequences in all the “whole root” data sets (RCn1I and RCn2D). Similar rules were applied to identify the sRNAs highly accumulated in the whole roots. As a result, 91,265 sRNAs including eight miRNA sequences (osa-miR169i-5p.2, osa-miR390-3p, osa-miR531b, osa-miR1430, osa-miR1847.2, osa-miR1865-5p, osa-miR1874-3p and osa-miR5082) showed high abundances in the root tips (Additional file 1), and 7,446 sRNAs including 24 miRNA sequences (osa-miR156l-5p, osa-miR164d, osa-miR164e, osa-miR166k-3p/osa-miR166l-3p, osa-miR169f.2, osa-miR171f-5p, osa-miR393a, osa-miR408-5p, osa-miR435, osa-miR528-5p, osa-miR529b, osa-miR812i/osa-miR812j/osa-miR812g/osa-miR812h, osa-miR812r, osa-miR812s, osa-miR1318-3p, osa-miR1320-5p, osa-miR1425-3p, osa-miR1427, osa-miR1432, osa-miR1861h/osa-miR1861j, osa-miR3979-3p, osa-miR5788, osa-miR5795 and osa-miR5802) were highly accumulated in the whole roots (Additional file 2). Since the “whole root” includes the “root tip” cells, we reasoned that the high levels of the 7,446 sRNAs in the whole roots were largely attributed to their high abundances within the elongation and the maturation zones of the rice roots including the main roots, the adventitious roots and the lateral roots.

In plants, some of the sRNAs, such as the miRNAs, exert their regulatory activities through target cleavages [3,4,13]. The sRNAs incorporate into specific AGO-associated RNA-induced silencing complexes (RISCs), and guide the complexes to the target transcripts to perform cleavages. It has been reported that AGO1 is an RNA slicer selectively recruiting miRNAs and short interfering RNAs in plants [14,15]. In this consideration, we set out to identify AGO1-enriched sRNAs from the sRNAs highly accumulated in the root tips and the whole roots obtained above. The following rules were applied: (1) the sRNAs should be detectable in at least one of the AGO1-associated sRNA HTS data sets (GSM455962, GSM455963 and GSM455964); (2) its normalized accumulation levels should be 3 RPM or higher, and should be three times or more than the levels of the same sequence in the control set (GSM455965). As a result, 68 AGO1-enriched sRNAs were extracted from the sRNAs highly accumulated in the root tips, and 102 AGO1-enriched sRNAs including osa-miR528-5p were identified from the sRNAs highly accumulated in the whole roots (Figure 1, Additional file 3 and Additional file 4).

**Figure 2:**
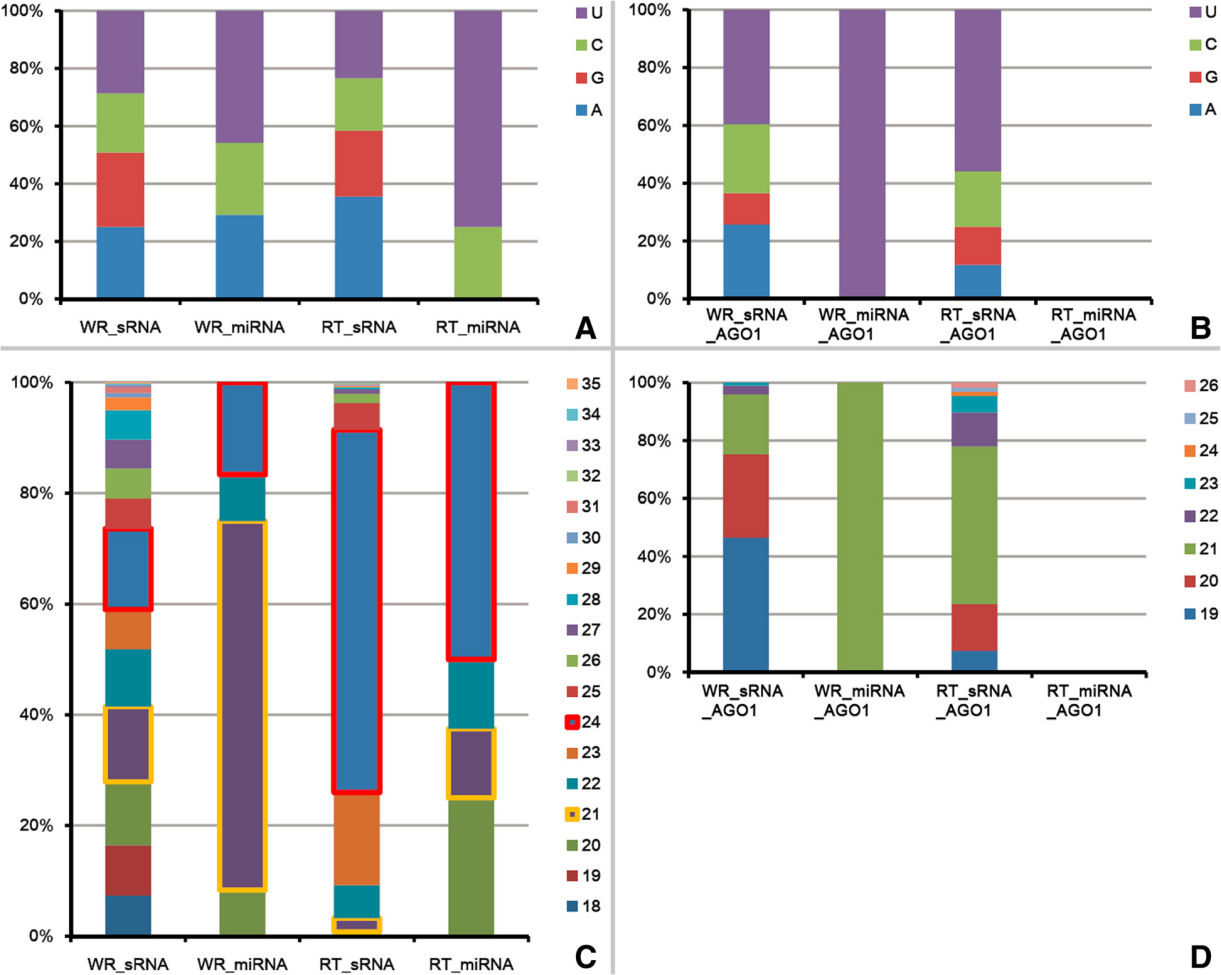
**Sequence characteristics of the small RNAs (sRNAs) and the microRNAs (miRNAs) highly accumulated in the root tips and the whole roots of rice. (A)** 5^′^ terminal compositions (measured by percentages) of the sRNAs and the miRNAs highly accumulated in the root tips (RT_sRNA and RT_miRNA) and the whole roots (WR_sRNA and WR_miRNA). **(B)**v5^′^ terminal compositions (measured by percentages) of the Argonaute 1 (AGO1)-enriched sRNAs and miRNAs highly accumulated in the root tips (RT_sRNA_AGO1 and RT_miRNA_AGO1) and the whole roots (WR_sRNA_AGO1 and WR_miRNA_AGO1). **(C)** Sequence length distributions (measured by percentages) of the sRNAs and the miRNAs highly accumulated in the root tips and the whole roots. The occupancies of the sRNAs and the miRNAs of 21 nt and 24 nt are highlighted by yellow and red boxes respectively. **(D)** Sequence length distributions (measured by percentages) of the AGO1-enriched sRNAs and miRNAs highly accumulated in the root tips and the whole roots.

Next, the sequence characteristics of the sRNAs before and after the AGO1 enrichment filtering were examined separately. For both the sRNAs highly accumulated in the root tips and those highly abundant in the whole roots, no significant bias of the 5′ terminal nucleotide compositions was observed before the AGO1 enrichment filtering (Figure 2A). However, for those enriched in AGO1 complexes, the proportion of 5′ U (uridine)-started sRNAs was increased. Specifically, for the sRNAs highly accumulated in the whole roots, the percentage of 5′ U-started sRNAs increased from 28.68% to 39.60%. For those highly accumulated in the root tips, the percentage increased from 23.36% to 55.88% (Figure 2B). Different from the miRNAs, 21 nt (nucleotide) is not the dominant length for the other sRNAs highly accumulated in the whole roots. More interestingly, 24 nt is the dominant length for both the sRNAs and the miRNAs highly accumulated in the root tips (Figure 2C). After the AGO1 enrichment filtering, the sRNAs ranging from 19 nt to 21 nt occupied a dominant portion (96.04%) of the sRNAs abundant in the whole roots. And, for the sRNAs highly accumulated in the root tips, the 21 nt ones increased significantly (from 2.49% to 54.41%) (Figure 2D). Considering the previous report on the sequence characteristics of AGO1-associated sRNAs [16], our AGO1 enrichment filtering tended to selectively extract 5′ U-started, 19 to 21 nt sRNAs from the sRNAs highly accumulated in the root tips and the whole roots, respectively.

### Identification of the targets of the AGO1-enriched sRNAs and network construction

As mentioned above, the sRNAs associated with the AGO1 silencing complexes are more likely to modulate gene expression through target cleavages. In this analysis, only the AGO1-enriched sRNAs were subjected to target prediction. miRU algorithm [17,18] with default parameters was employed for this prediction. As a result, 1,699 target binding sites were predicted for the 68 AGO1-enriched sRNAs highly expressed in the root tips, and 1,979 binding sites were predicted for the 102 AGO1-enriched sRNAs (including osa-miR528-5p) highly expressed in the whole roots.

**Figure 3:**
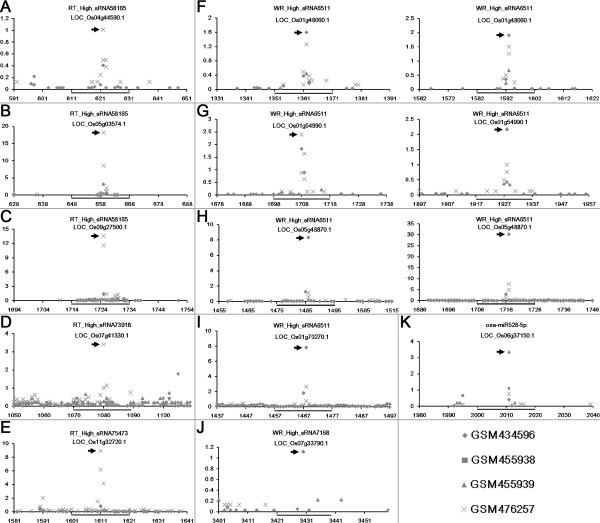
**Degradome sequencing data-based validation of the targets regulated by the small RNAs (sRNAs) highly accumulated in the root tips and the whole roots of rice.** For all the figure panels **(A** to **K)**, the IDs of the target transcripts and the regulating sRNAs are listed on the top. The *y* axes measure the intensity (in RPM, reads per million) of the degradome signals, and the *x* axes indicate the positions of the cleavage signals on the target transcripts. The binding sites of the sRNA regulators on their target transcripts were denoted by gray horizontal lines, and the dominant cleavage signals were marked by gray arrowheads. The figure keys for the degradome signatures from four libraries are shown on the bottom right. Please note, for **(F)** to **(H)**, each target transcript possesses two binding sites of WR_High_sRNA6511.

Degradome sequencing data is a valuable resource for large-scale validation of the sRNA—target interactions [19,20]. Four degradome sequencing data sets of rice (see detail in Methods) were recruited to do a comprehensive validation of the predicted targets according to the analytical workflow proposed previously [21]. Considering the reports that some evident cleavage signals out of the canonical regions (10^th^ to 11^th^ nt of the regulating sRNAs) were observed [22-25], the binding sites with prominent cleavage signals resided within 8^th^ to 12^th^ nt of the regulating sRNAs were retained. After target plot-based screening, 22 interactions involving 16 target genes and six sRNAs highly accumulated in the root tips, and 171 interactions involving 29 genes and 27 sRNAs (including osa-miR528-5p) highly abundant in the whole roots passed the validation (Additional file 5: Figure S1 and Additional file 6: Figure S2). Among the validated sRNA—target pairs, we observed that many binding sites were supported by evident degradome signatures resided within the canonical cleavage sites (10^th^ to 11^th^ nt of the regulating sRNAs) (Figure 3). In addition to osa-miR528-5p (Figure 3K), several sRNAs, such as RT_High_sRNA58165, RT_High_sRNA73918, RT_High_RNA75473, WR_High_sRNA6511 and WR_High_sRNA7168, cleaved the corresponding transcripts at the canonical sites. For WR_High_sRNA6511, we observed that the sRNA possessed two binding sites on the transcripts LOC_Os01g48060.1, LOC_Os01g54990.1 and LOC_Os05g48870.1, separately (Figure 3 F, 3G and 3H). Notably, all of these sRNAs are distinct from the current plant miRNA registries in miRBase (release 19) [26]. The result indicates that the sRNAs, which remain to be classified, could guide AGO1 complexes to perform miRNA-like target cleavages in the root systems of rice.

Based on the target lists obtained above, the sRNA-mediated regulatory networks were constructed in the root tips and the whole roots by using Cytoscape [27] (Additional file 7: Figure S3 and Additional file 8: Figure S4). At first glance, RT_High_sRNA58165 regulates 16 transcripts encoded by ten different genes (Additional file 7: Figure S3), pointing to the possibility that this sRNA might play an essential role in root tip formation. More complex network was observed for those sRNAs highly accumulated in the whole roots. In some cases, one target is shared by two or more sRNAs. For example, LOC_Os07g29820.1 is regulated by WR_High_sRNA7168 and WR_High_sRNA6608. LOC_Os09g23730.1 is regulated by WR_High_sRNA317, WR_High_sRNA1124, WR_High_sRNA1878, WR_High_sRNA2359 and WR_High_sRNA2909 (Additional file 8: Figure S4).

### Biological meanings of the networks supported by target expression- and literature mining-based evidences

Considering the repressive role of the AGO1-enriched sRNAs in gene expression, we deduced that for the sRNA—target pairs validated above, the target genes should display expression patterns contrary to those of the sRNA regulators. For example, the target gene of a sRNA highly accumulated in the root tips was supposed to have higher expression level in the whole roots than in the root tips. To support this notion, 24 microarray data sets contributed by a previous report [28] were utilized. These data sets enabled us to investigate the expression levels of all the target genes in different parts of the roots (eight different regions: root cap, division zone, elongation zone, maturation zone I, maturation zone II, maturation zone III, maturation zone IV and maturation zone V; each with three replicates). According to the report by Takehisa *et al.*[28], in this analysis, the microarray data sets of the root cap and the division zone were grouped into the “root tip” category (~200 μm), which was anatomically comparable to the sRNA HTS library group (GSM409313, GSM409314 and GSM409315) prepared from the root tips (~250 μm). All the 24 microarray data sets were treated as the “whole root” category in order to make it anatomically consistent with the sRNA HTS library group (RCn1I and RCn2D) prepared from the whole roots. All the microarray data sets were generated from the 10-day-old rice seedlings, which was largely consistent with the 14-day-old seedlings for sRNA library preparation. For the expression level analysis, the mean value of the three replicates was calculated for each target gene.

**Figure 4:**
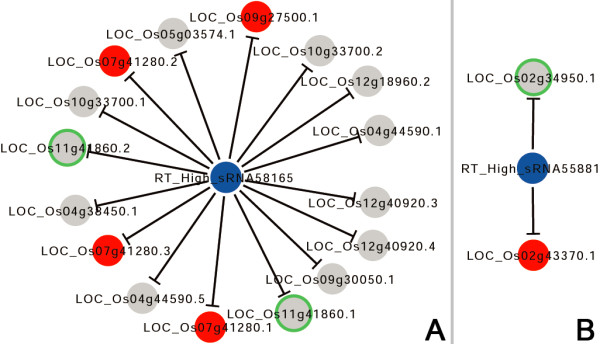
**Certain subnetworks mediated by the small RNAs (sRNAs) highly accumulated in the root tips of rice.** For both **(A)** and **(B)**, the sRNAs are represented by blue nodes, and the targets by gray nodes. Notably, the target nodes in red color are the ones with expression patterns contrary to those of the regulating sRNAs, which are supported by microarray data. The target nodes with green outlines are the ones with potential biological functions in root development based on the literatures. The subnetworks were constructed by using Cytoscape [27].

**Figure 5:**
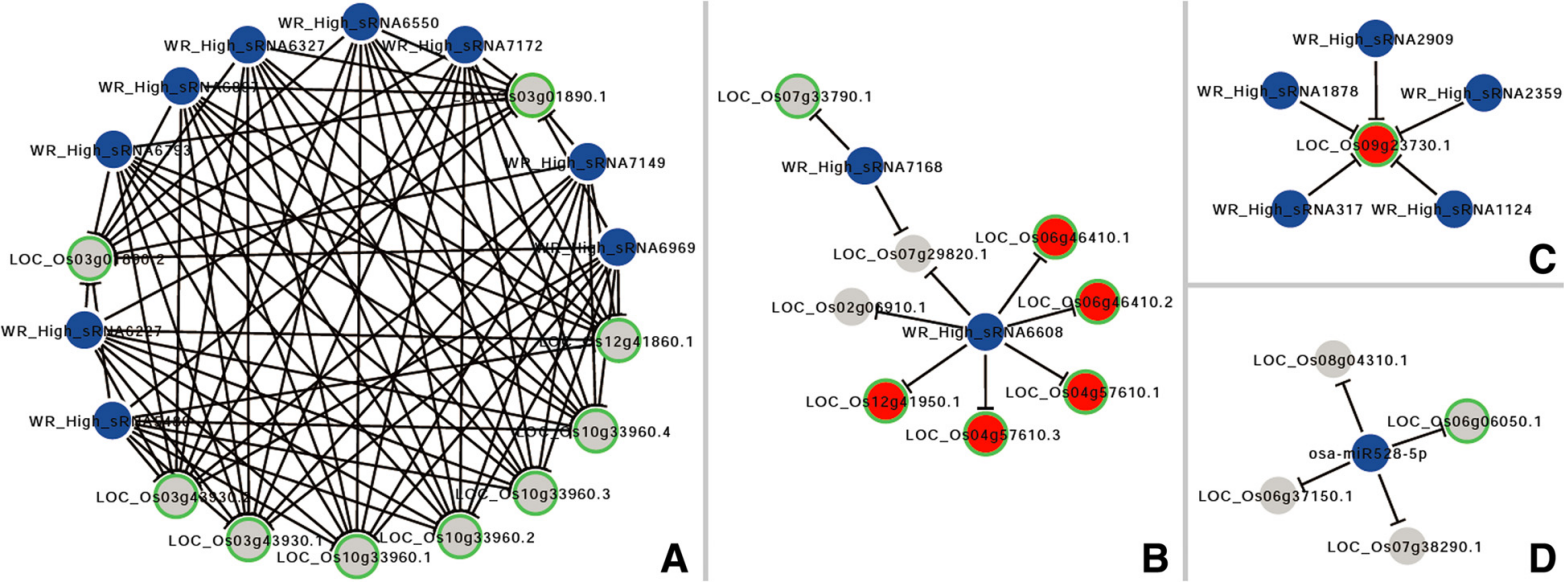
**Certain subnetworks mediated by the small RNAs (sRNAs) highly accumulated in the whole roots of rice.** For all the figure panels **(A)** to **(D)**, the sRNAs are represented by blue nodes, and the targets by gray nodes. Notably, the target nodes in red color are the ones with expression patterns contrary to those of the regulating sRNAs, which are supported by microarray data. The target nodes with green outlines are the ones with potential biological functions in root development based on the literatures. The subnetworks were constructed by using Cytoscape [27].

The microarray data-based analysis provided supportive evidences for some of the sRNA—target interactions. Specifically, the level of LOC_Os02g43370.1 regulated by RT_High_sRNA55881 in the whole roots is 4.53 times higher than its level in the root tips (0.125 vs. 0.028) (Figure 4B and Additional file 9: Table S1). For LOC_Os07g41280.1/LOC_Os07g41280.2/LOC_Os07g41280.3 cleaved by RT_High_sRNA58165, their expression value in the whole roots is 5.547 versus 0.670 in the root tips. Also regulated by RT_High_sRNA58165, the level of LOC_Os09g27500.1 in the whole roots is 0.317 versus 0.023 in the root tips (Figure 4A and Additional file 9: Table S1). For LOC_Os04g57610.1/LOC_Os04g57610.3 regulated by WR_High_sRNA6608, their expression value in the whole roots is 1.355, which is lower than that (3.909) in the root tips. For LOC_Os06g46410.1/LOC_Os06g46410.2 also regulated by WR_High_sRNA6608, their expression value in the whole roots is 0.643 versus 1.463 in the root tips. Also for WR_High_sRNA6608, the level of its target transcript LOC_Os12g41950.1 in the whole roots is 1.736, which is lower than that (4.185) in the root tips (Figure 5B and Additional file 9: Table S1). For LOC_Os09g23730.1 regulated by WR_High_sRNA317, WR_High_sRNA1124, WR_High_sRNA1878, WR_High_sRNA2359 and WR_High_sRNA2909, its expression levels in the whole roots and the root tips are 5.430 and 8.273 respectively (Figure 5C and Additional file 9: Table S1). Taken together, the results demonstrated that at least some of the target genes displayed expression patterns contrary to those of the regulating sRNAs. Failure to validate the regulatory relationships of the remaining sRNA—target pairs by microarray data analysis might attribute to several embedded factors. The most likely reason might be that the expression of the target genes was not only modulated by the identified regulatory sRNAs, but also regulated by the other factors such as transcription factors and epigenetic factors which might disturb the contrary accumulation patterns between the sRNAs and the targets in the root systems. From this point of view, we should recognize that the regulation of the expression of a gene is quite complex, and could not be determined by only one regulator in most cases. Another potential reason is that the target gene might be specifically expressed in the root part in which the regulating sRNAs was highly abundant. For example, the sRNA was highly abundant in the root tips, and the target gene was expressed in the root tips but not in the remaining parts of the rice roots. It will lead to the failure to observe the contrary accumulation pattern between this sRNA—target pair.

We also intended to find some root-related evidences for the target genes based on functional annotations and literature mining. Fortunately, some positive hints were obtained. For examples, based on the annotation from TIGR rice (the rice genome annotation project established by the institute for genome research) [29], LOC_Os02g34950.1 regulated by RT_High_sRNA55881 (Figure 4B) is involved in post-embryonic development. And, its homologous gene in *Arabidopsis*, *AT5G22370*, participates in the organization of microtubules during cell division according to the TAIR (The *Arabidopsis* Information Resource) annotation [30]. Since the root tip includes a meristematic region in an active state of cell division, whether *LOC_Os02g34950* plays an essential role in the formation and/or the maintenance of the root meristem remains to be investigated. Also in the root tips, *LOC_Os11g41860* encoding an F-box domain-containing protein is regulated by RT_High_sRNA58165 based on the degradome sequencing data (Figure 4A). According to a recent report, an F-box gene *LOC_Os02g44990* is involved in promoting root growth in rice [31], pointing to the possibility that *LOC_Os11g41860* is functionally linked to root development. More supportive evidences were found for the target genes within the network mediated by the sRNAs abundant in the whole roots. *LOC_Os03g01890*, *LOC_Os03g43930*, *LOC_Os10g33960* and *LOC_Os12g41860*, all of which encode START domain-containing proteins (homologous to Class III HD-ZIP proteins of *Arabidopsis*), were regulated by the same sRNA regulators, i.e. WR_High_sRNA5480, WR_High_sRNA6227, WR_High_sRNA6327, WR_High_sRNA6550, WR_High_sRNA6793, WR_High_sRNA6897, WR_High_sRNA6969, WR_High_sRNA7149 and WR_High_sRNA7172 (Figure 5A). In *Arabidopsis* and *Medicago truncatula*, miR166-mediated regulation of Class III *HD-ZIP* genes is important for root development [32-34]. More interestingly, although the above nine sRNAs are not miRNAs, WR_High_sRNA6793 shares high sequence similarity with osa-miR166g-3p and osa-miR166h-3p of rice, and the other eight sRNAs share high sequence similarity with miR166 of the other plant species. In this regard, the four target genes regulated by the sRNAs homologous to miR166 might have a conserved role in rice root development. Another class of target genes including *LOC_Os04g57610*, *LOC_Os06g46410* and *LOC_Os12g41950* encode the rice ARF proteins (ARF12, ARF17 and ARF25 respectively) (Figure 5B). The three *ARF* genes share high sequence identity with each other, and *LOC_Os04g57610* was reported to regulate root elongation in rice [35]. Moreover, the homologous *ARF* genes in *Arabidopsis* are regulated by miR167, and are essential for root system development [9,36,37]. Notably, the three *ARF* genes of rice were targeted by WR_High_sRNA6608 sharing high sequence similarity with osa-miR167. *LOC_Os06g06050* targeted by osa-miR528-5p encodes OsFBL27, an F-box domain- and LRR-containing protein (Figure 5D). Its homologous gene *AT2G42620* (*MAX2*, *More Axillary Growth 2*) is implicated in root development in *Arabidopsis*[38-40]. And, miR528 was previously indicated to play a role in auxin signal-mediated rice root development [41]. *LOC_Os09g23730* encoding an HMG-Y-related protein was targeted by five sRNAs highly accumulated in the whole roots (Figure 5C). Based on an early report, ectopic expression of the maize HMGB1 protein causes defects in root development of tobacco seedlings [42]. More direct evidence was found for *LOC_Os07g33790* targeted by WR_High_sRNA7168 (Figure 5B) sharing sequence similarity with miR167 of the other plant species. This target gene encodes GLR (glutamate receptor) 3.4 based on the TIGR annotation. It was reported that GLR3.1 of rice was essential for the maintenance of the root apical meristem [43], and GLR3.2 and GLR3.4 of *Arabidopsis* were involved in lateral root initiation [44]. All these literature-based evidences support the potential roles of the target genes in rice root development.

## Conclusions

Summarily, this study on the sRNAs in the rice root systems could advance our knowledge of the regulatory activities and the biological functions of the plant sRNAs other than the miRNAs. The networks established in this study were supported by the degradome sequencing data, the microarray data and the literature-based evidences, indicating that the networks could provide a basis for in-depth analysis of sRNA-mediated gene regulation in the rice roots.

## Methods

### Data sources

High-throughput sequencing data sets of rice sRNAs were retrieved from GEO (Gene Expression Omnibus; http://www.ncbi.nlm.nih.gov/geo/) [45] and the Rice Small RNA Database 2 belonging to the Next-Gen Sequence Databases (http://mpss.udel.edu/rice_sRNA2/index.php?menu=../common/web/library_info.php?SITE=rice_sRNA2&showAll=true) [46]. Specifically, GSM409313, GSM409314 and GSM409315 downloaded from GEO were prepared from the root tips (~250 μm) of 13 to 14-day old rice seedlings [12]. RCn1I and RCn2D downloaded from the Next-Gen Sequence Databases were prepared from the whole roots of 14-day-old rice seedlings [11].

The AGO1-associated sRNA HTS data sets were downloaded from GEO. GSM455962, GSM455963 and GSM455964 were prepared from the rice AGO1a, AGO1b and AGO1c proteins, respectively. GSM455965 was prepared from the total protein extract of rice [47].

The rice degradome sequencing data sets (GSM434596, GSM455938, GSM455939 and GSM476257) were obtained from GEO.

The microarray data is a gift from a recent report by Takehisa *et al.*[28]. All the data sets could be obtained from GEO under the accession ID GSE30136. Specifically, the GSM ID for all the used microarray data sets are: GSM746016 (Root cap_rep1), GSM746017 (Root cap_rep2), GSM746018 (Root cap_rep3), GSM746019 (Division zone_rep1), GSM746020 (Division zone_rep2), GSM746021 (Division zone_rep3), GSM746022 (Elongation zone_rep1), GSM746023 (Elongation zone_rep2), GSM746024 (Elongation zone_rep3), GSM746025 (Maturation zone_I_rep1), GSM746026 (Maturation zone_I_rep2), GSM746027 (Maturation zone_I_rep3), GSM746028 (Maturation zone_II_rep1), GSM746029 (Maturation zone_II_rep2), GSM746030 (Maturation zone_II_rep3), GSM746031 (Maturation zone_III_rep1), GSM746032 (Maturation zone_III_rep2), GSM746033 (Maturation zone_III_rep3), GSM746034 (Maturation zone_IV_rep1), GSM746035 (Maturation zone_IV_rep2), GSM746036 (Maturation zone_IV_rep3), GSM746037 (Maturation zone_V_rep1), GSM746038 (Maturation zone_V_rep2) and GSM746039 (Maturation zone_V_rep3). GSM746016, GSM746017, GSM746018, GSM746019, GSM746020 and GSM746021 were used as the target gene expression profiling data of the root tips [the six libraries were prepared from the root tips (200 μm) of rice, which correlates well with the sRNA libraries of the root tips], and all the 24 GSM data sets were used as a whole to investigate the expression levels of the target genes within the whole roots.

The miRNA sequences were obtained from miRBase (release 19; http://www.mirbase.org/) [26]. The transcripts of rice genes and the gene annotations were retrieved from the rice genome annotation project established by the institute for genome research (TIGR rice, release 6.1; http://rice.plantbiology.msu.edu/index.shtml) [29].

### Prediction and validation of the sRNA targets

Target prediction was performed by using miRU algorithm [17,18] with default parameters. The degradome sequencing data were utilized to validate the predicted sRNA—target pairs. First, in order to allow cross-library comparison, the normalized read count (in RPM, reads per million) of a short sequence from a specific degradome library was calculated by dividing the raw count of this sequence by the total counts of the library, and then multiplied by 10^6^. Then, two-step filtering was performed to extract the most likely sRNA—target pairs. During the first step, the predicted sRNA binding sites along with the 50-nt surrounding sequences at both ends were collected in order to reduce the BLAST time. For the BLAST, all the collected degradome data sets were utilized at the same time to do a comprehensive search. It was based on the scenario that a sRNA—target pair was considered to be the candidate once the cleavage signal(s) existed in any data set(s). The predicted targets met the following criteria were retained: (1) there must be perfectly matched degradome signatures with their 5′ ends resided within 8^th^ to 12^th^ nt region from the 5′ ends of the regulating sRNAs; and (2) for a specific position within the 8^th^ to 12^th^ nt region, which could be regarded as the potential cleavage site, there must be two or more distinct degradome signatures with accordant 5′ ends to support this position. These retained transcripts were subjected to a second BLAST, and the degradome signals along each transcript were obtained to provide a global view of the signal noise when compared to the signal intensity within a specific target binding site. Referring to our previous study [21], both global and local t-plots were drawn. Finally, exhaustive manual filtering was performed, and only the transcripts with cleavage signals easy to be recognized were extracted as the potential sRNA—target pairs.

### Availability of supporting data

The data sets supporting the results of this article are included within the article and its additional files.

## Abbreviations

miRNA: MicroRNA; sRNA: Small RNA; AGO1: Argonaute 1; ARF: *Auxin response factor*; HTS: High-throughput sequencing; RPM: Reads per million; RISC: RNA-induced silencing complex; U: Uridine; nt: Nucleotide; TIGR: The Institute for Genome Research; TAIR: The *Arabidopsis* information resource; MAX2: *More axillary growth 2*; GLR: Glutamate receptor; GEO: Gene expression omnibus.

## Competing interests

The authors declare that they have no competing interests.

## Authors’ contributions

Conceived and designed the experiments: YM, CS, HW, YJ. Performed the experiments: XM, CS. Analyzed the data: XM, CS, YM. Contributed reagents/materials/analysis tools: XM, CS, YM. Wrote the paper: XM, YM, HW, YJ. All authors read and approved the final manuscript.

## Supplementary Material

Additional file 1 **Data S1.** List of sRNAs highly expressed in the root tips of rice. Click here for file

Additional file 2 **Data S2.** List of sRNAs highly expressed in the whole roots of rice. Click here for file

Additional file 3 **Data S3.** AGO1-enriched sRNAs highly expressed in the root tips of rice. Click here for file

Additional file 4 **Data S4.** AGO1-enriched sRNAs highly expressed in the whole roots of rice. Click here for file

Additional file 5: Figure S1 Degradome sequencing data-based validation of the targets of the AGO1-enriched sRNAs highly expressed in the root tips of rice. Click here for file

Additional file 6: Figure S2 Degradome sequencing data-based validation of the targets of the AGO1-enriched sRNAs highly expressed in the whole roots of rice. Click here for file

Additional file 7: Figure S3 Network mediated by the AGO1-enriched sRNAs highly expressed in the root tips of rice. Click here for file

Additional file 8: Figure S4 Network mediated by the AGO1-enriched sRNAs highly expressed in the whole roots of rice. Click here for file

Additional file 9: Table S1 Microarray data-based investigation of the expression patterns of the target genes. Click here for file
