# Supplementary figures and images for: Construction of small RNA-mediated gene regulatory networks in the roots of rice (Oryza sativa)
Source: BMC Genomics. 2013 Jul 27;14:510. doi: 10.1186/1471-2164-14-510 (PMC3734165; doi:10.1186/1471-2164-14-510)

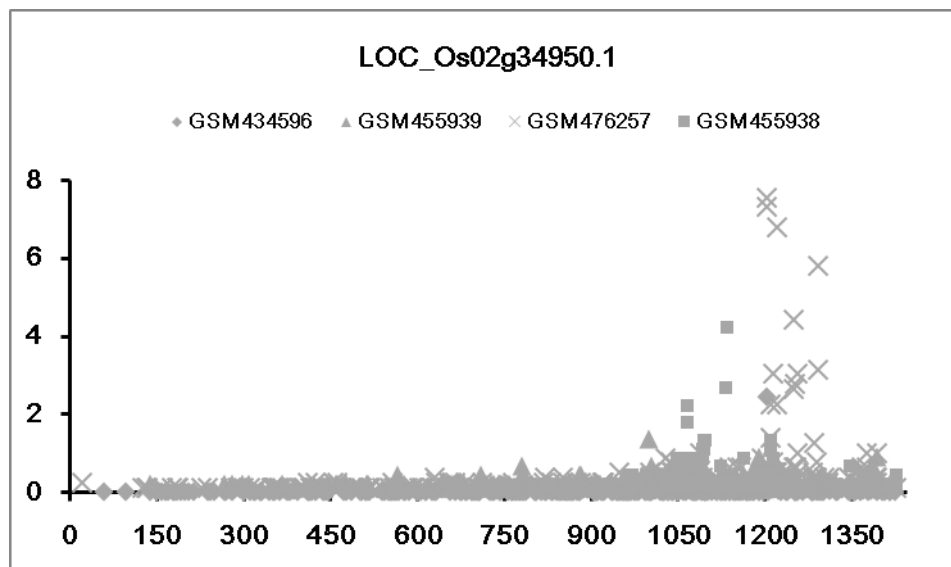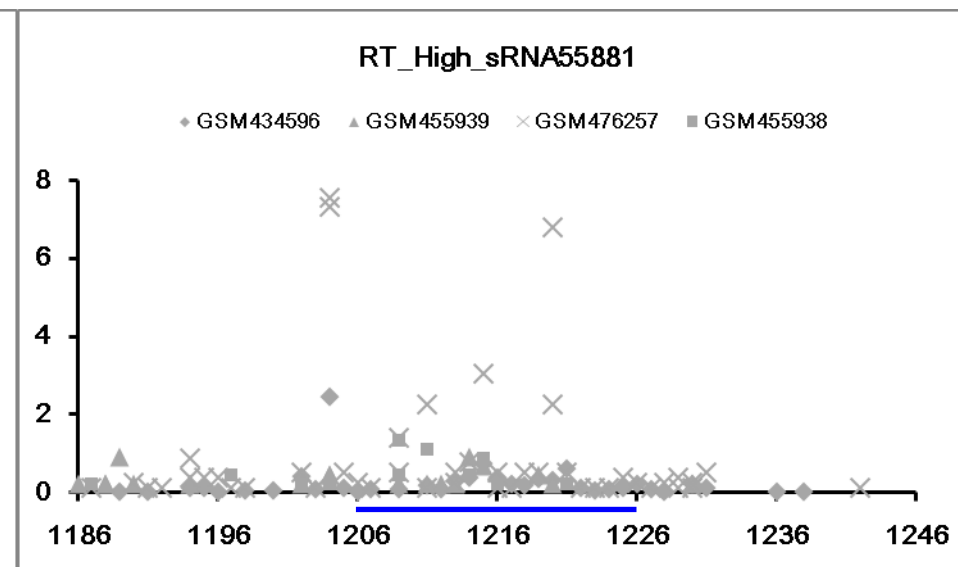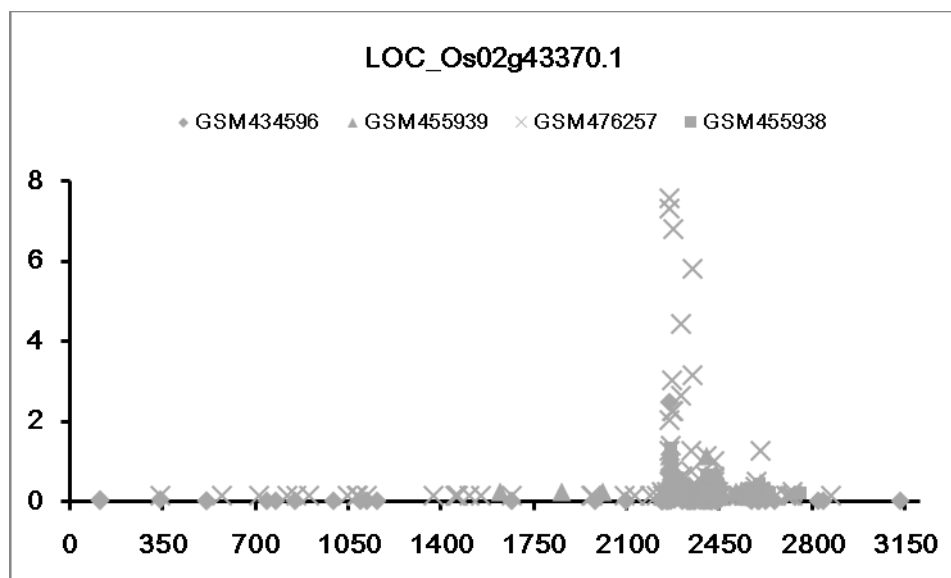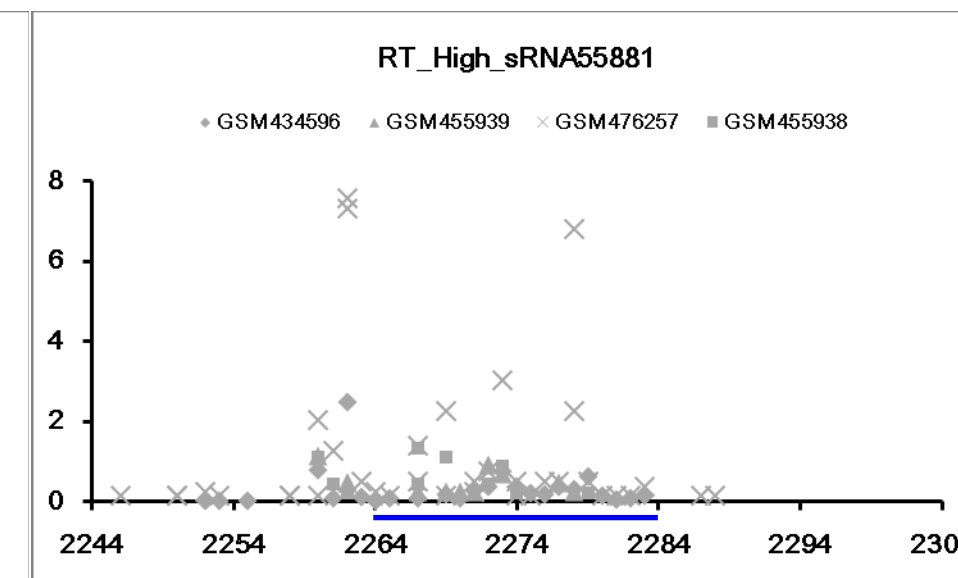

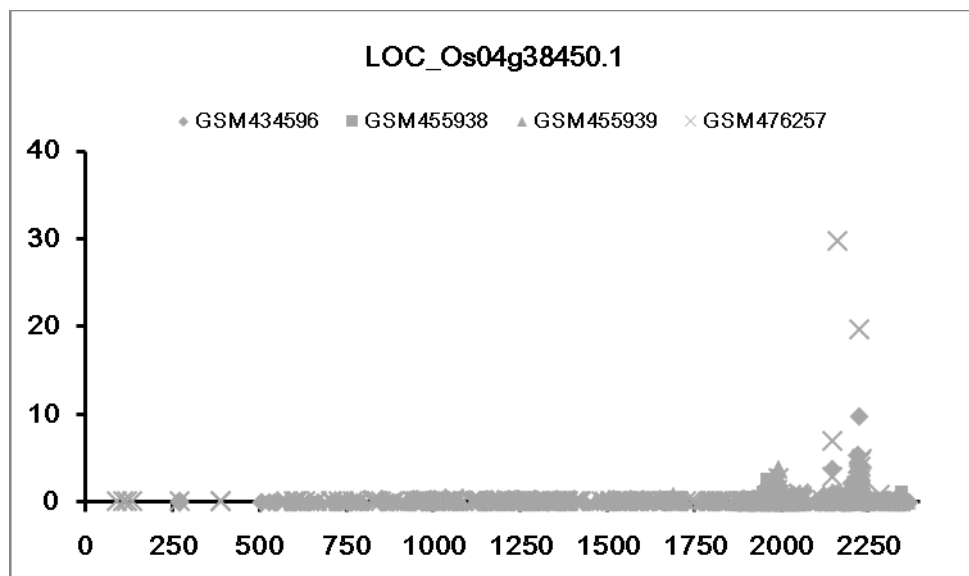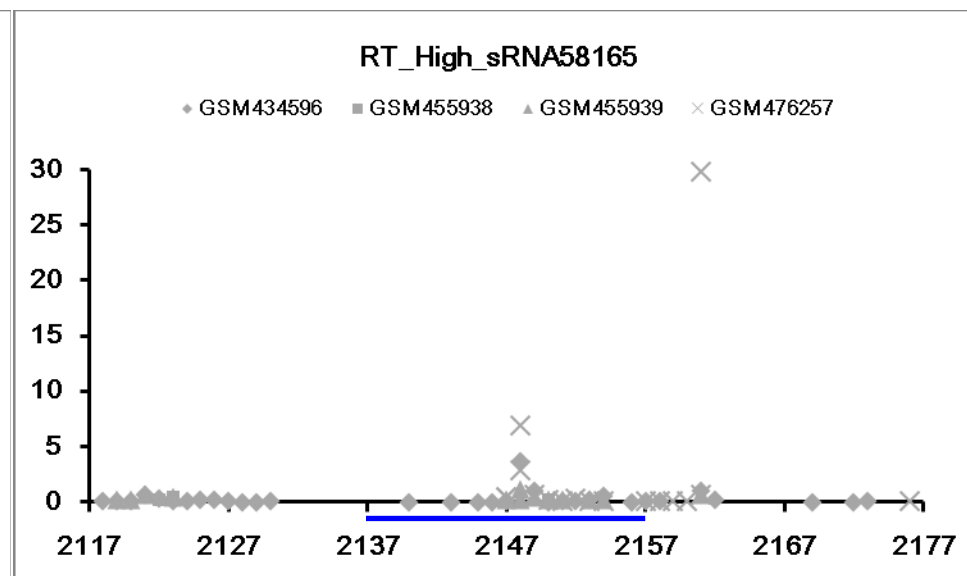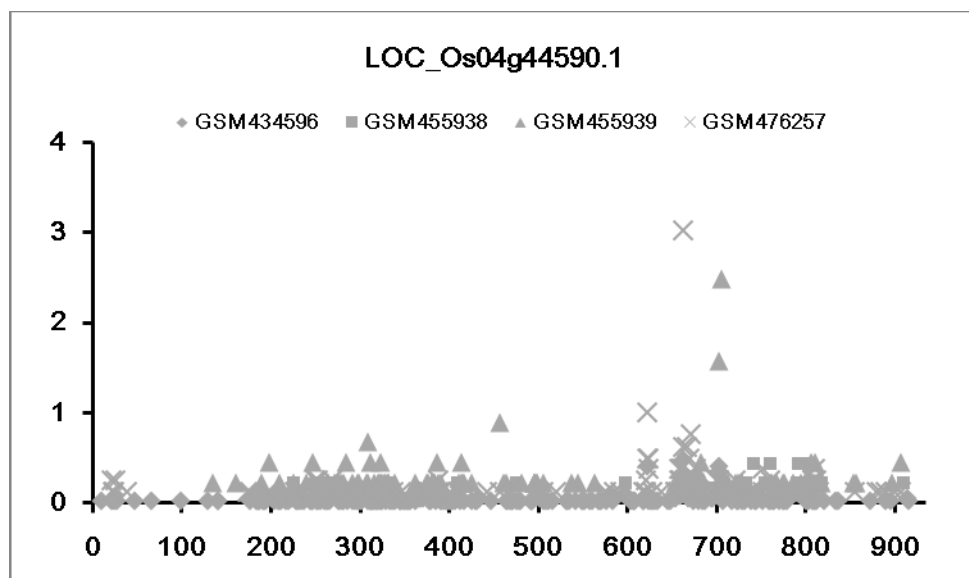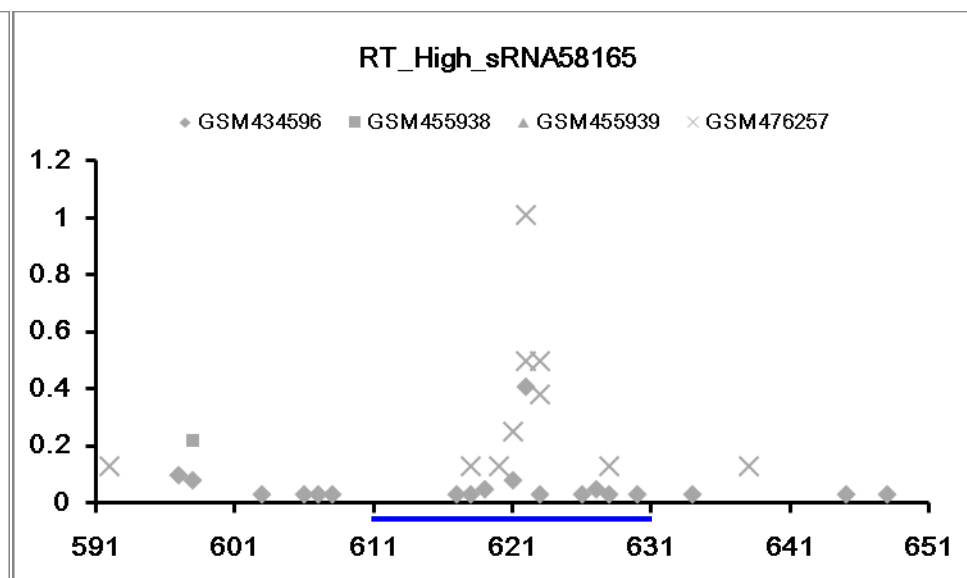

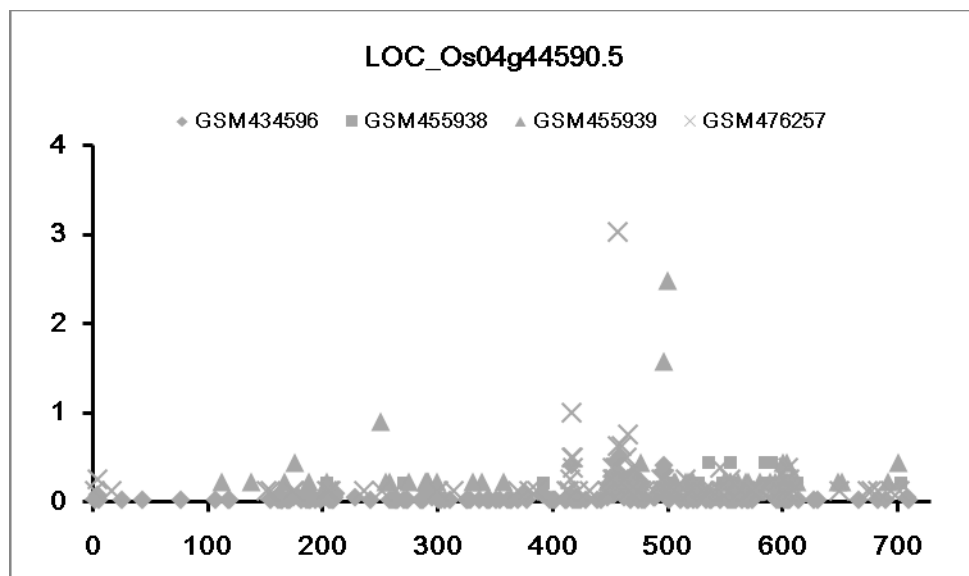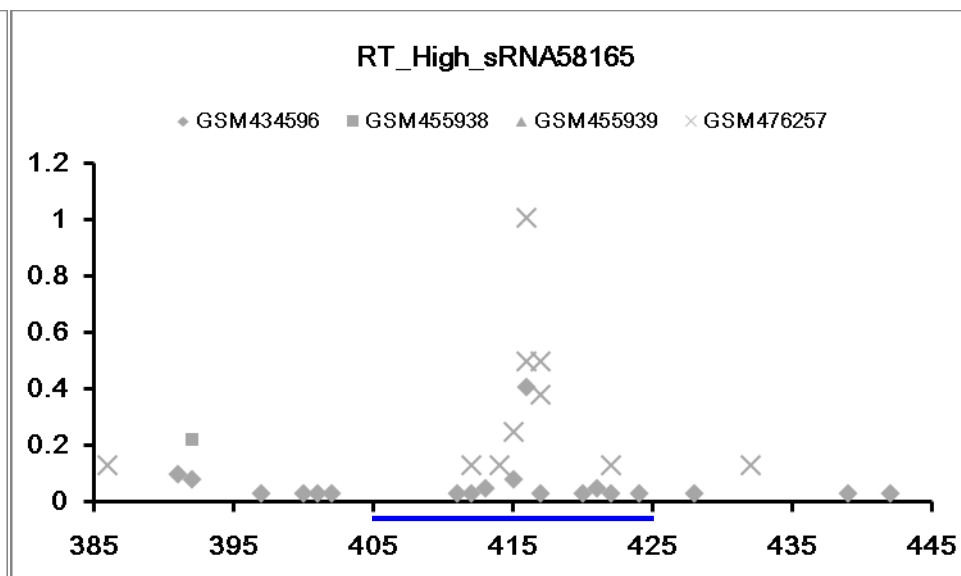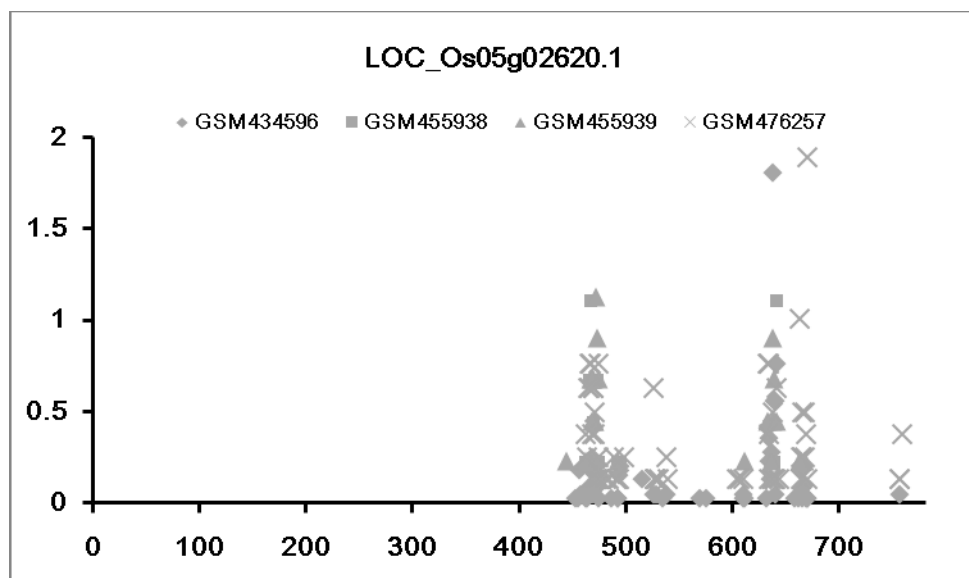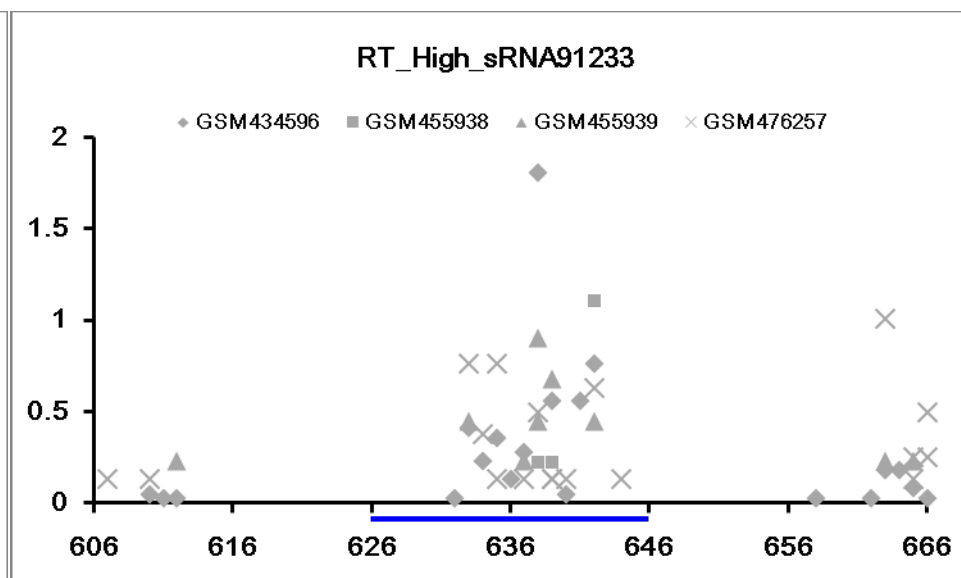

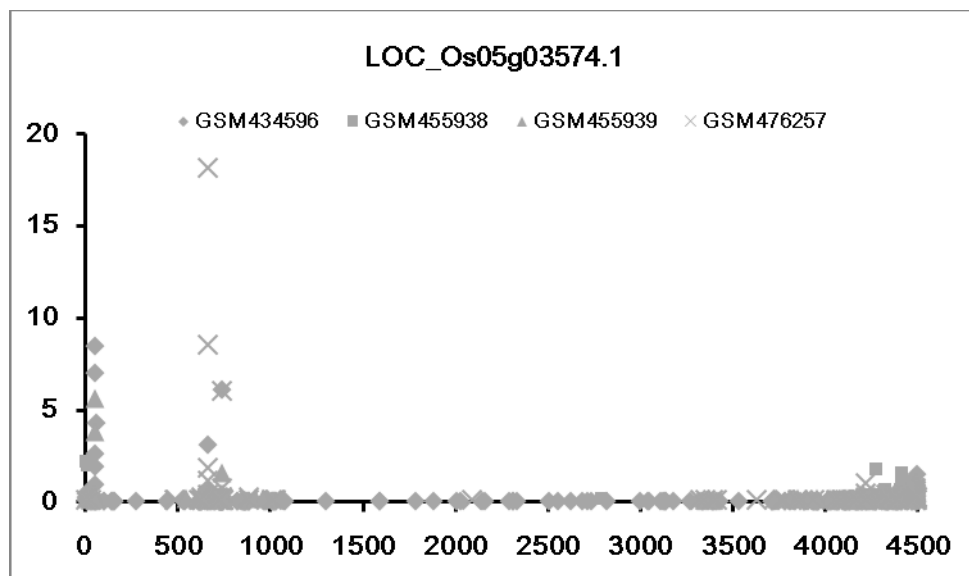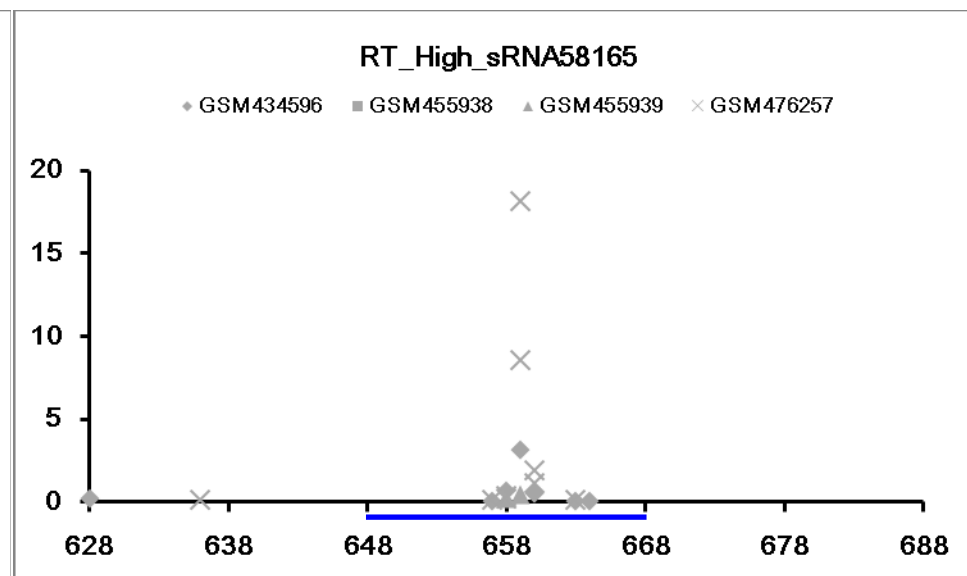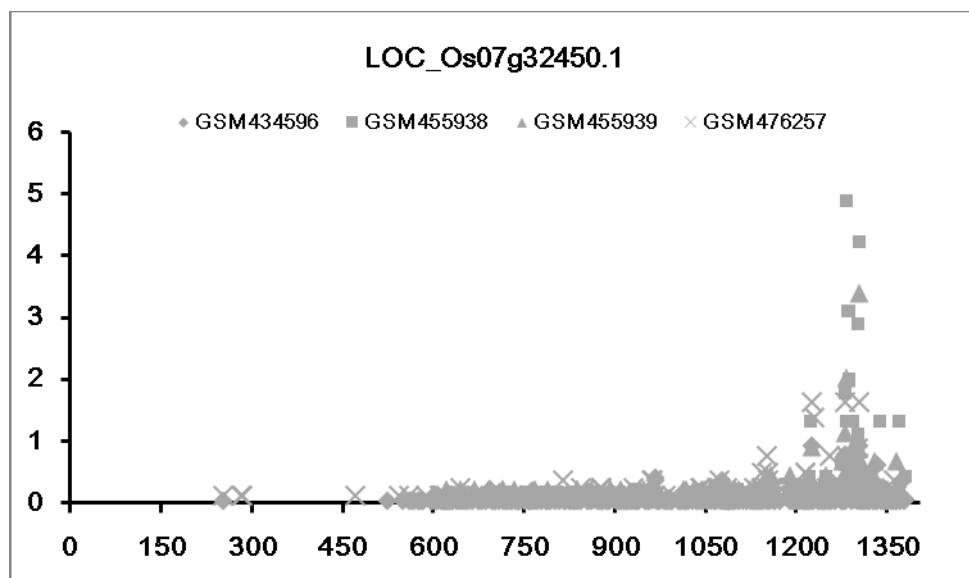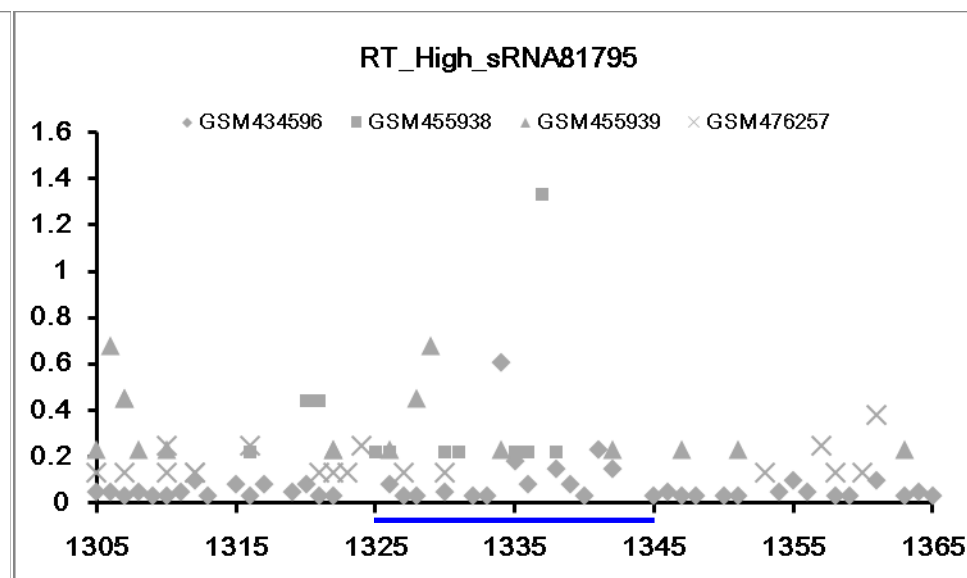

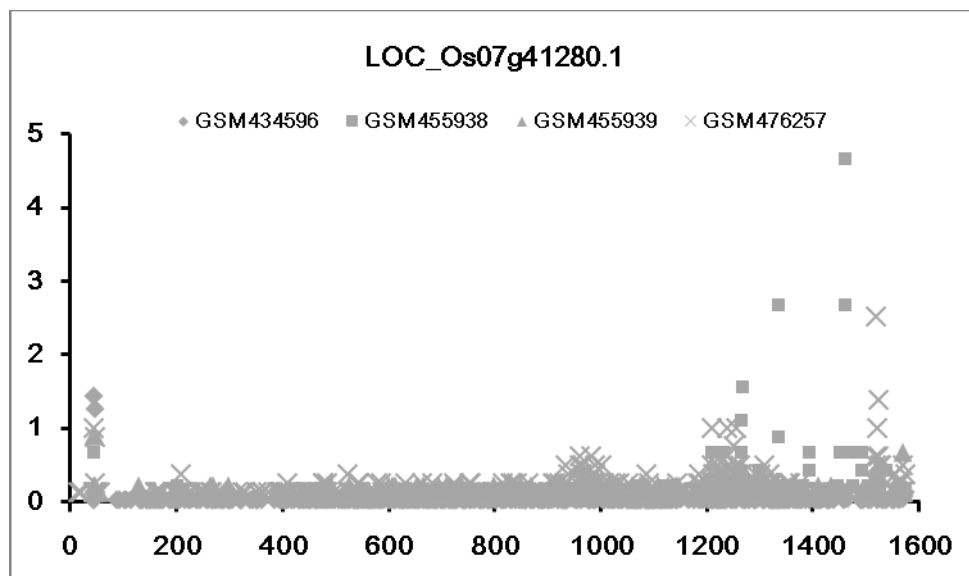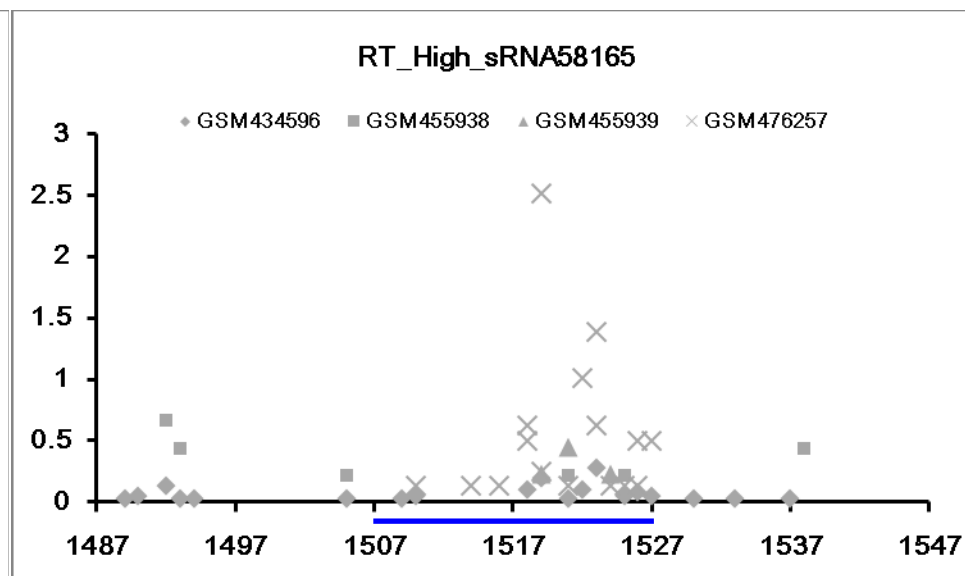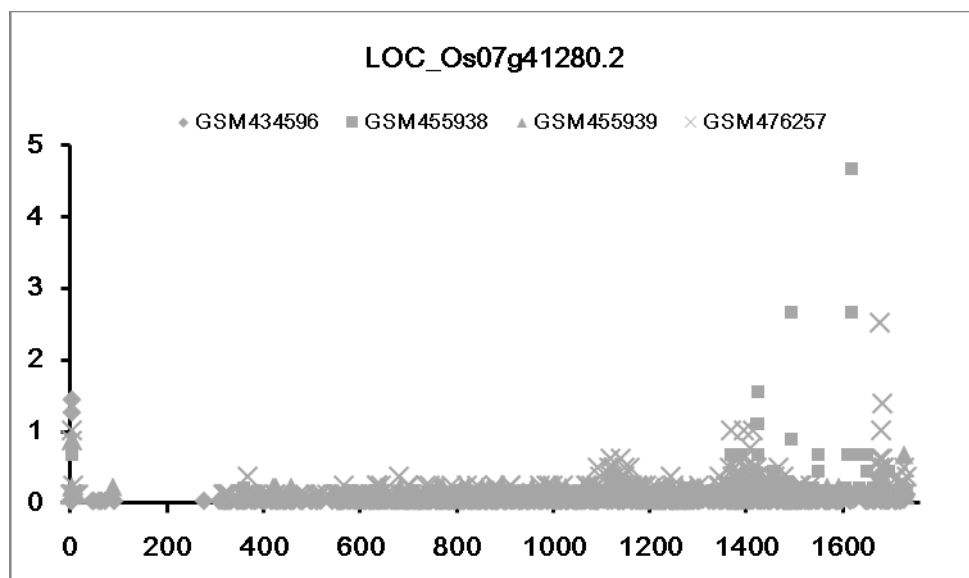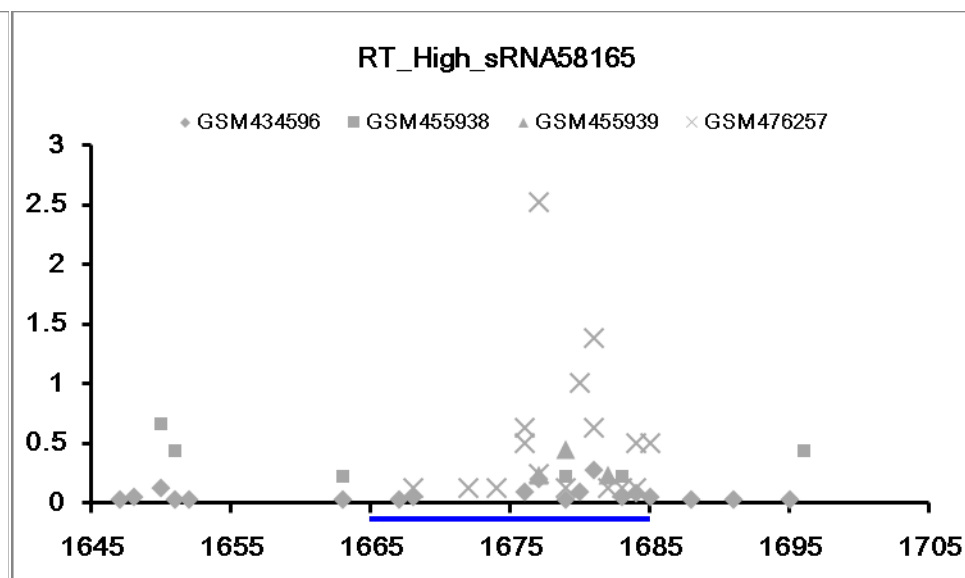

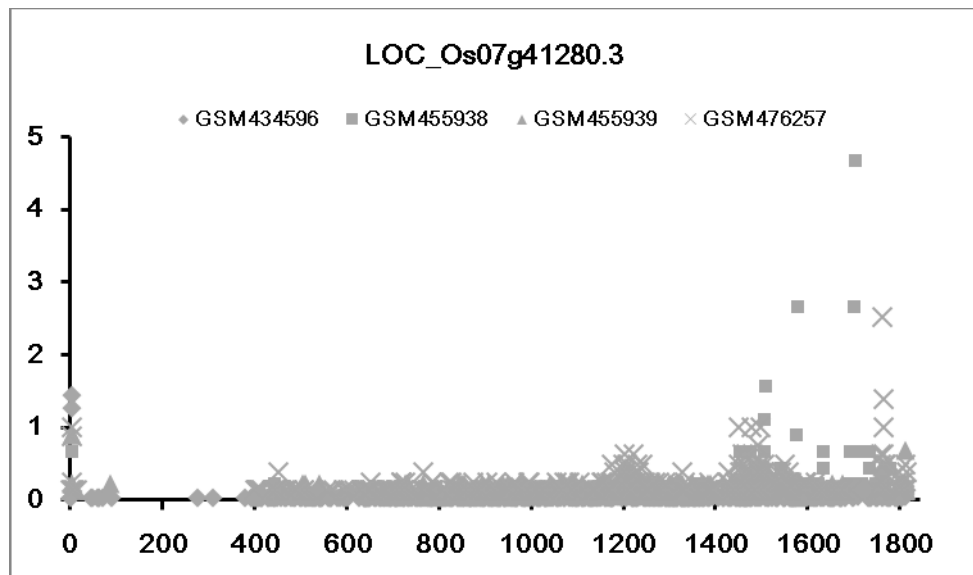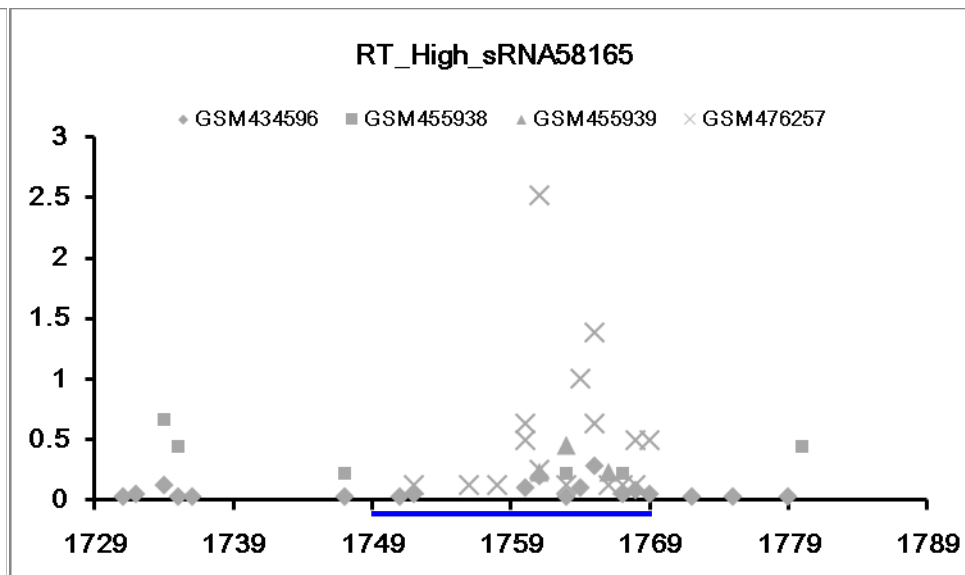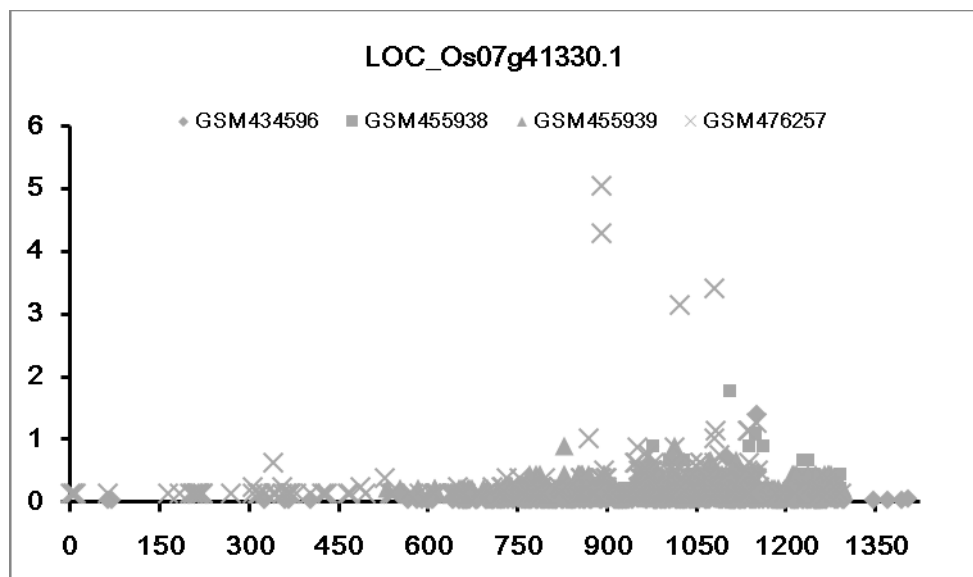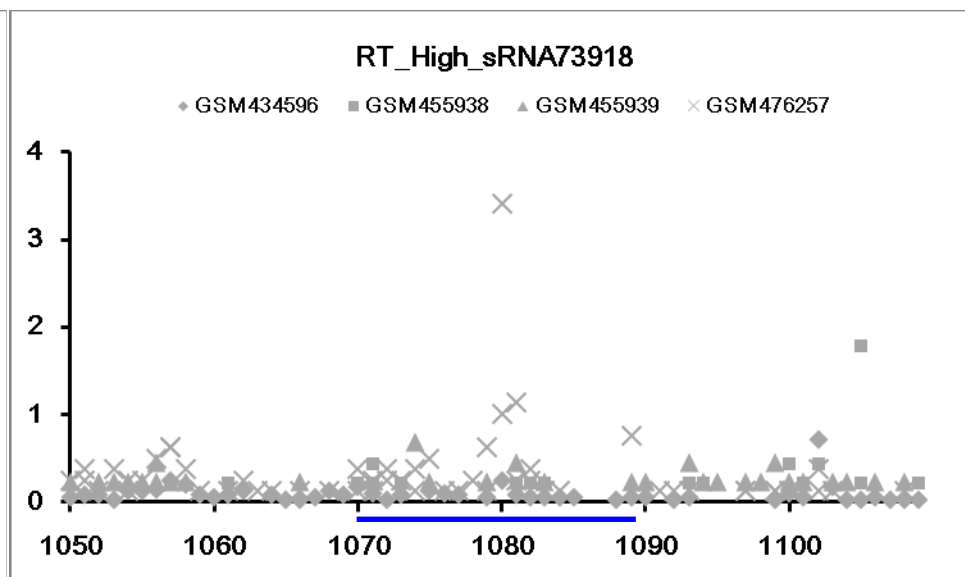

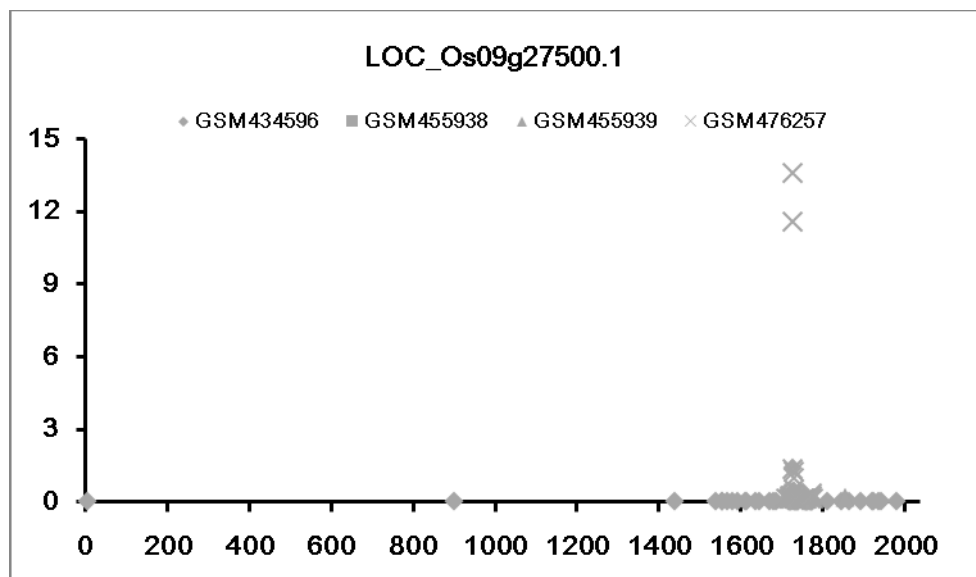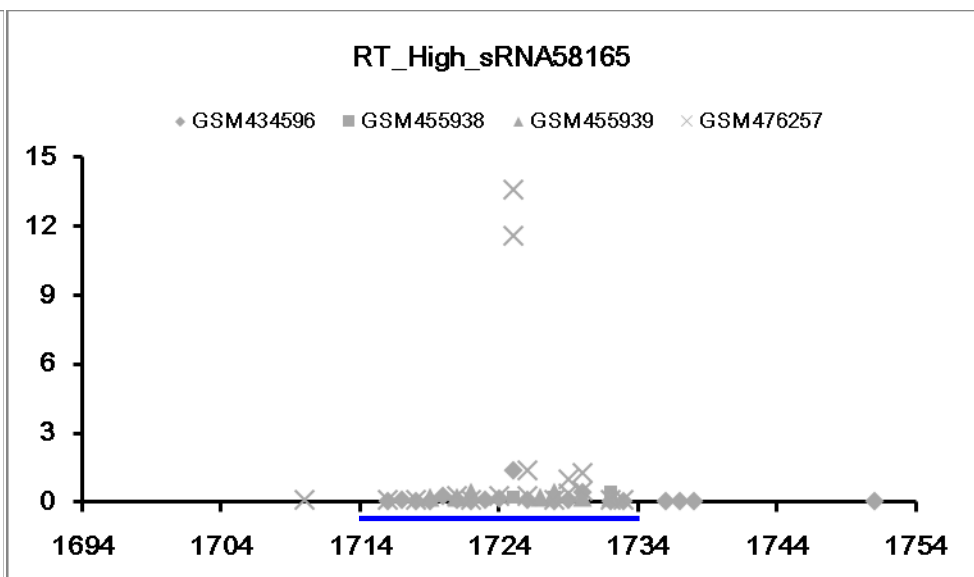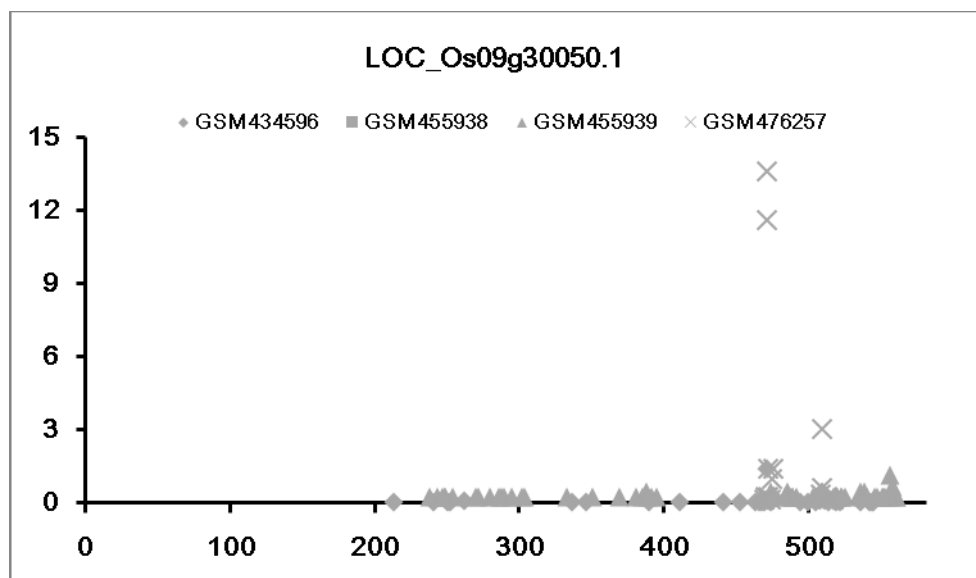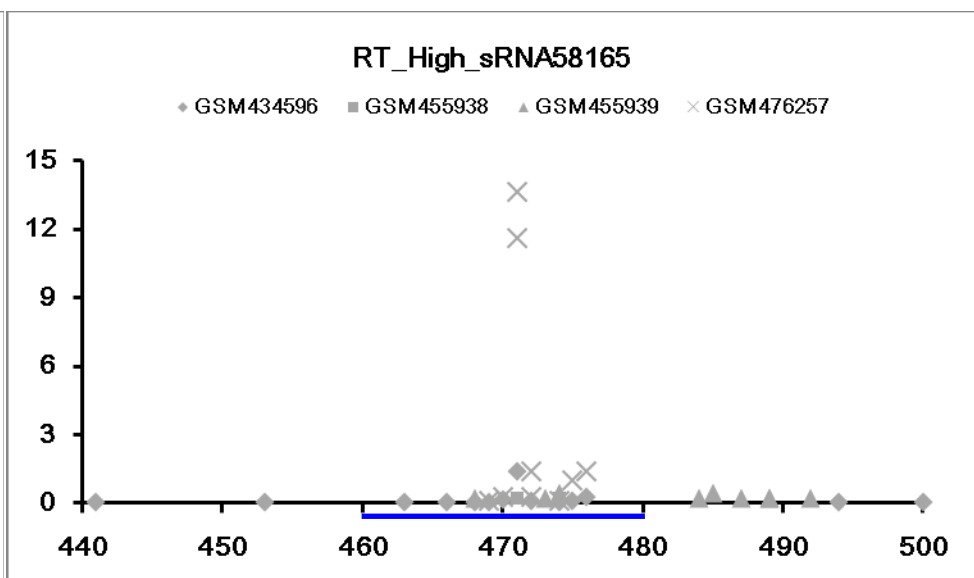



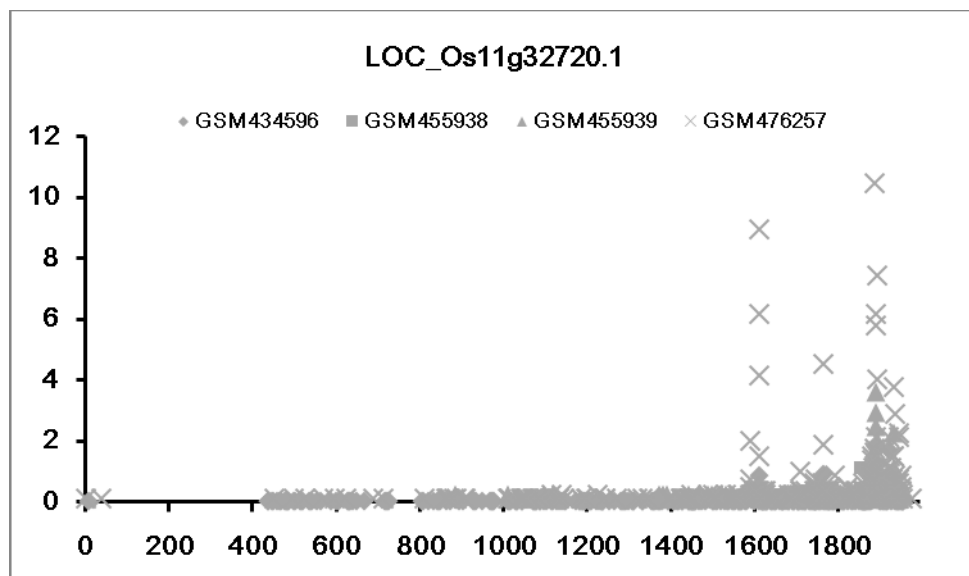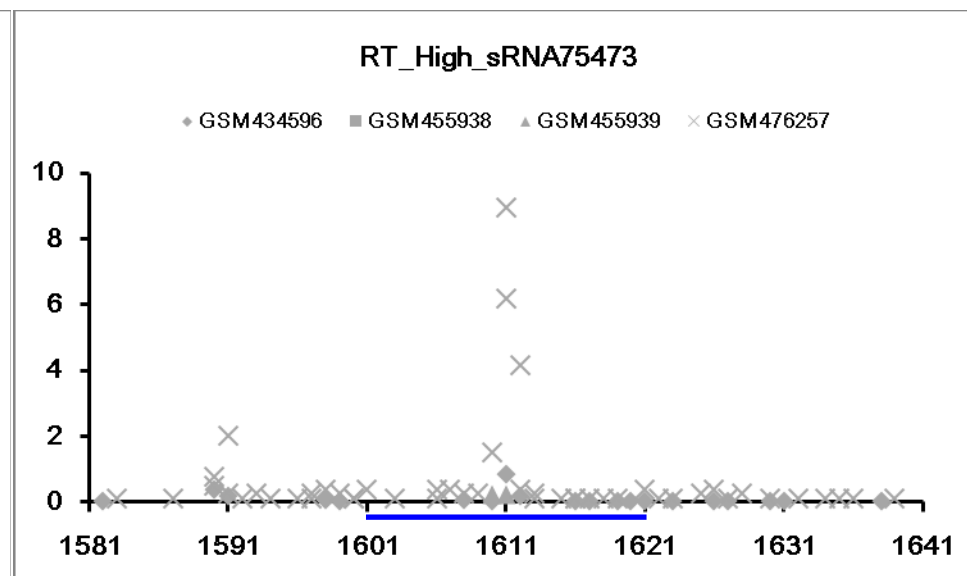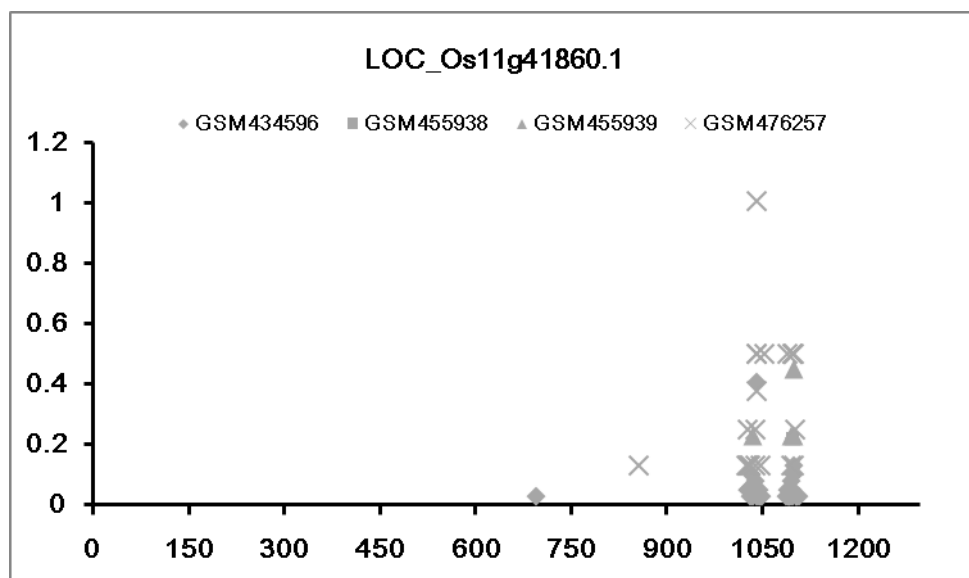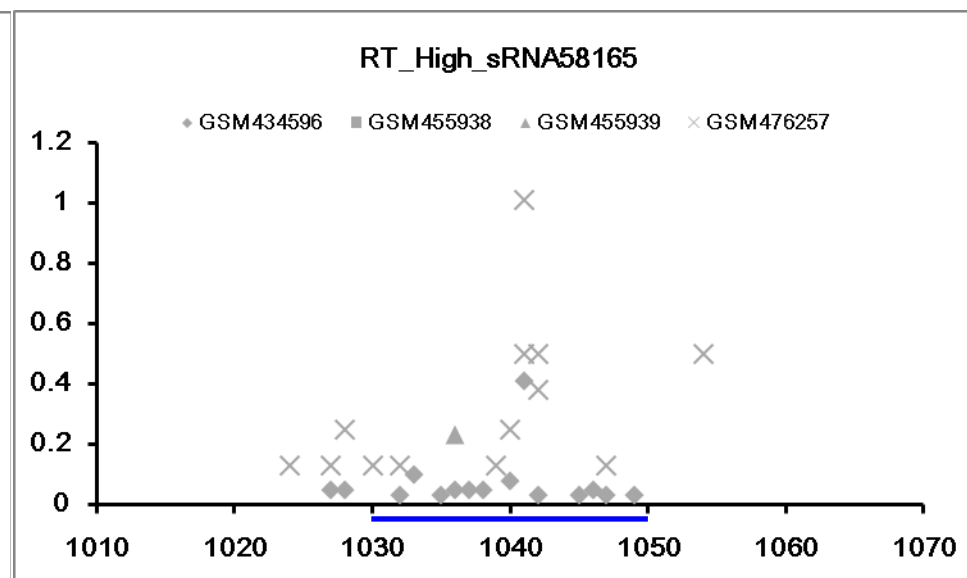

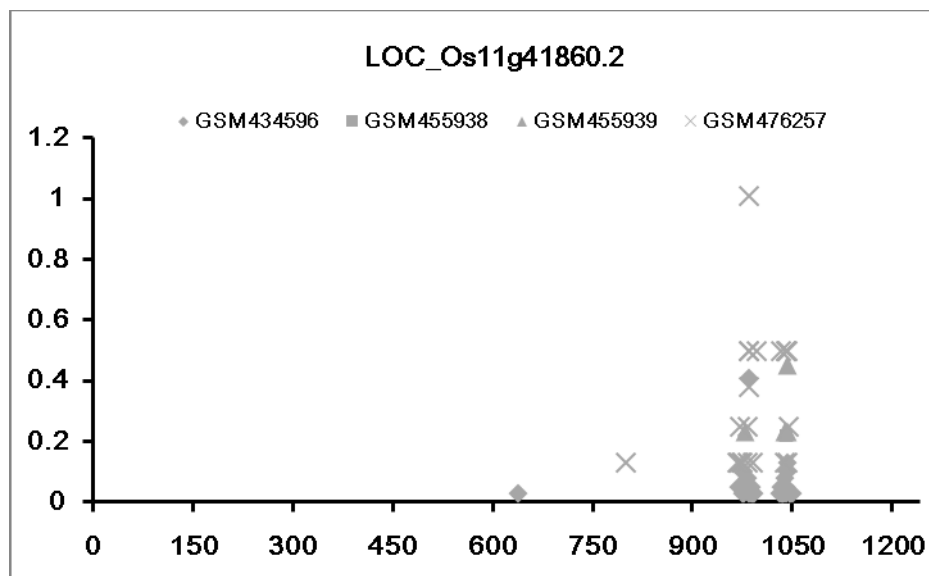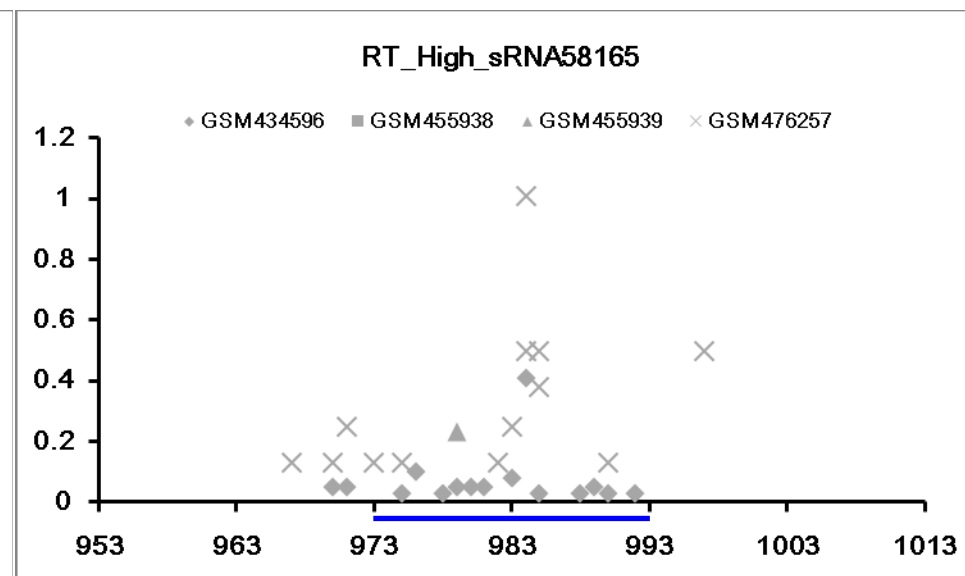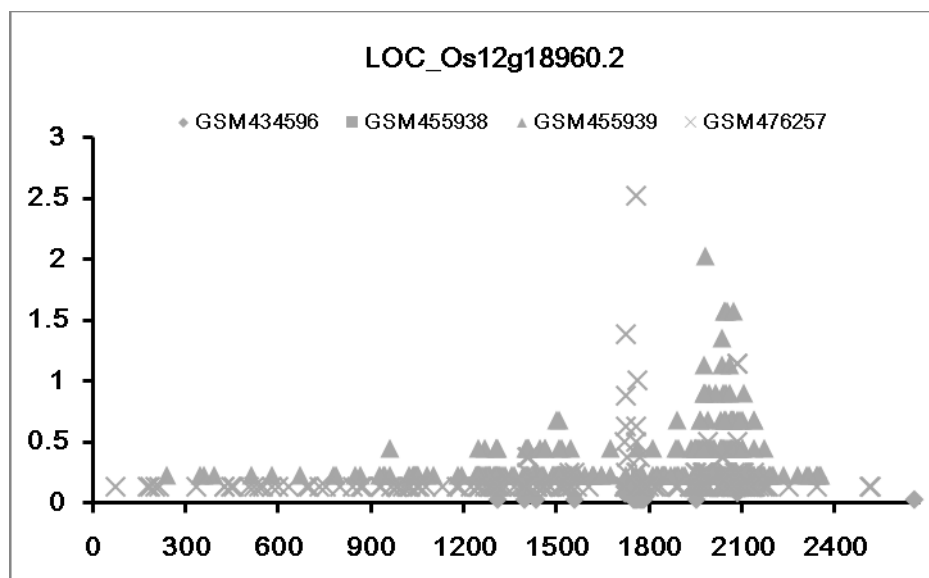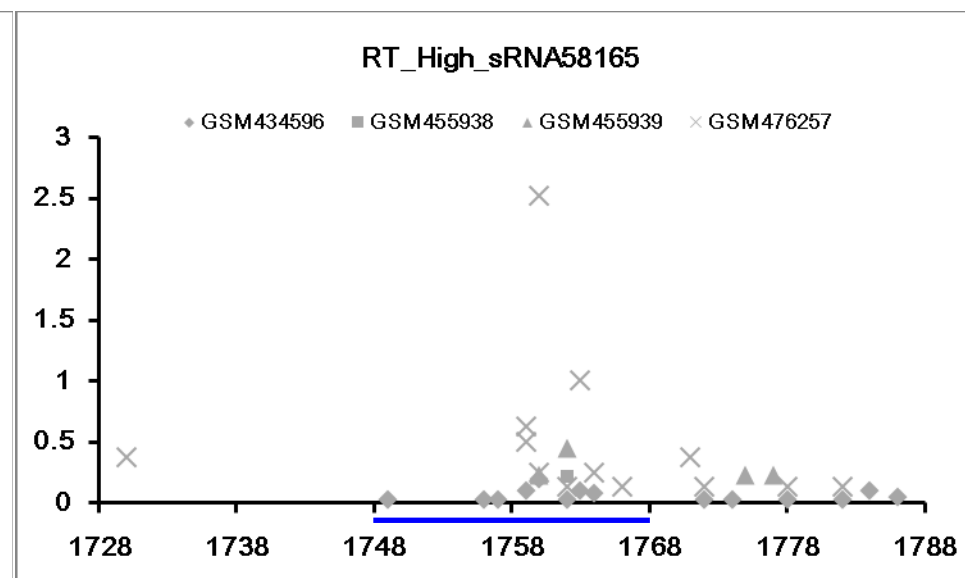

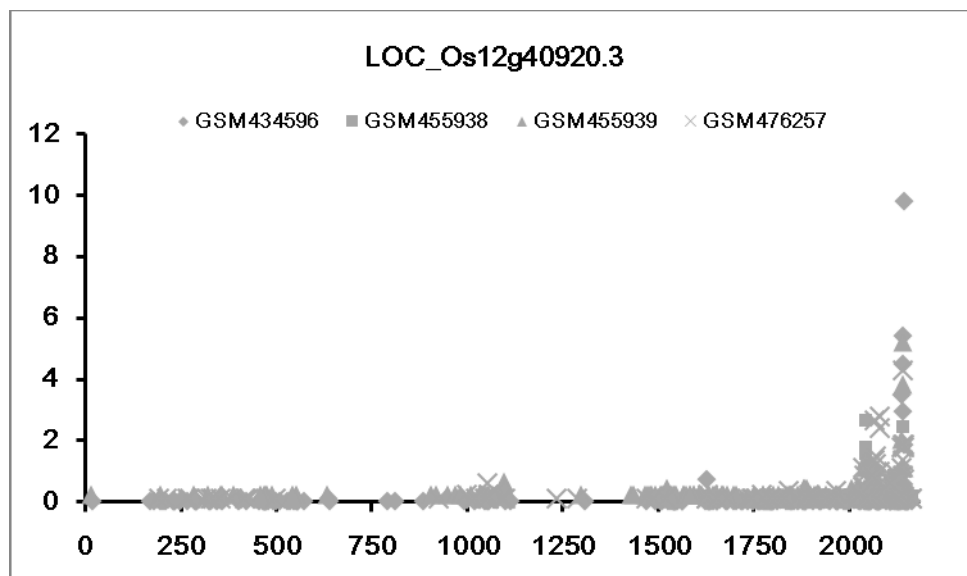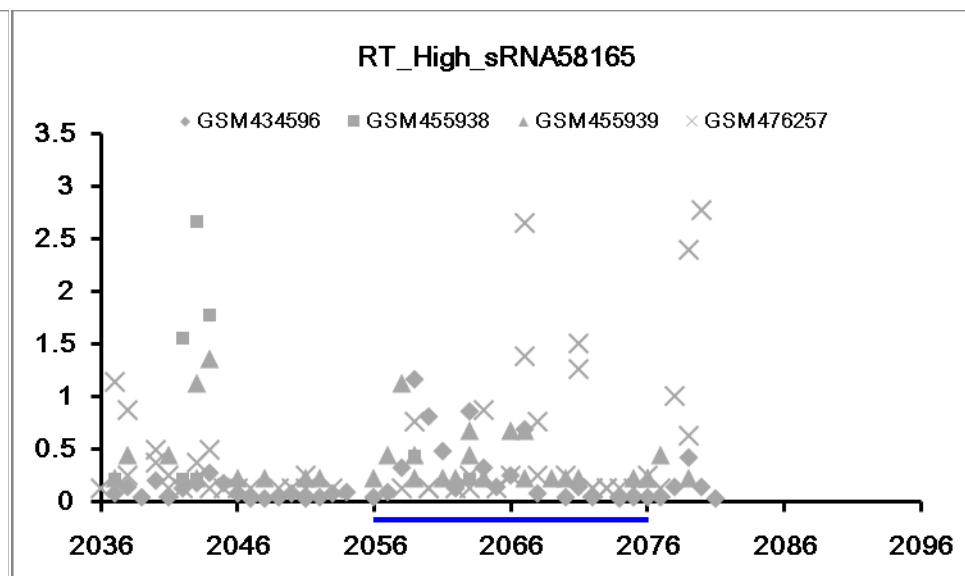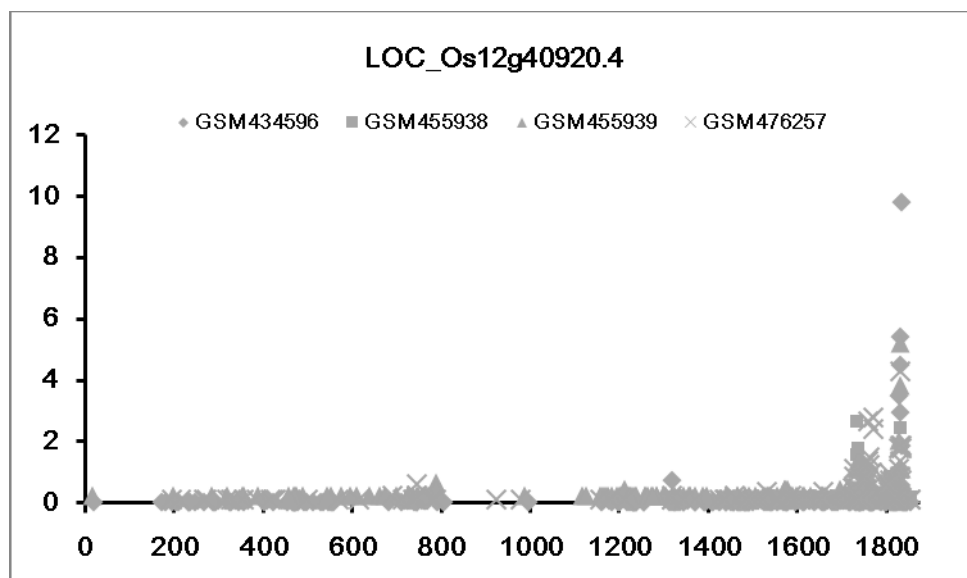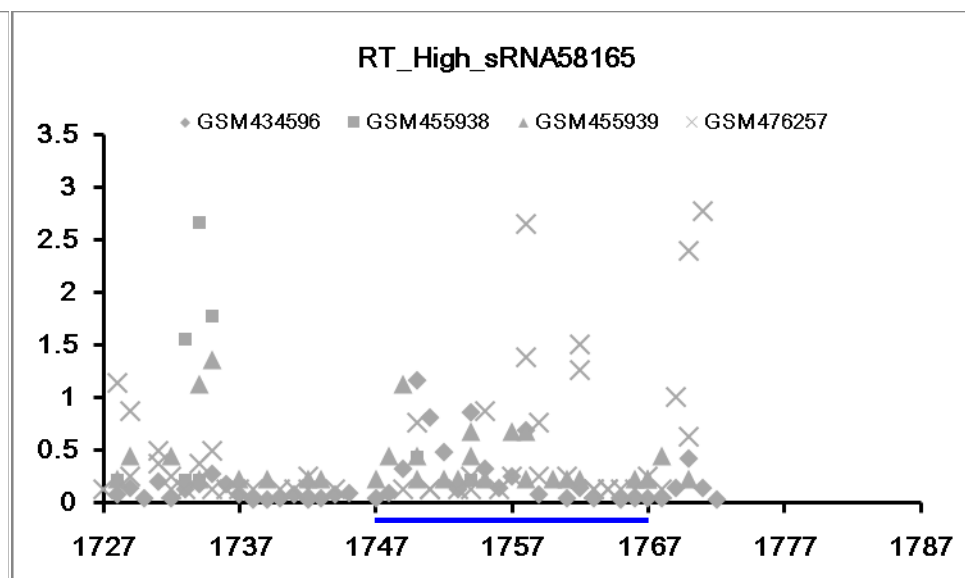

Supplement: Additional file 5: Figure S1 — Degradome sequencing data-based validation of the targets of the AGO1-enriched sRNAs highly expressed in the root tips of rice. [file 1471-2164-14-510-S5.pdf]

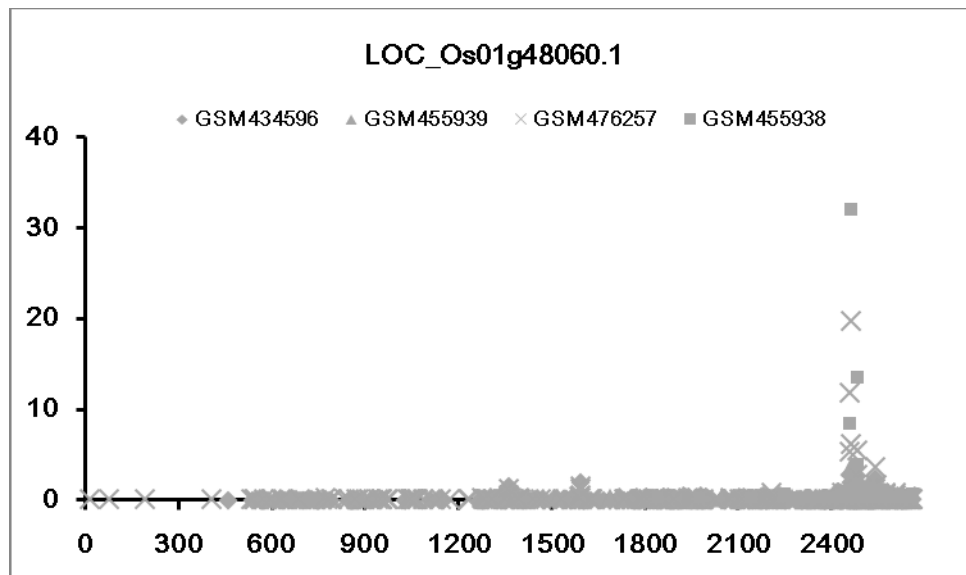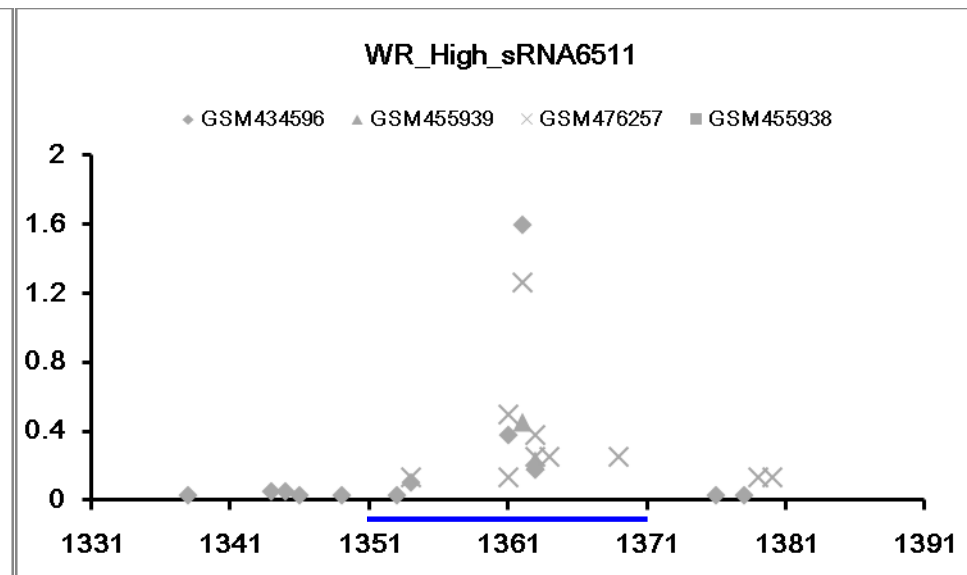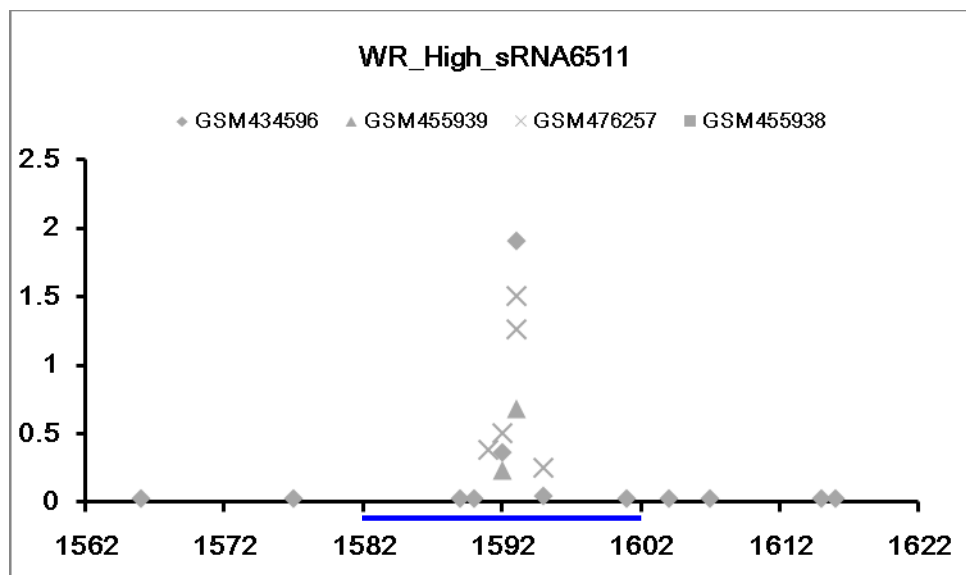

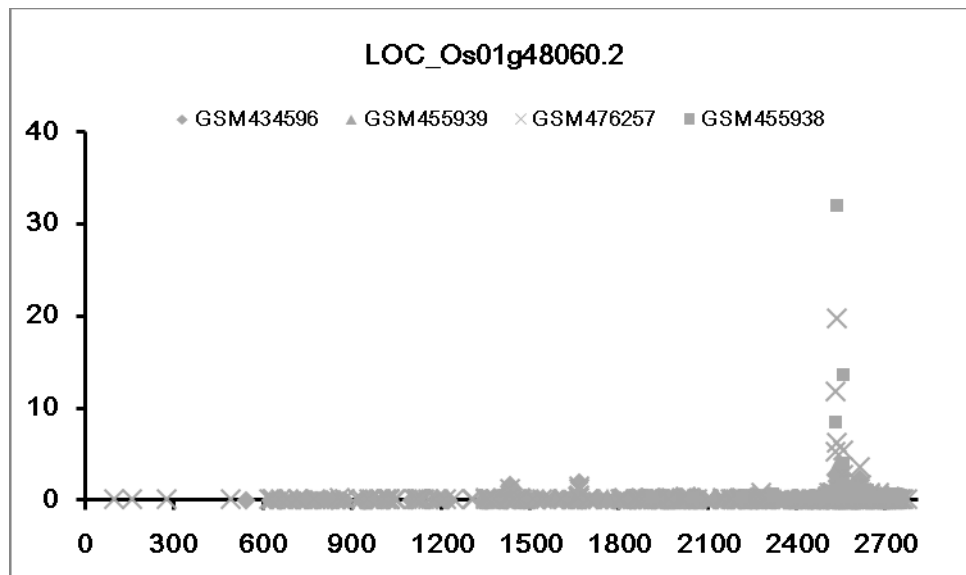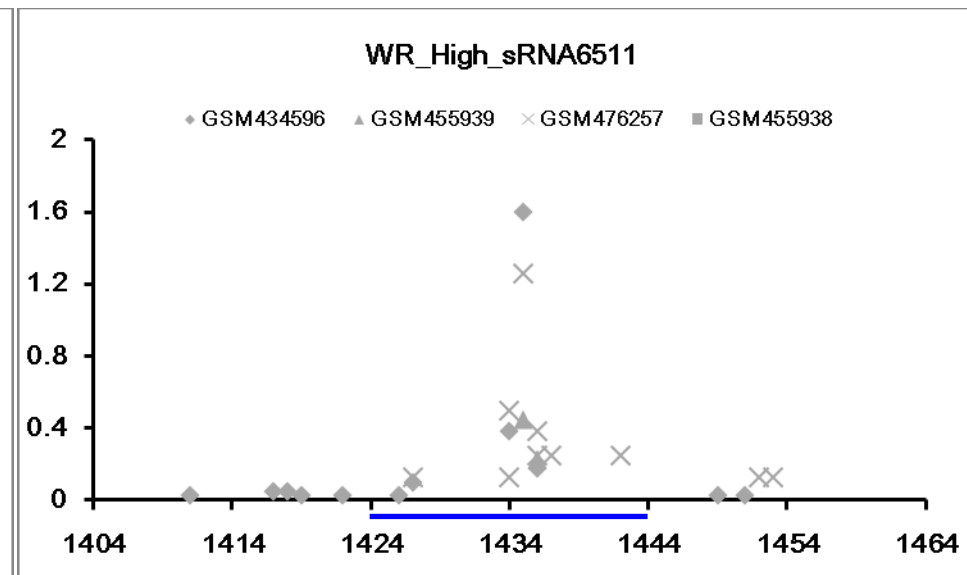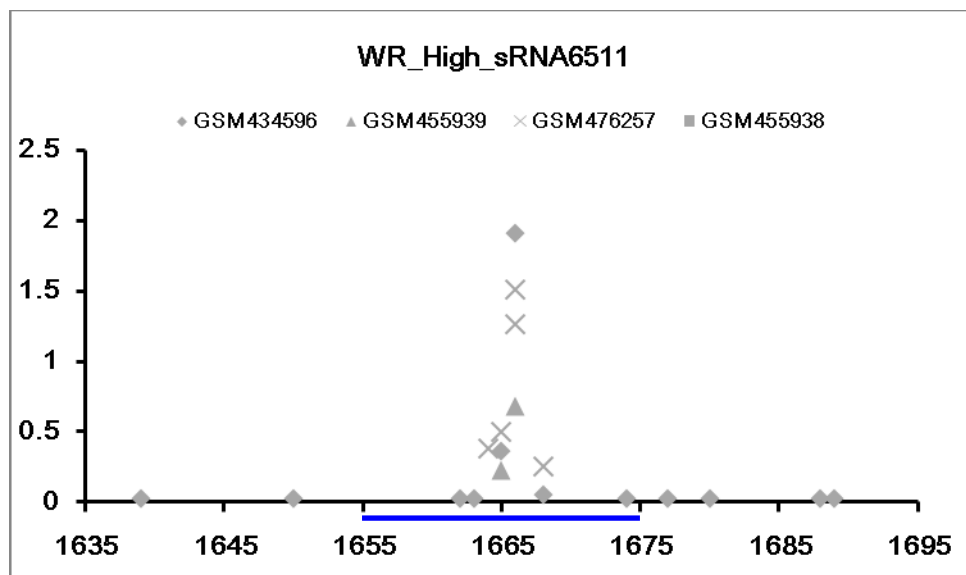

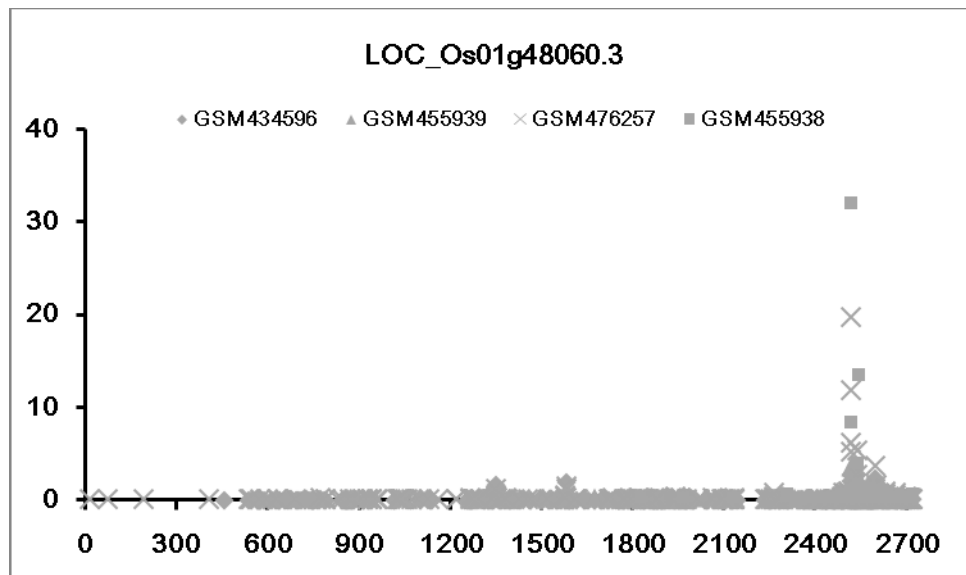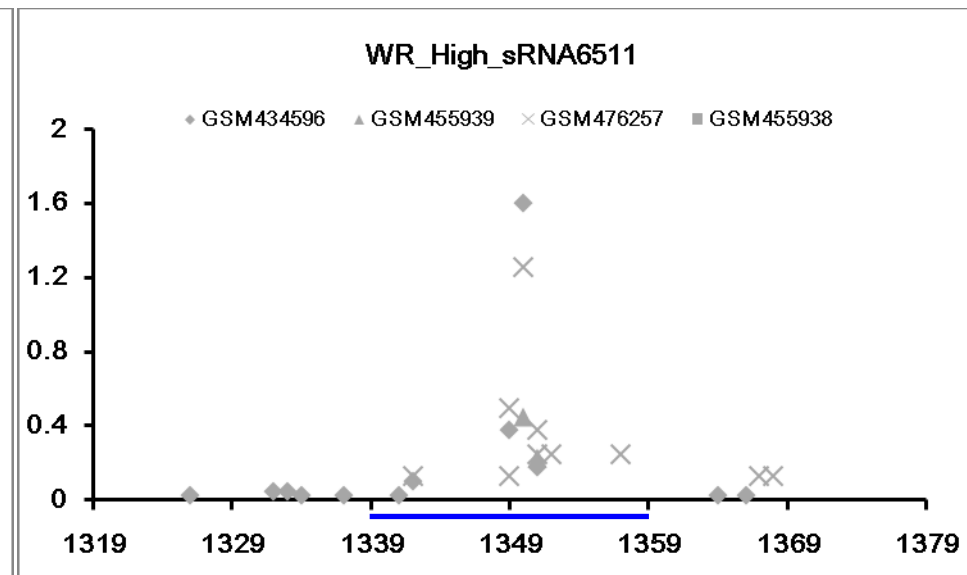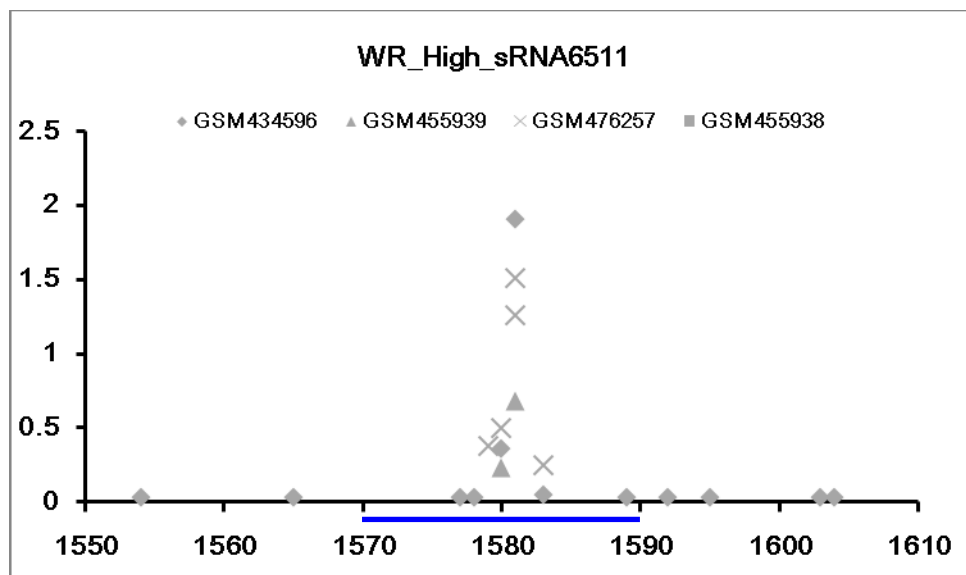

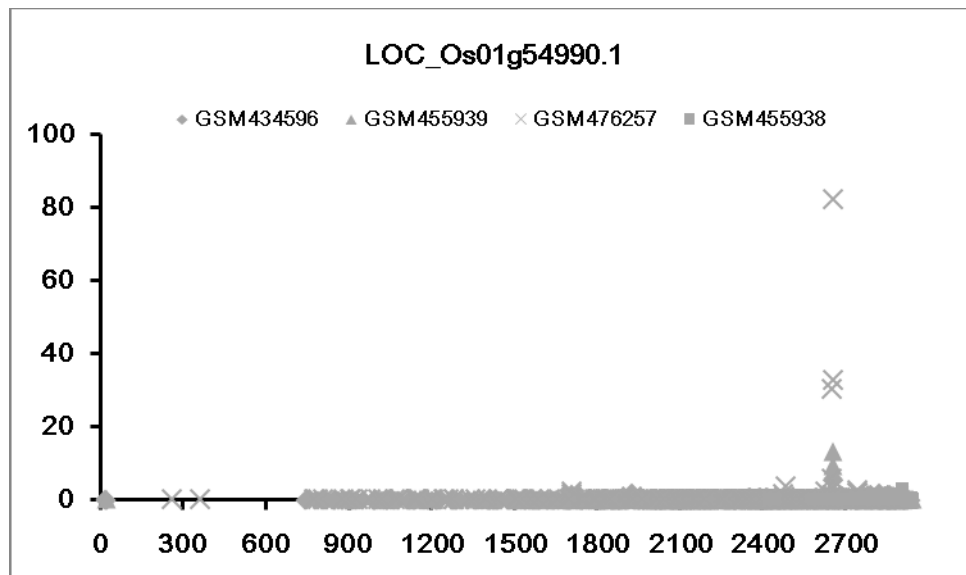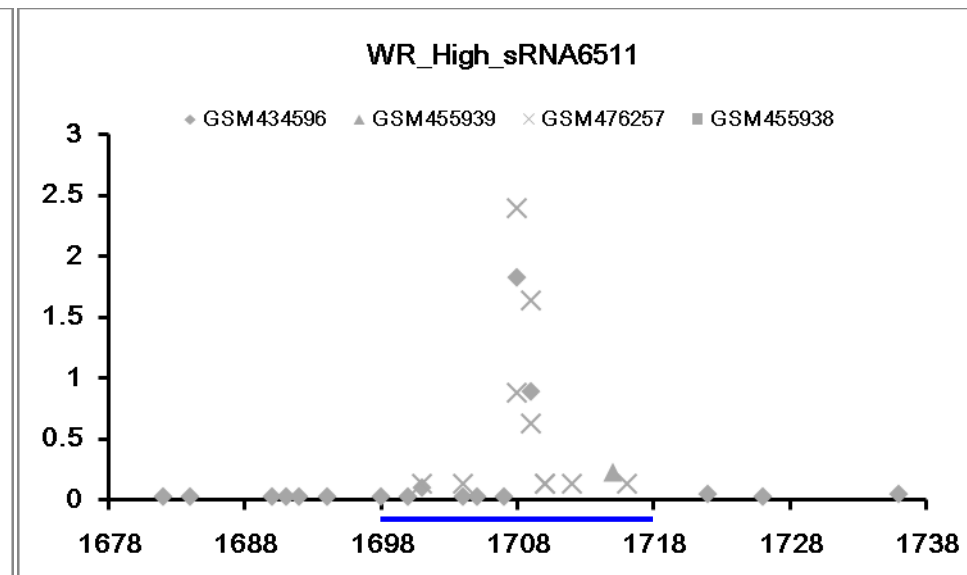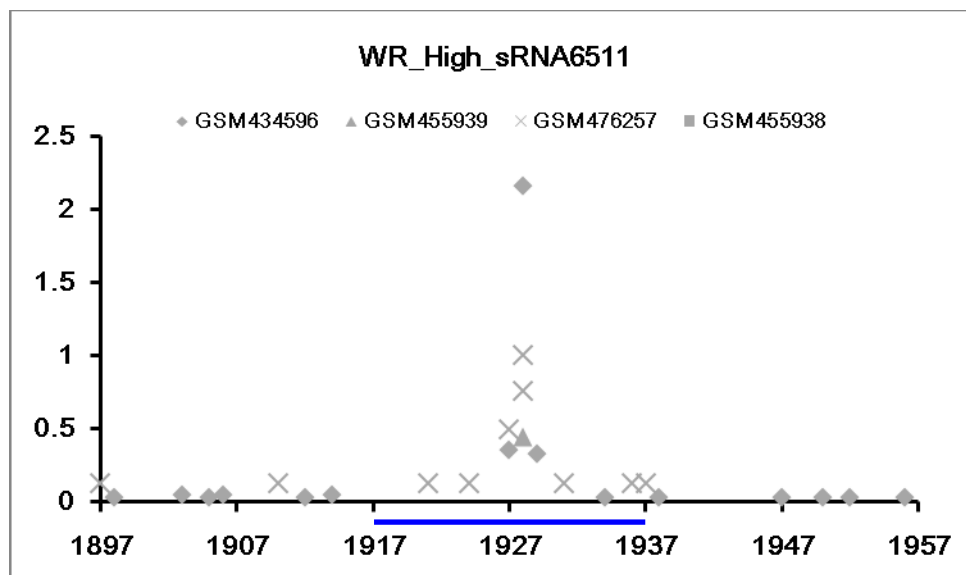

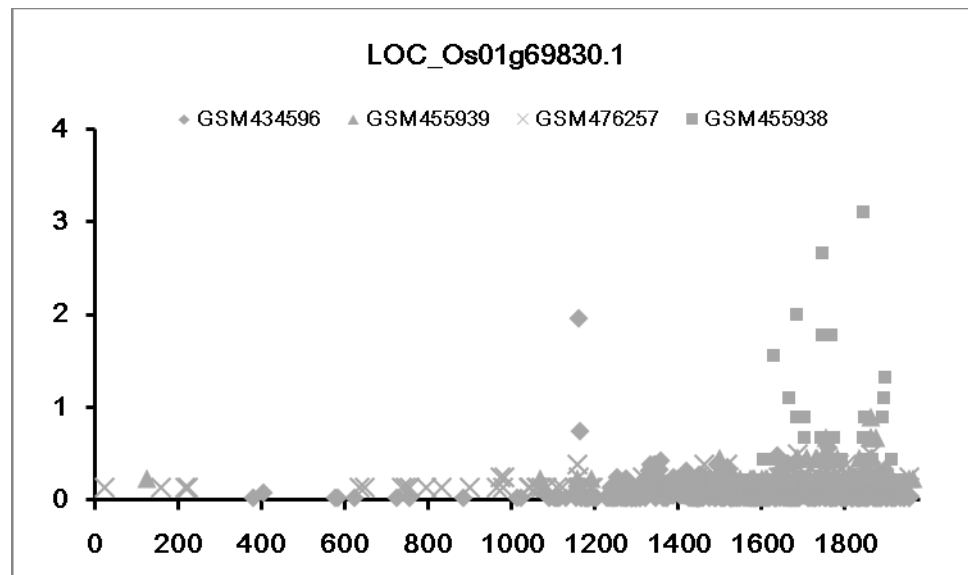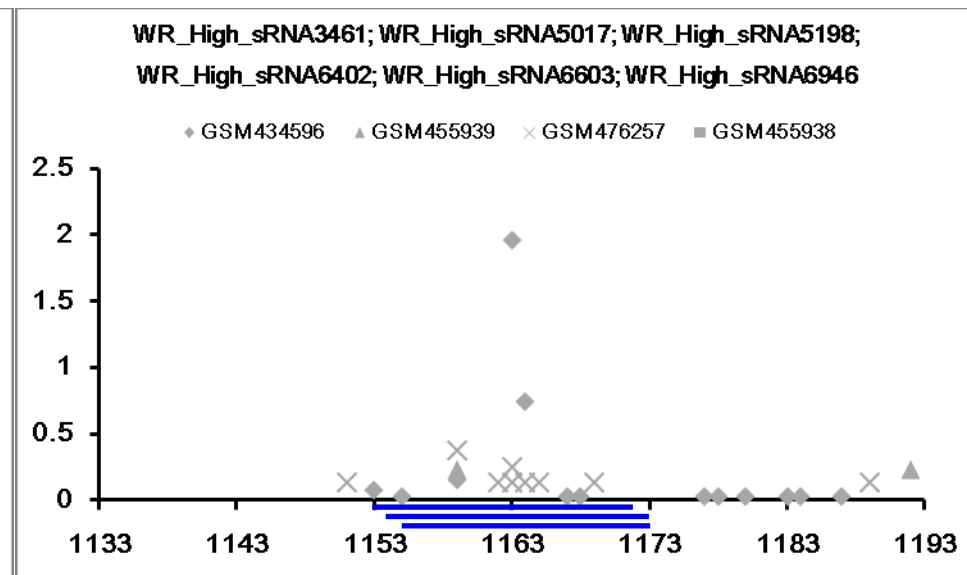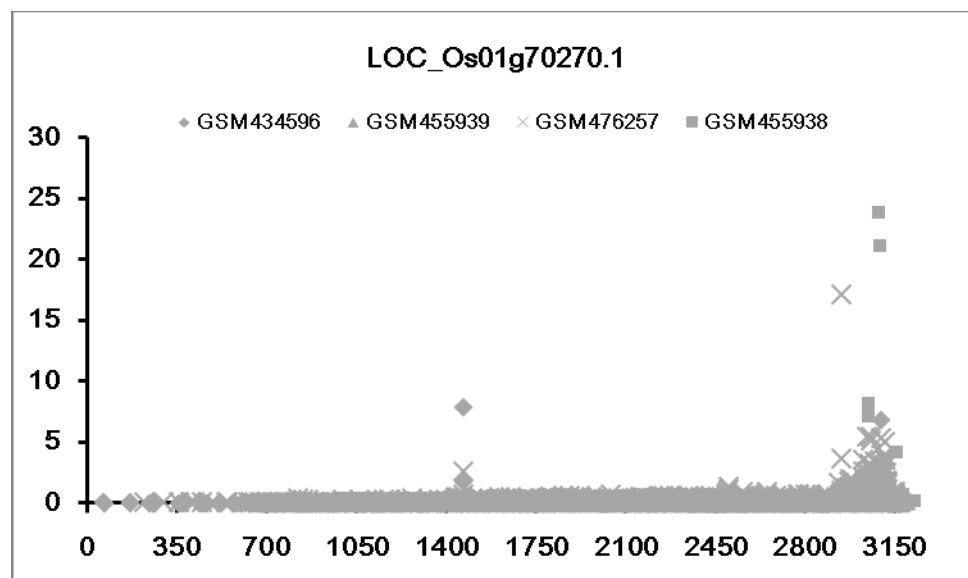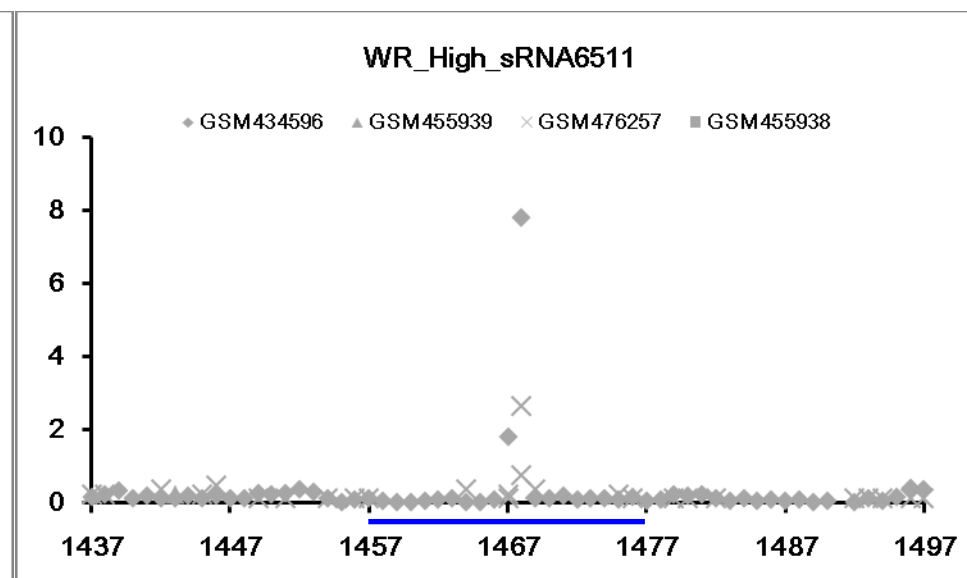

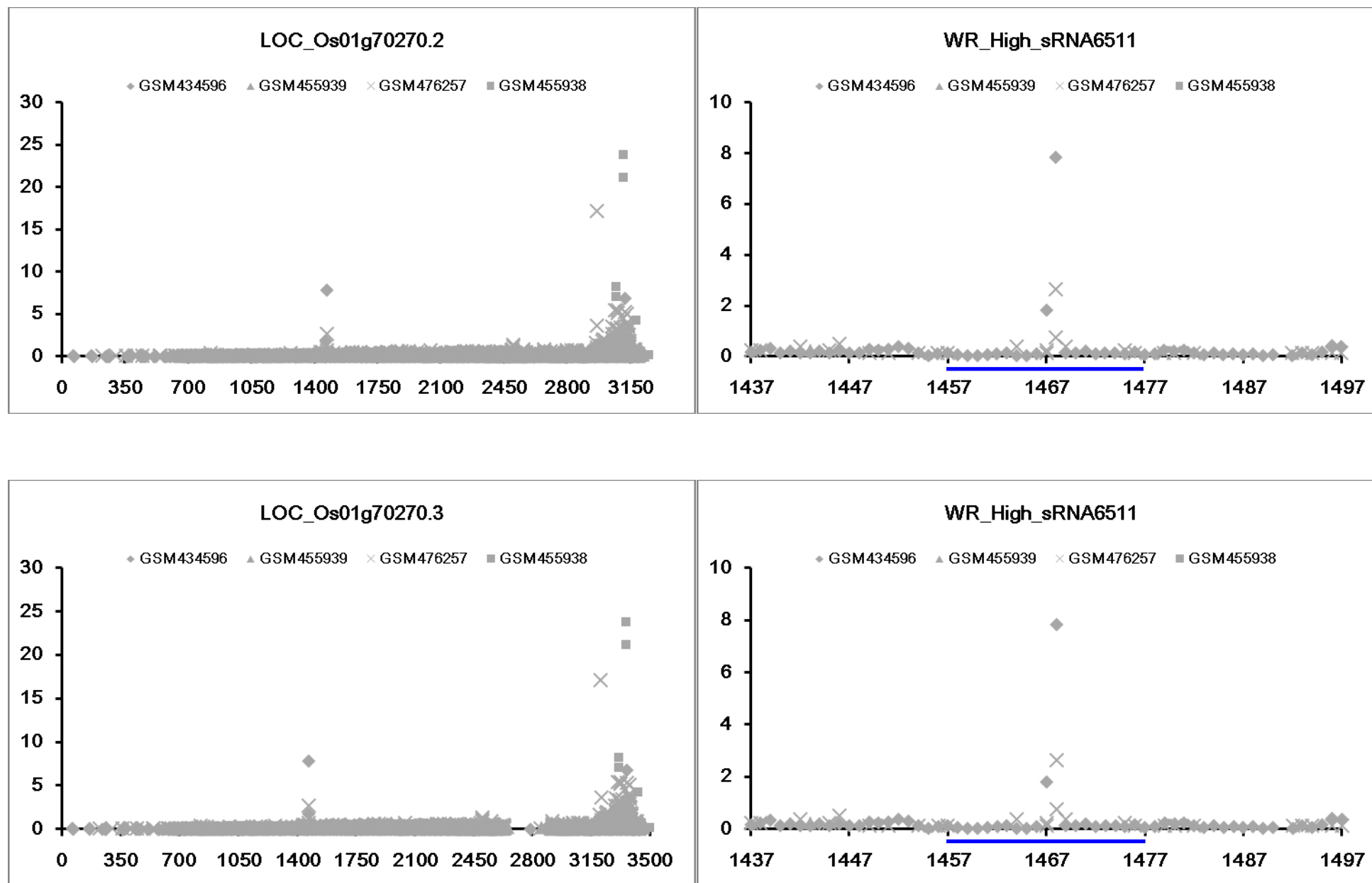

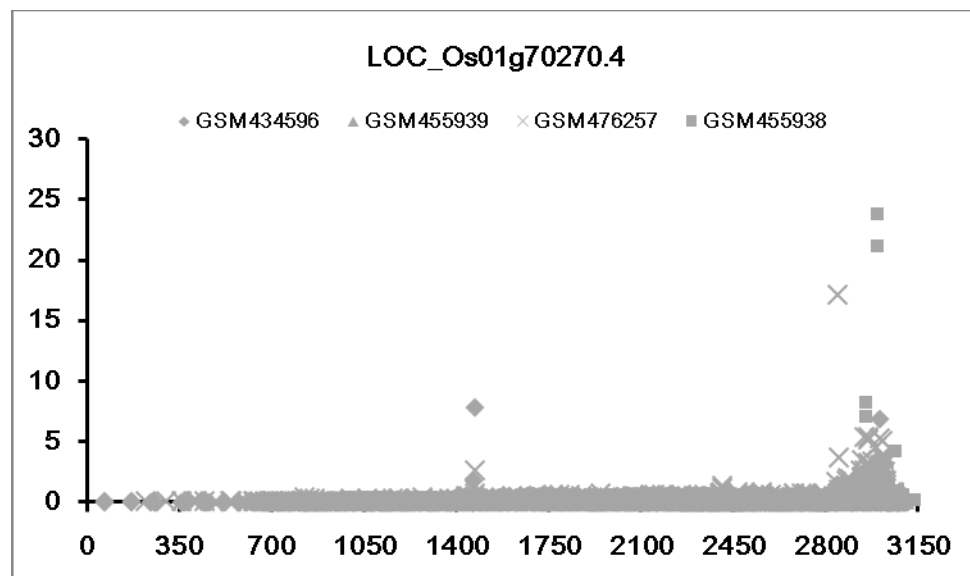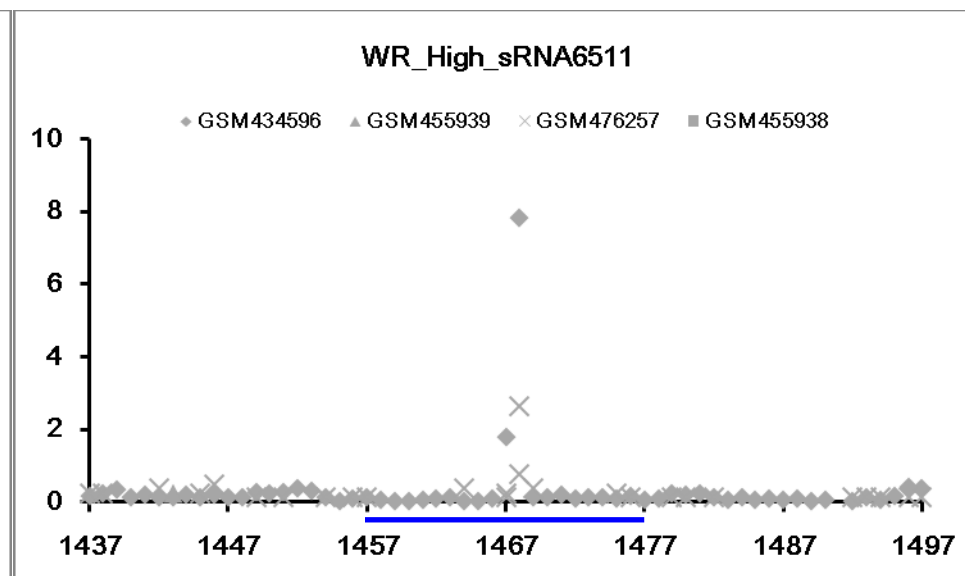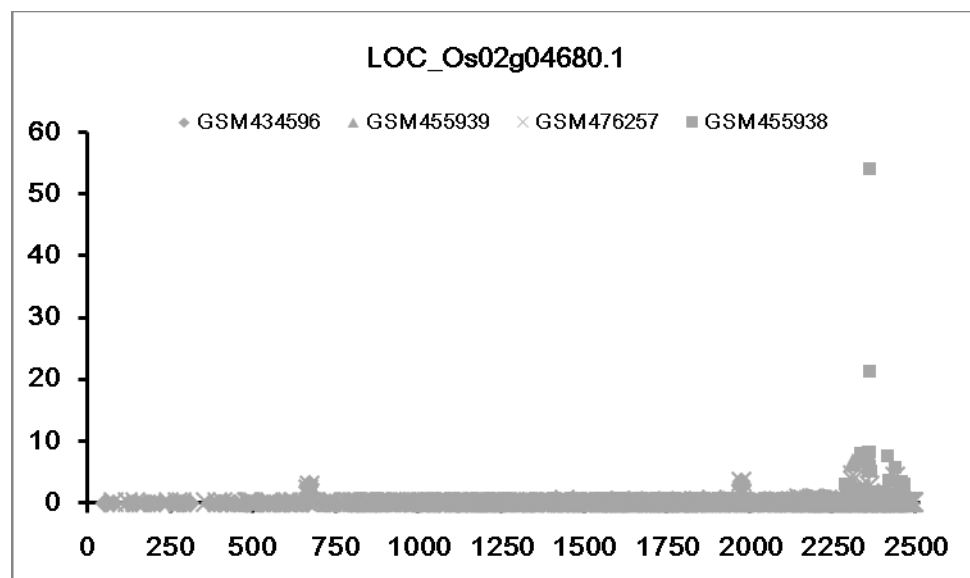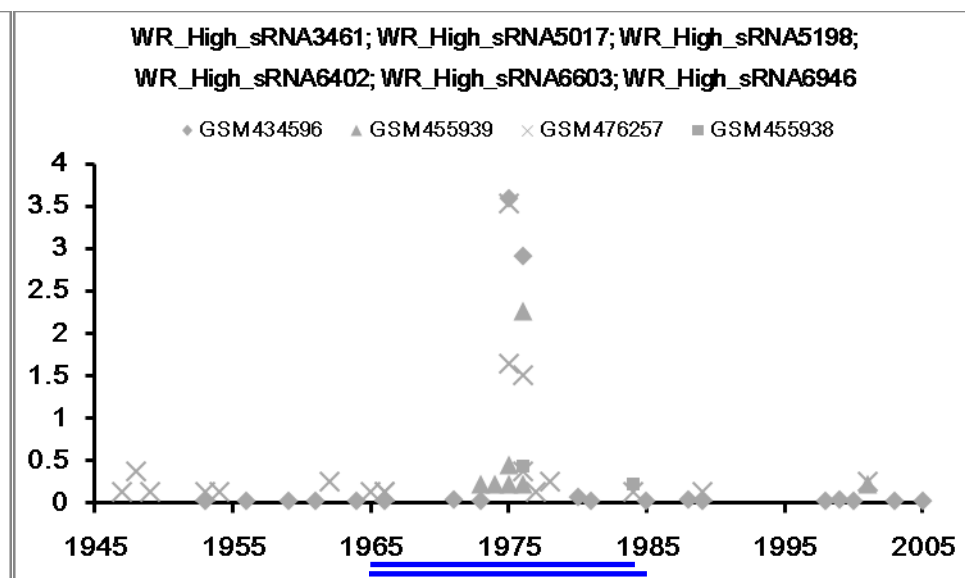

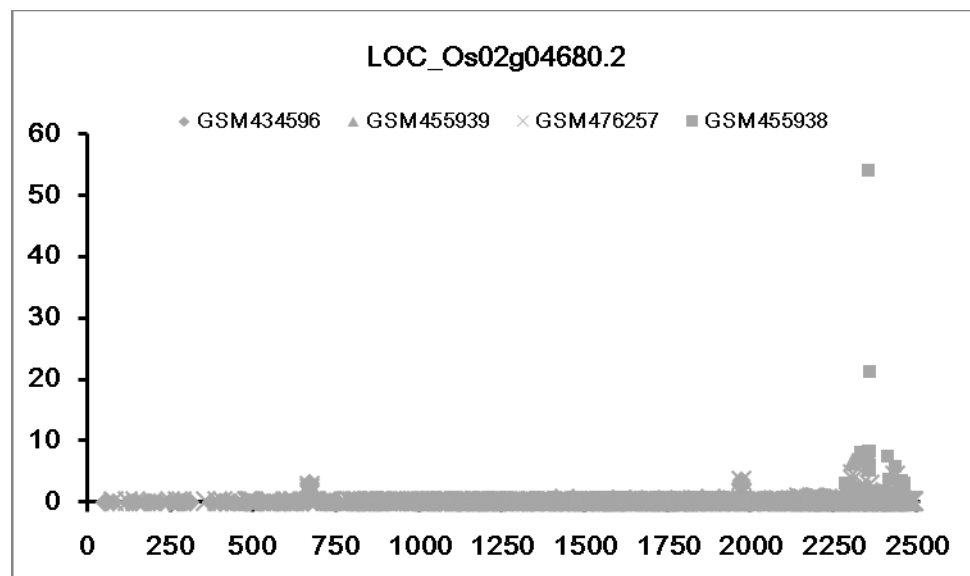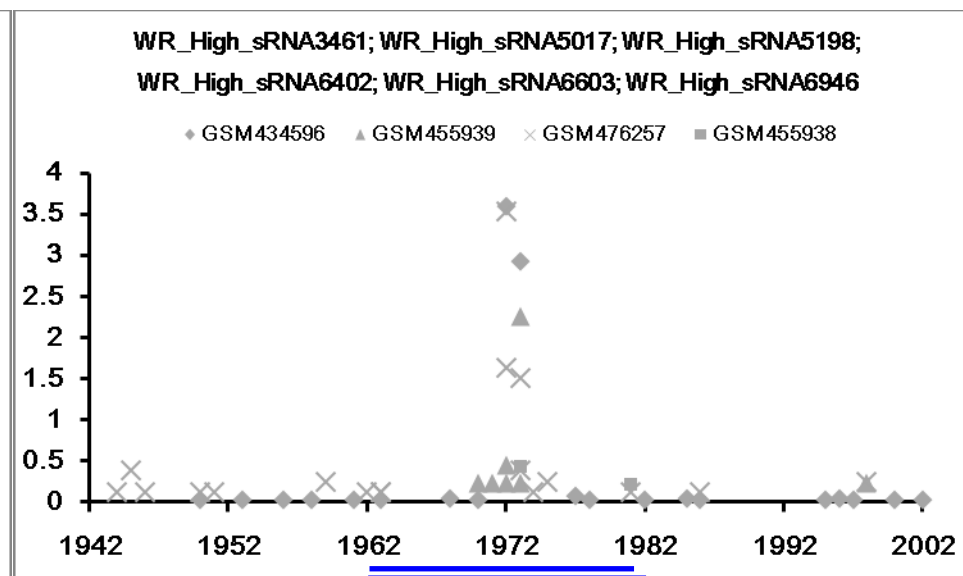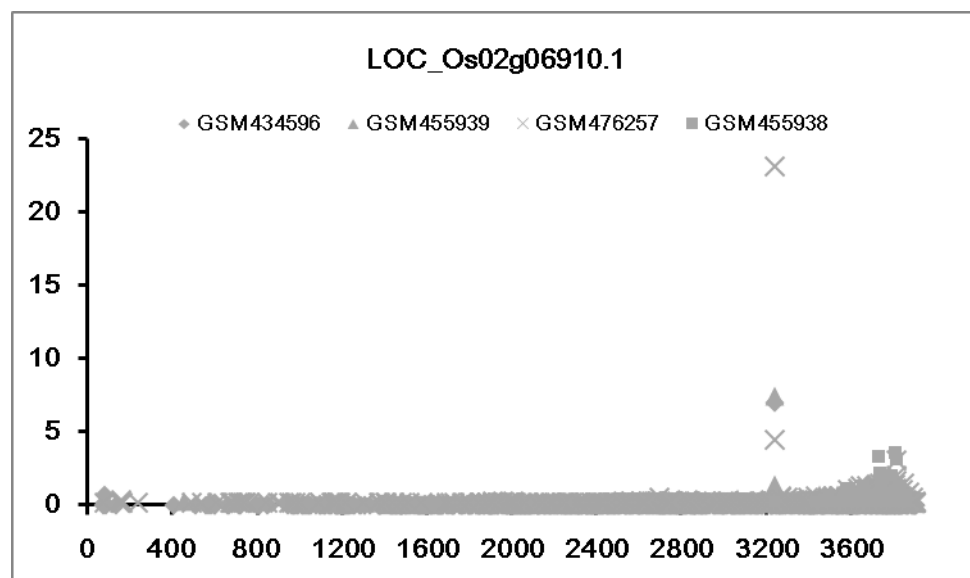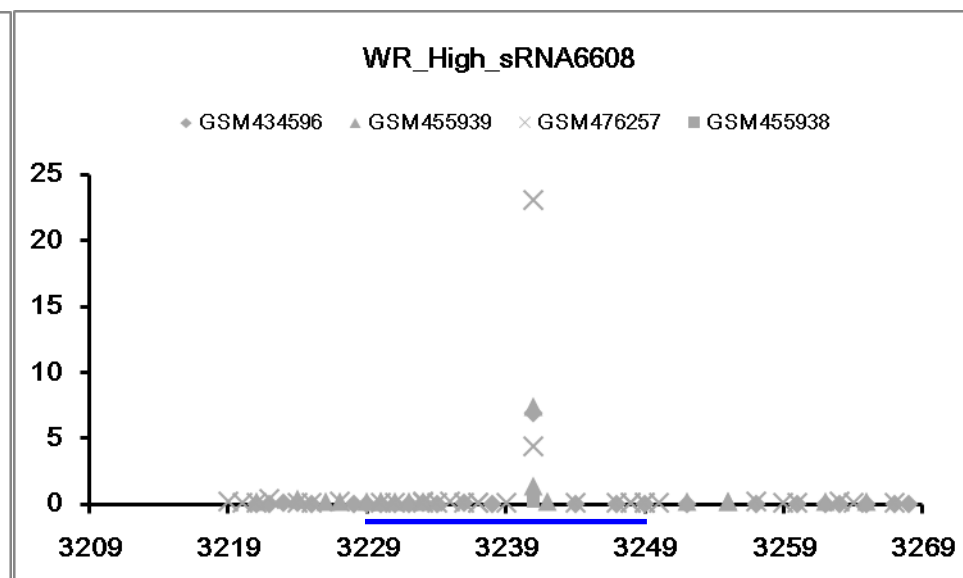

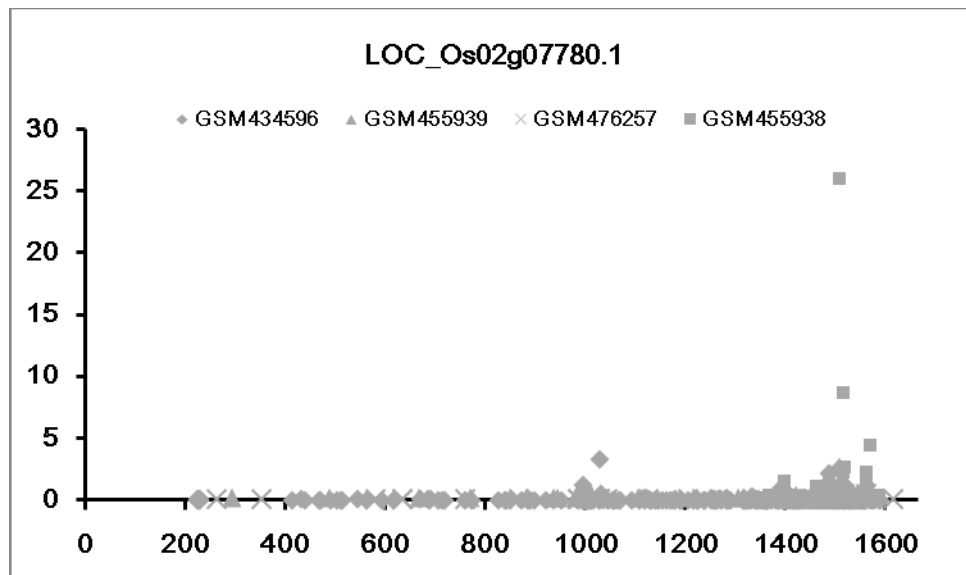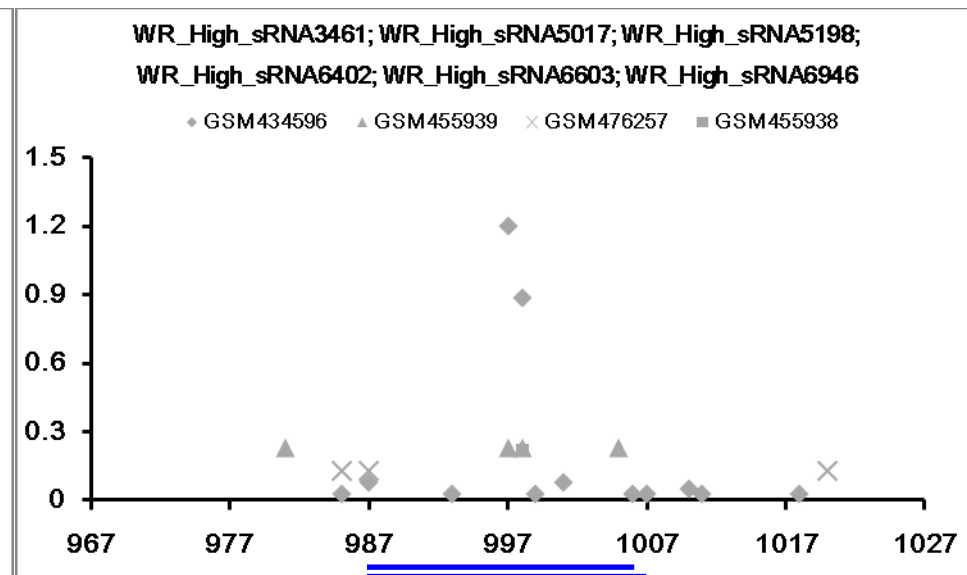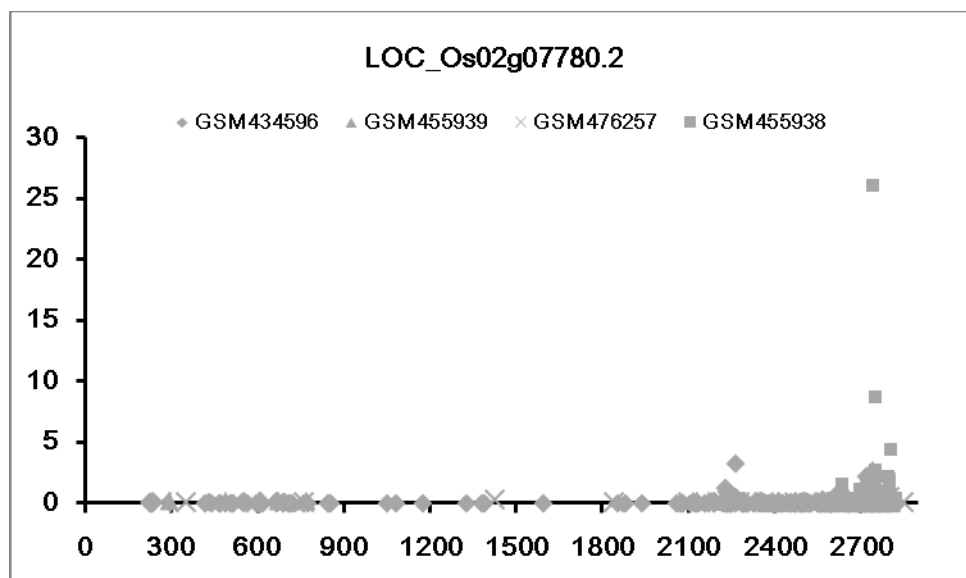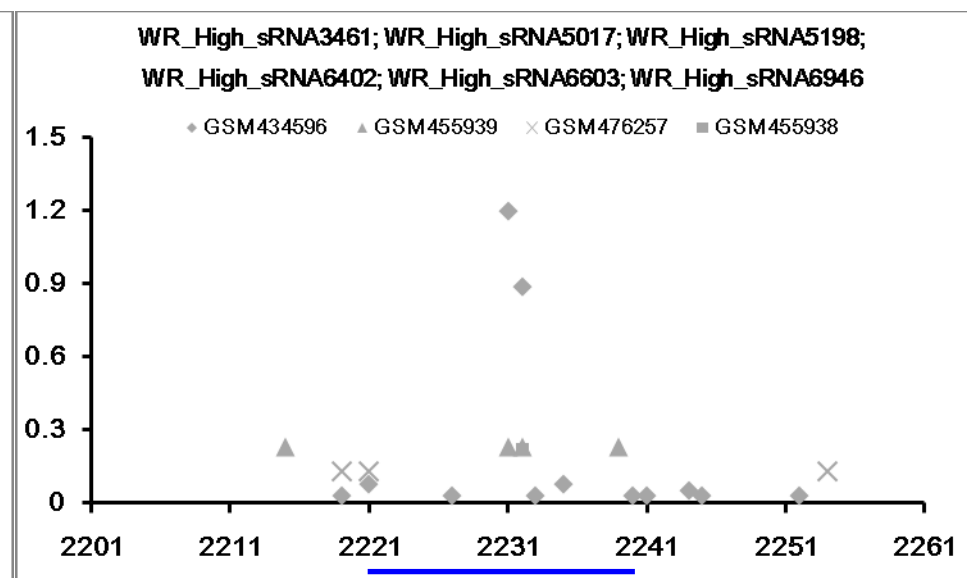

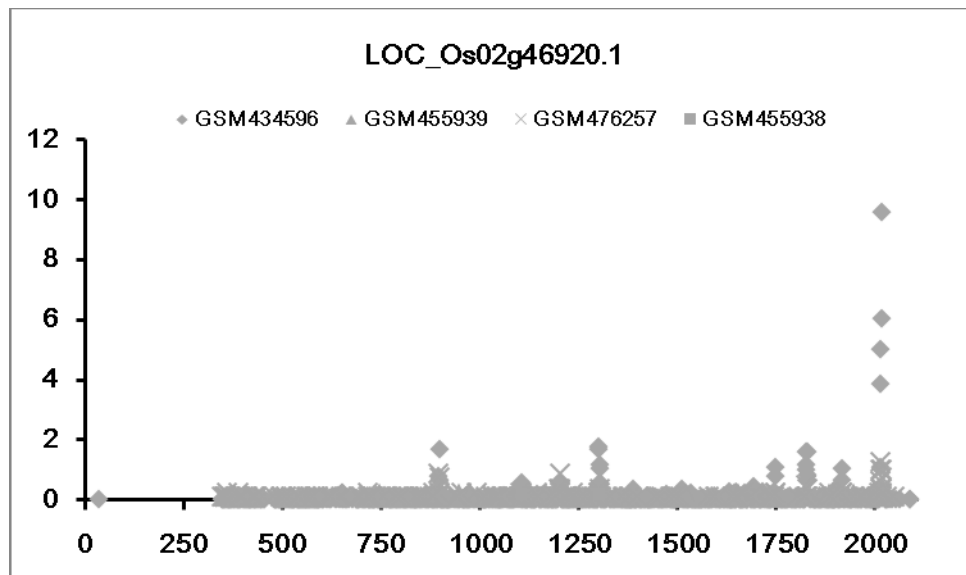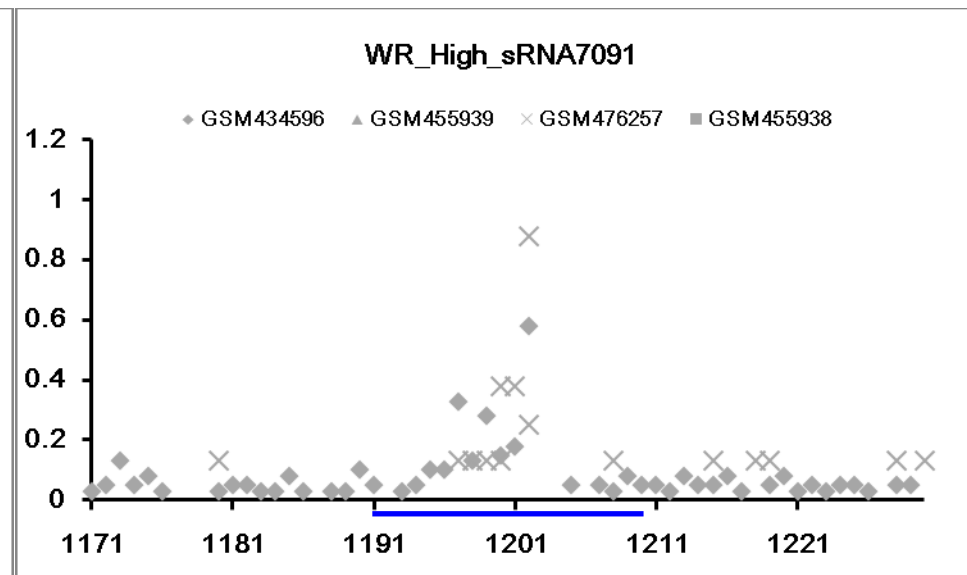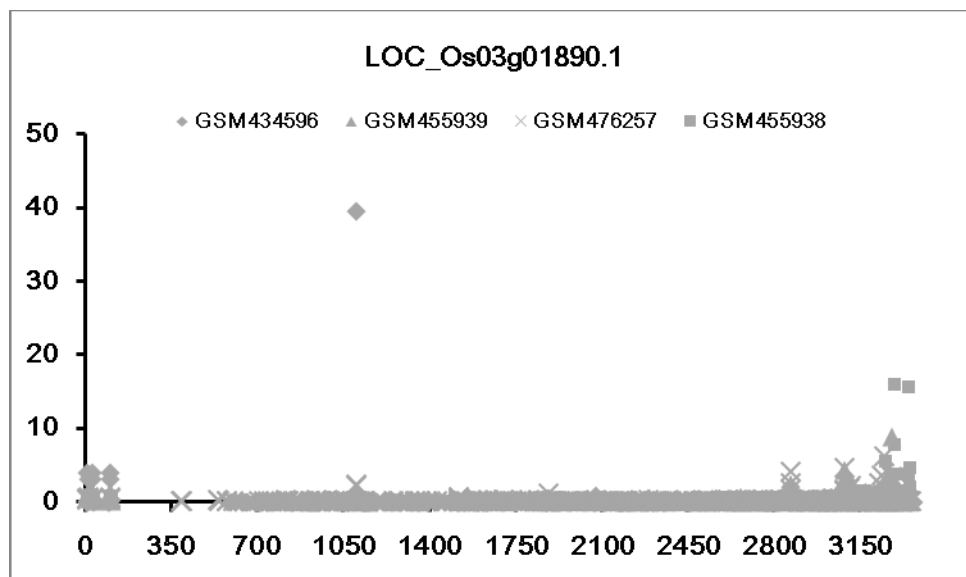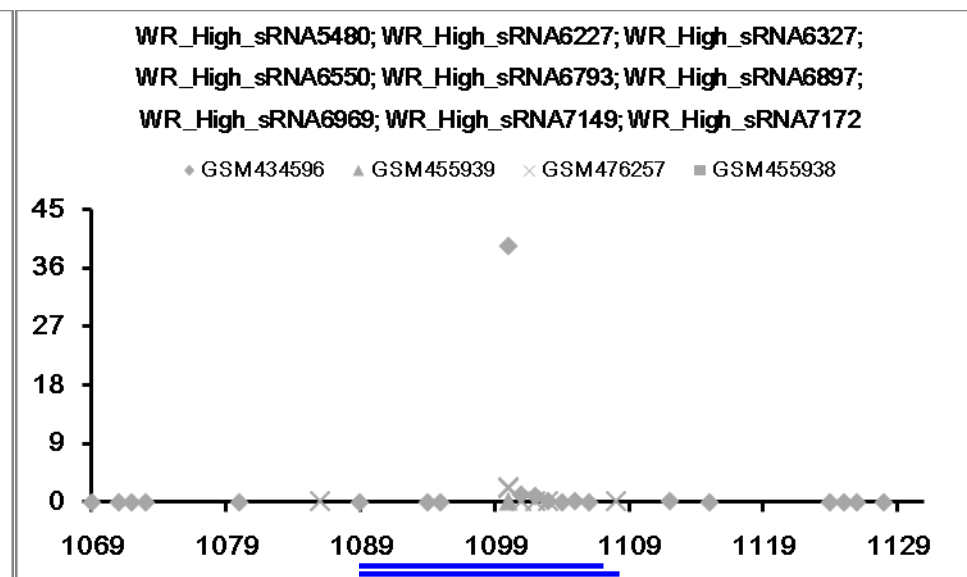

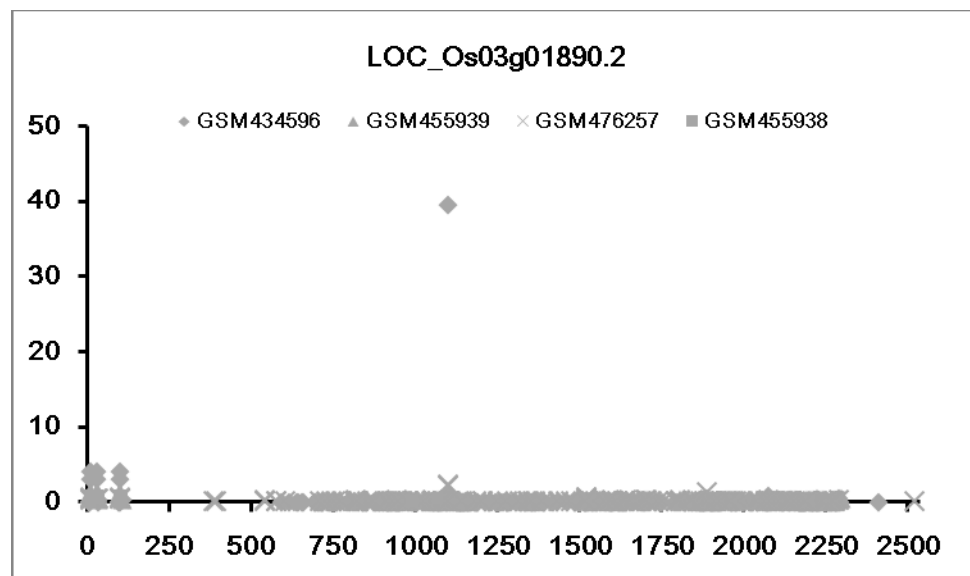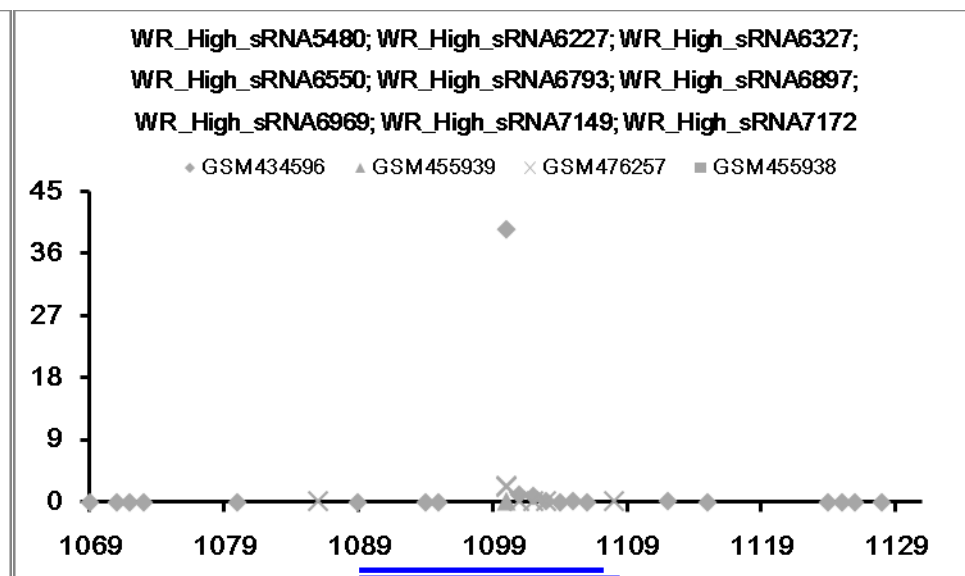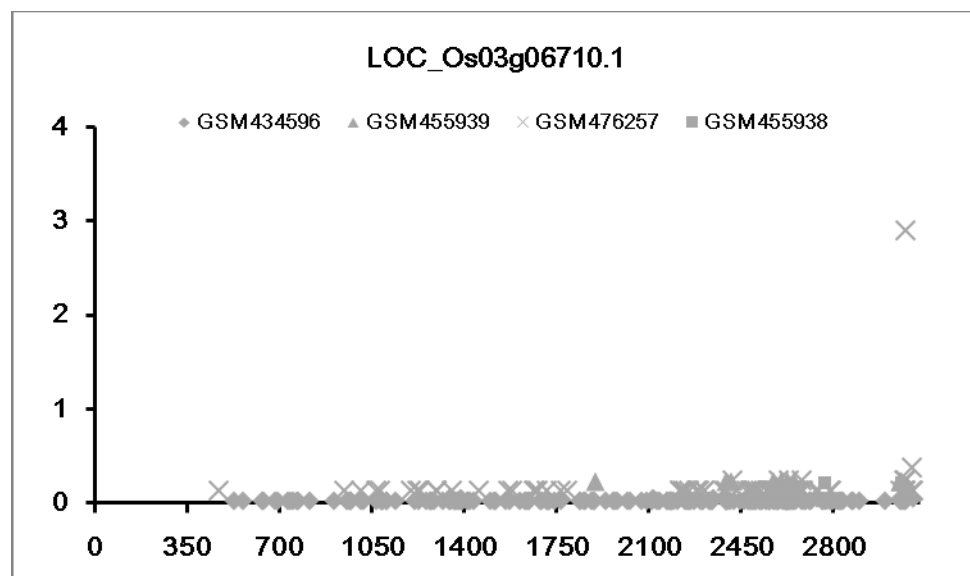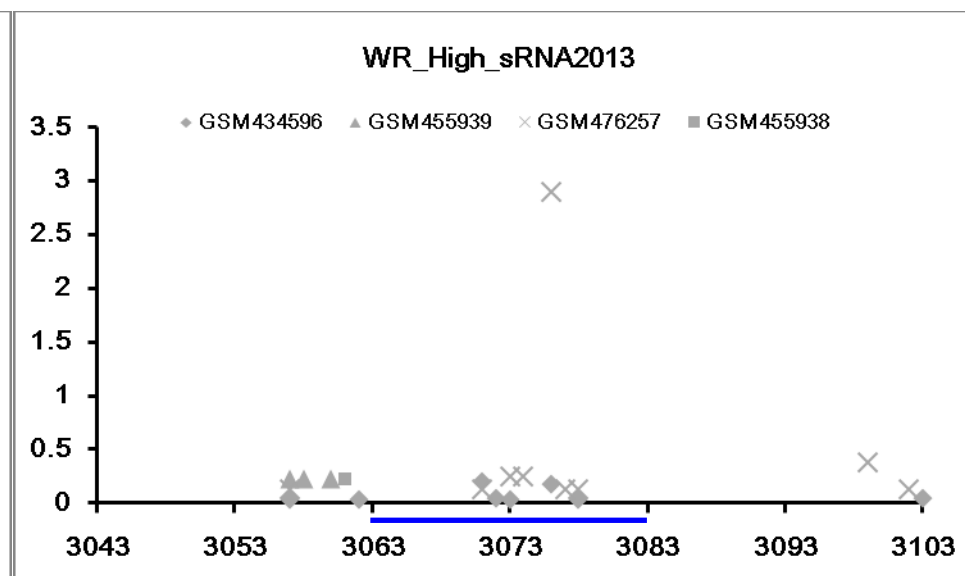

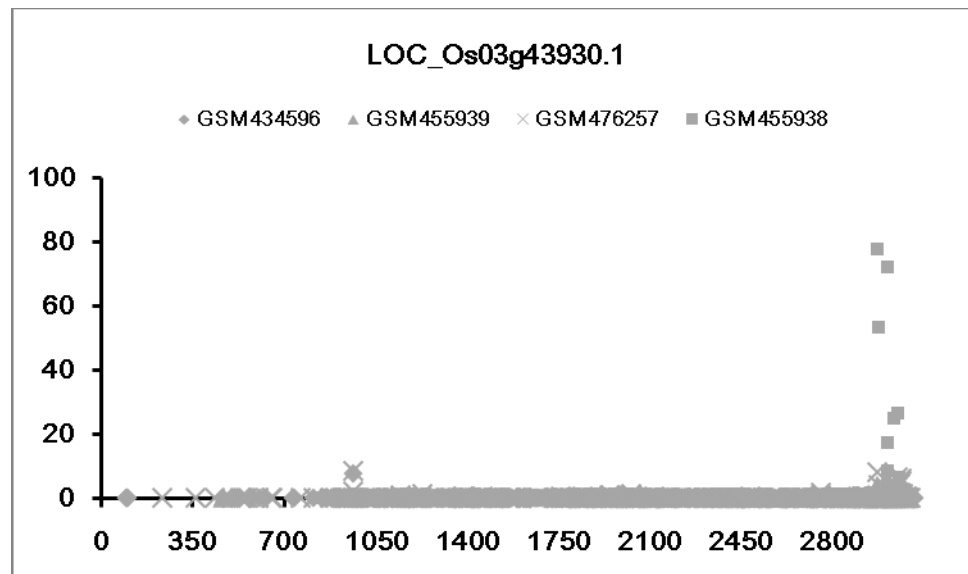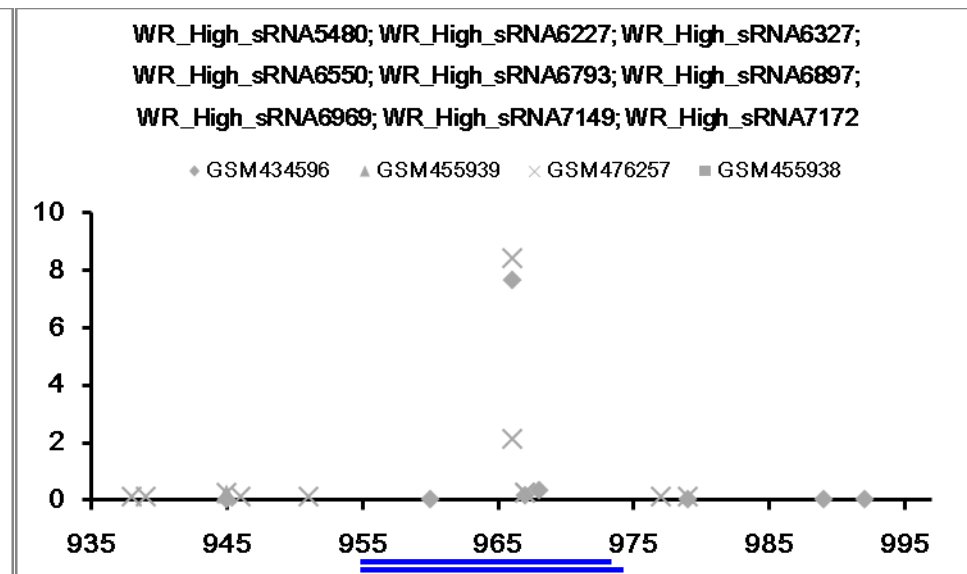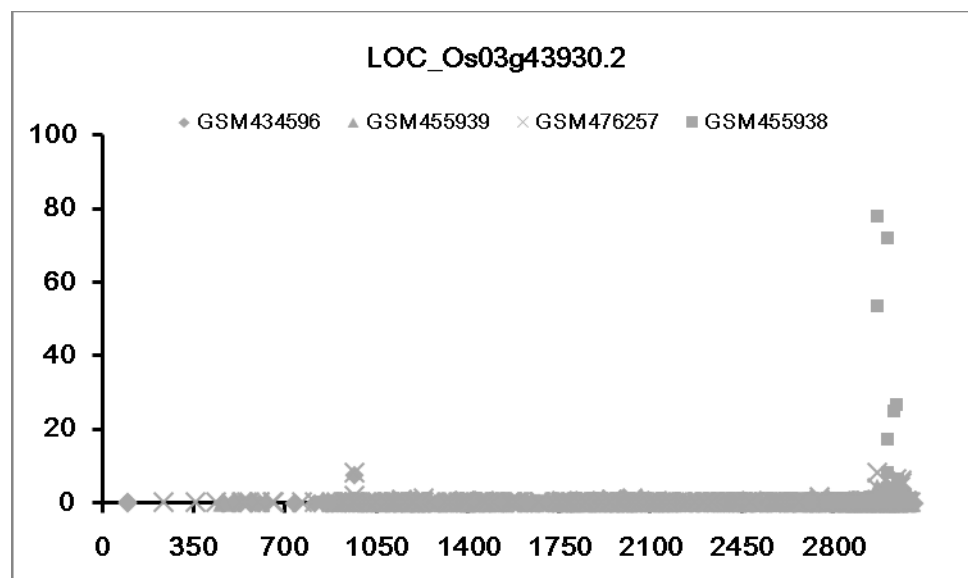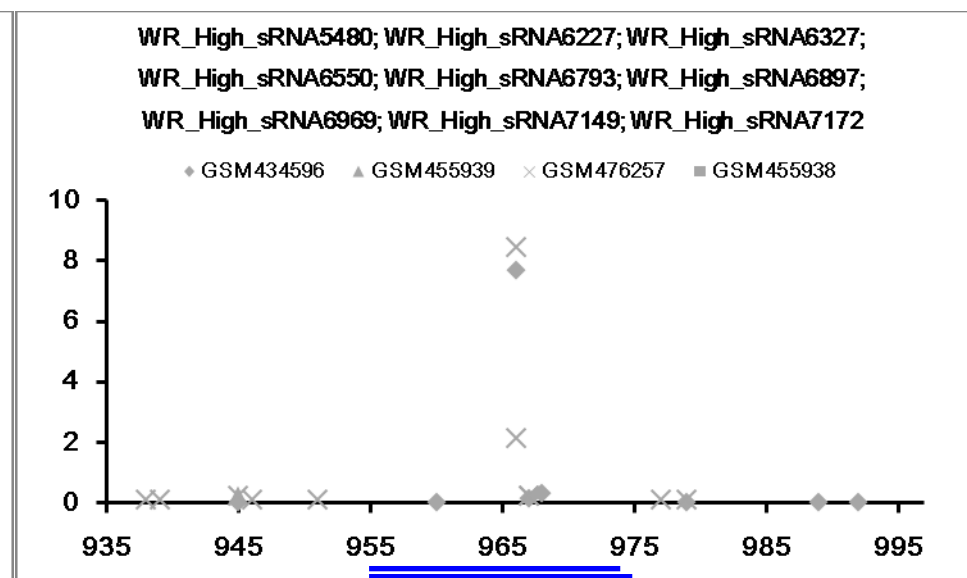

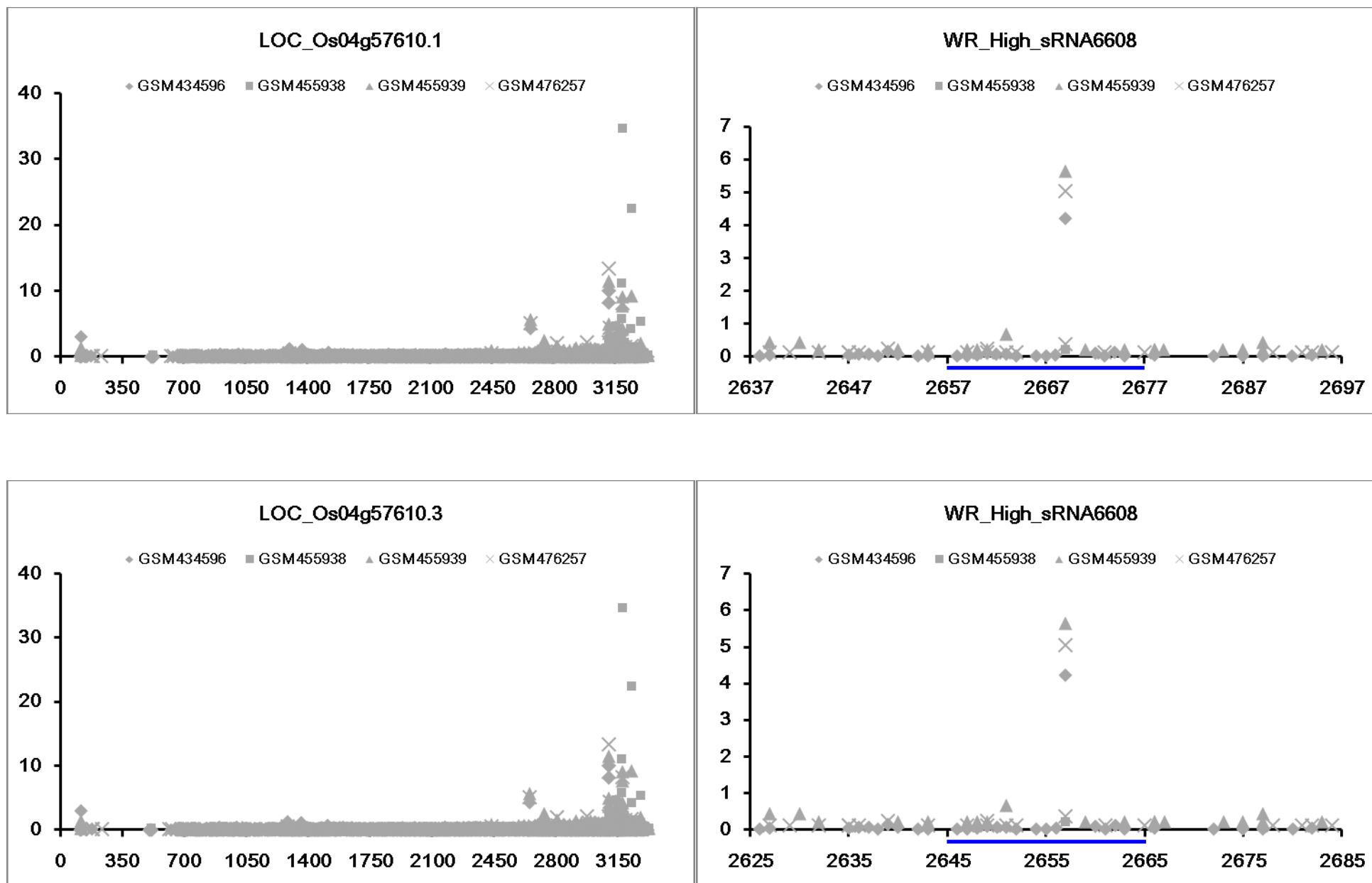

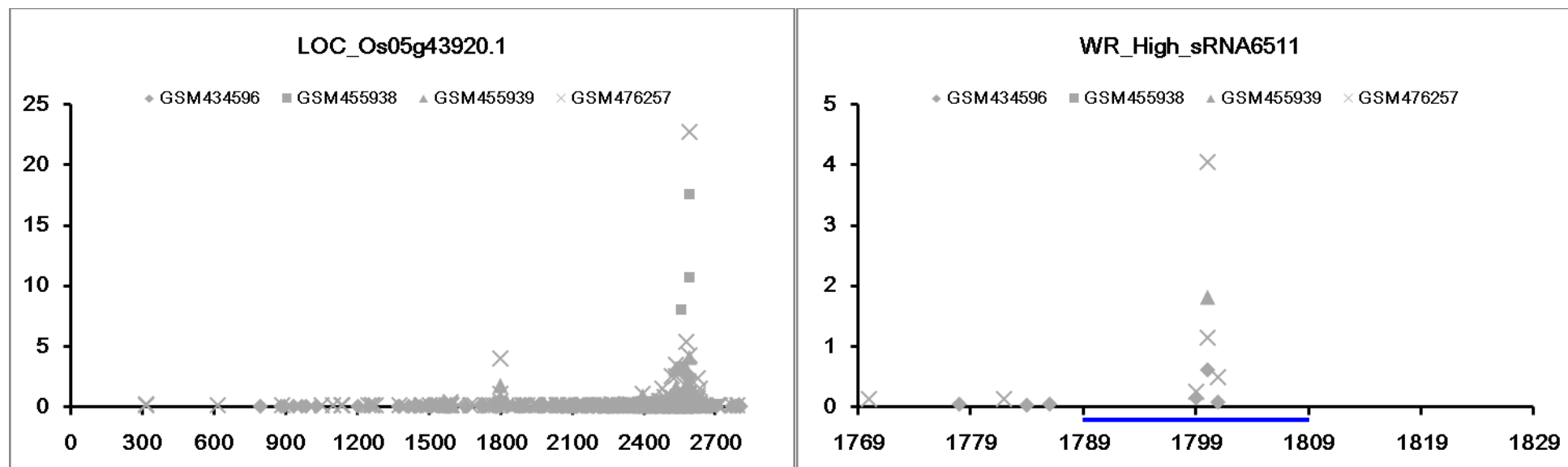

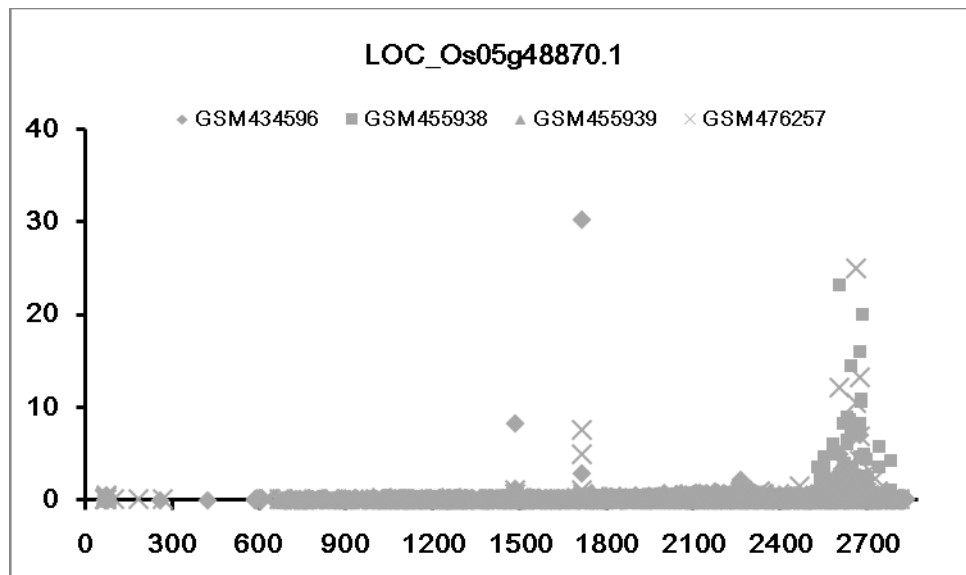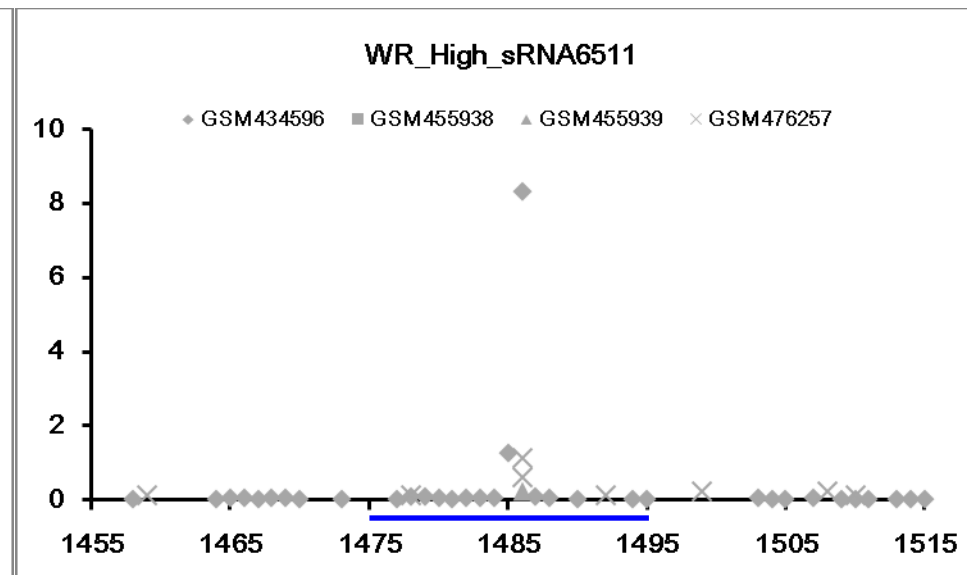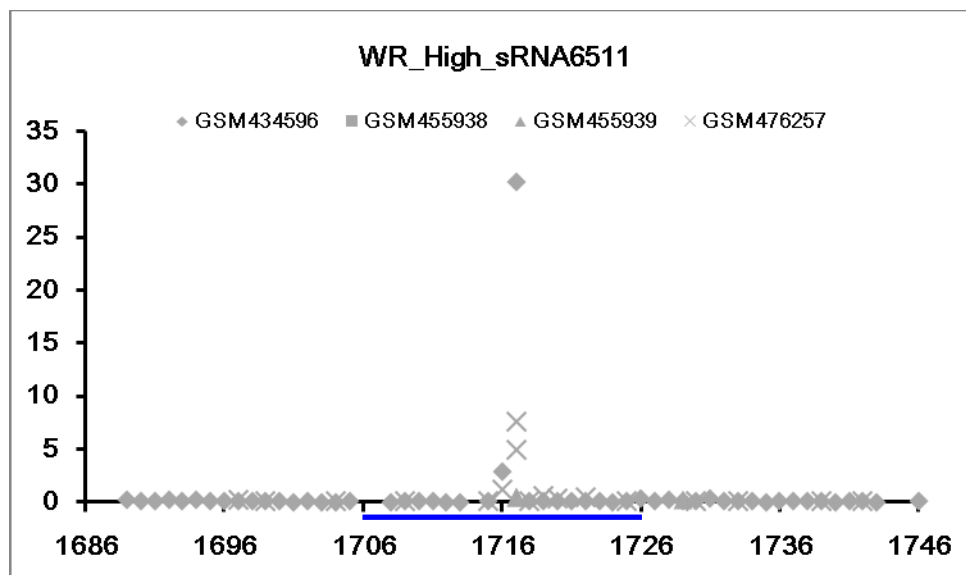

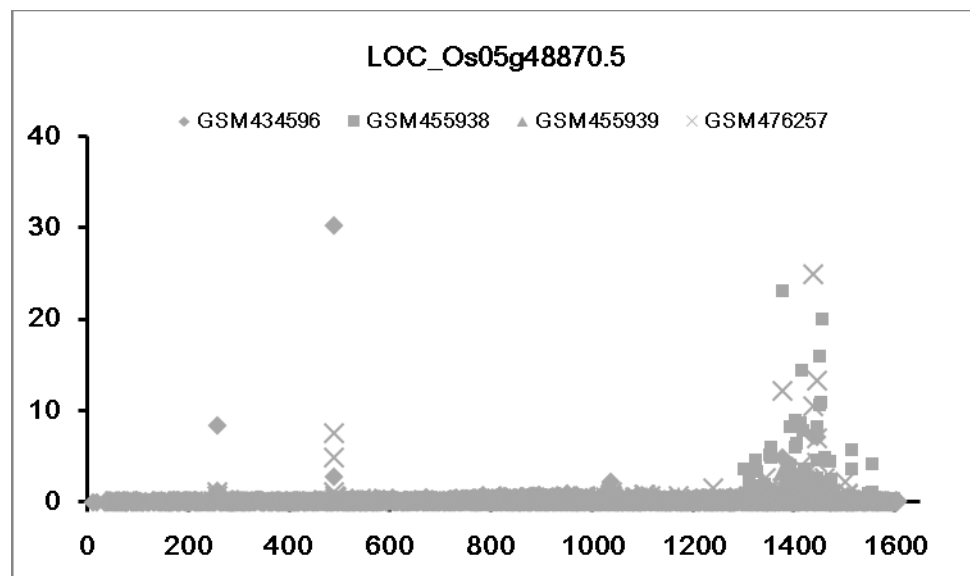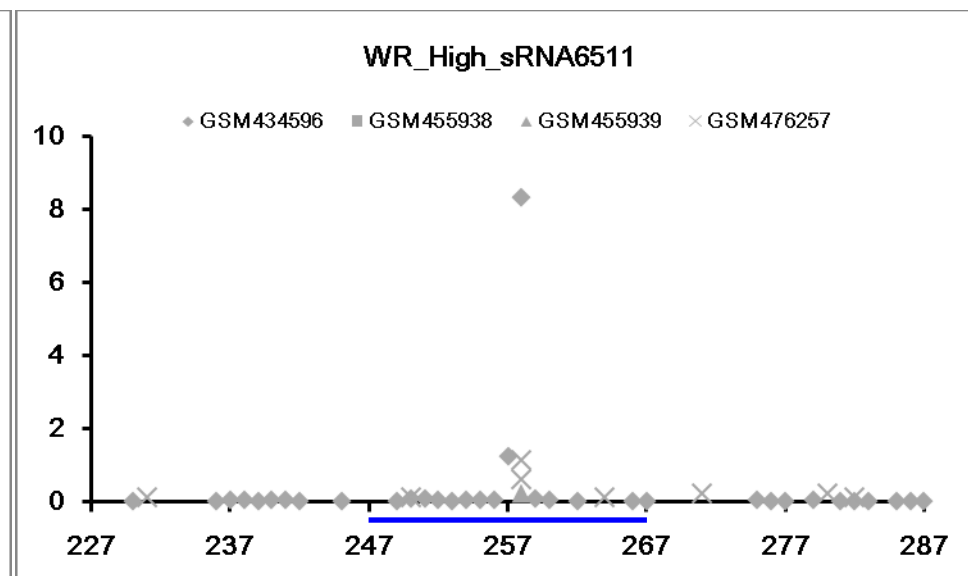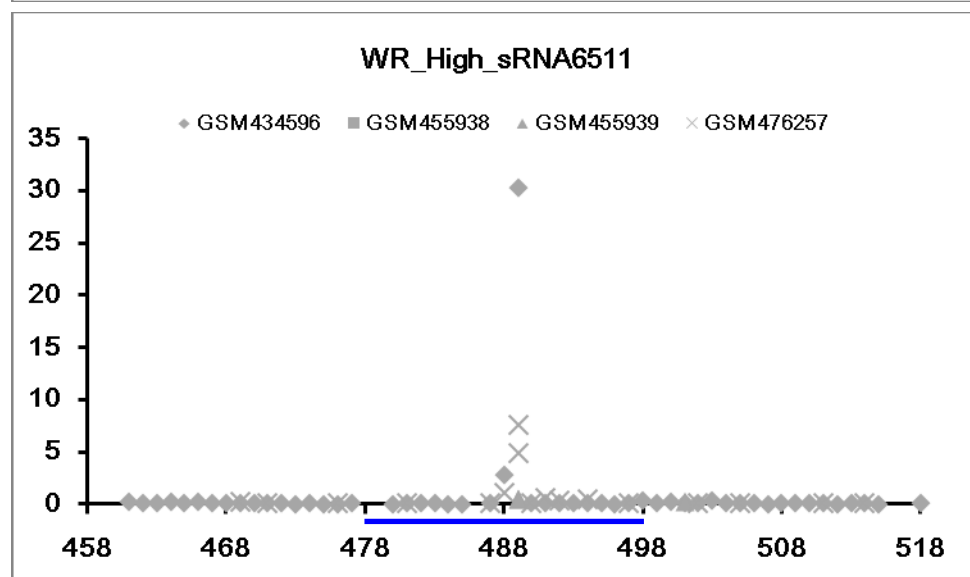

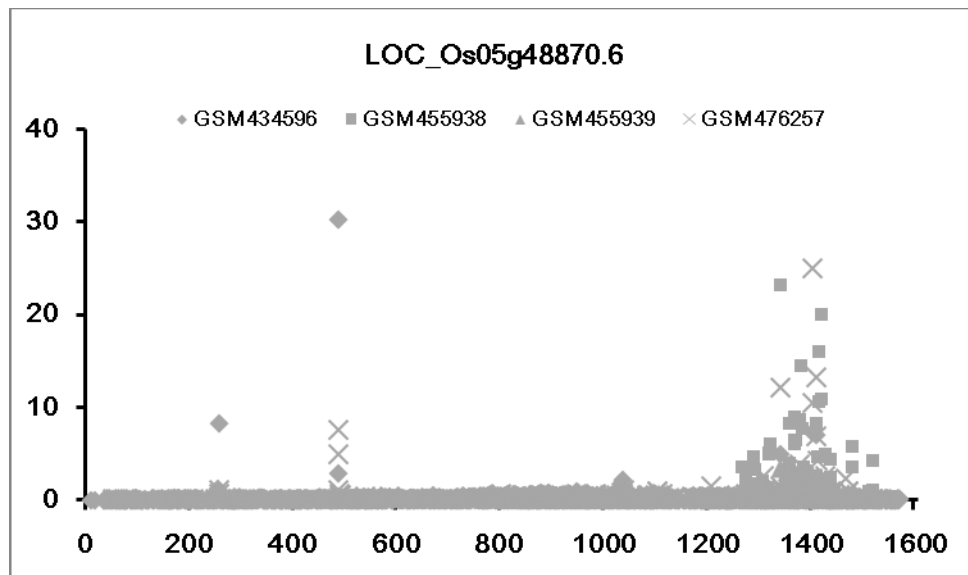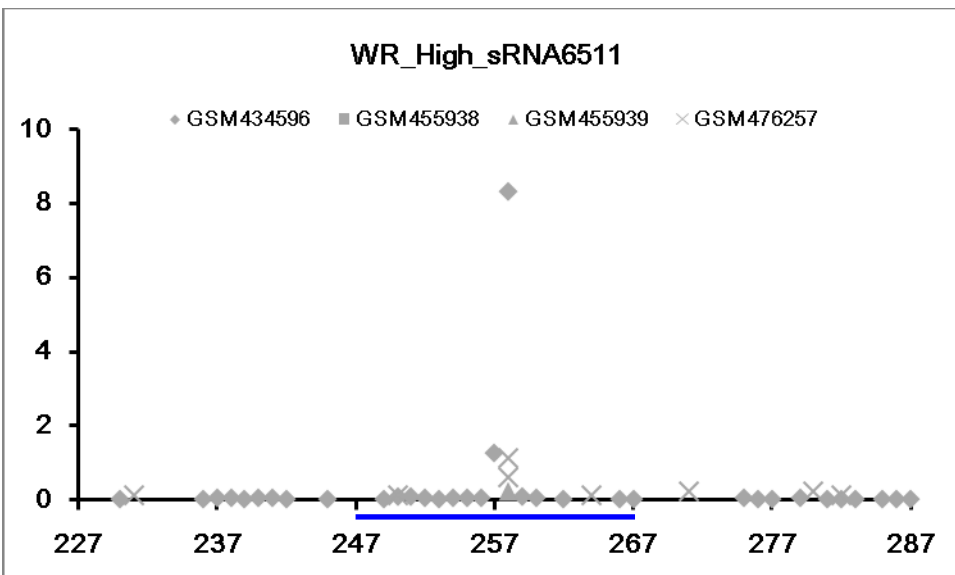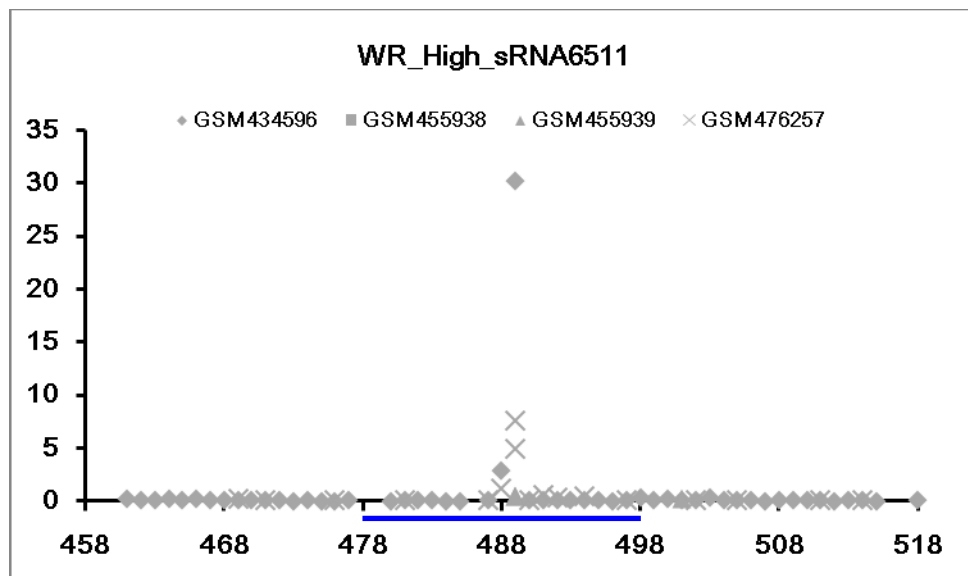

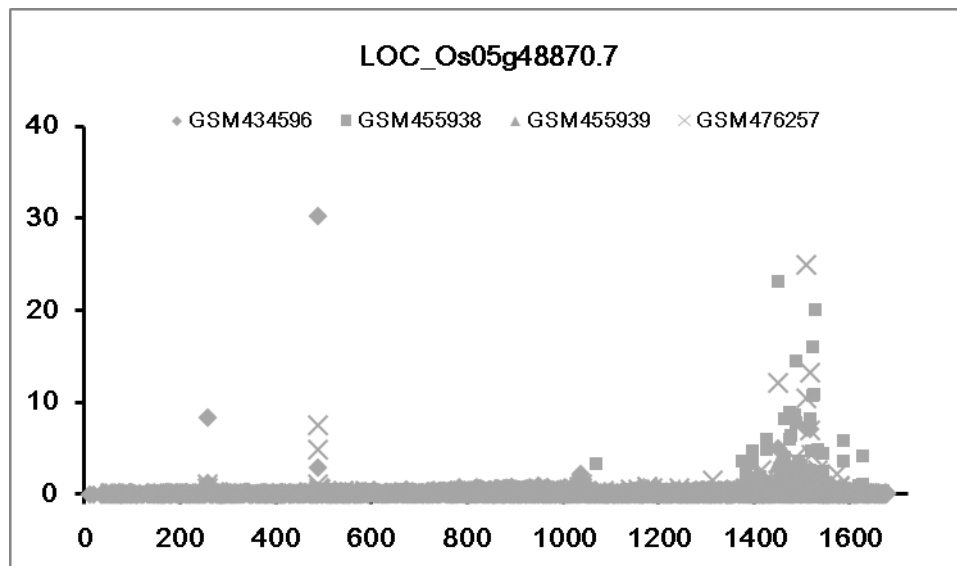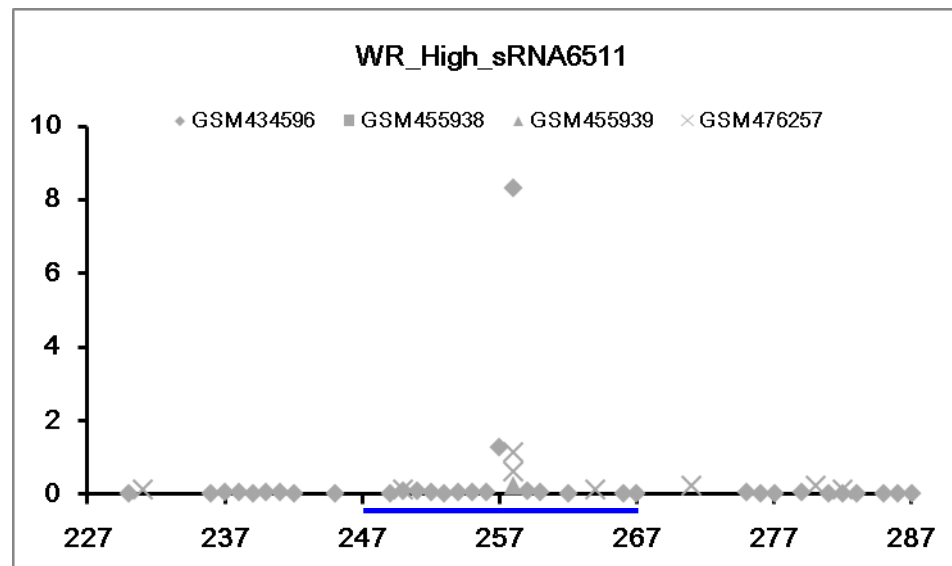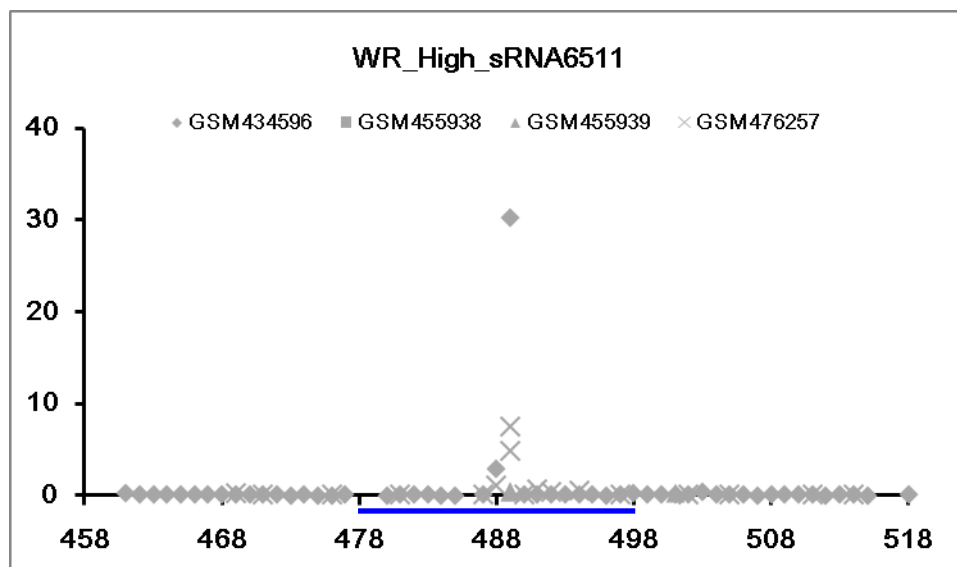

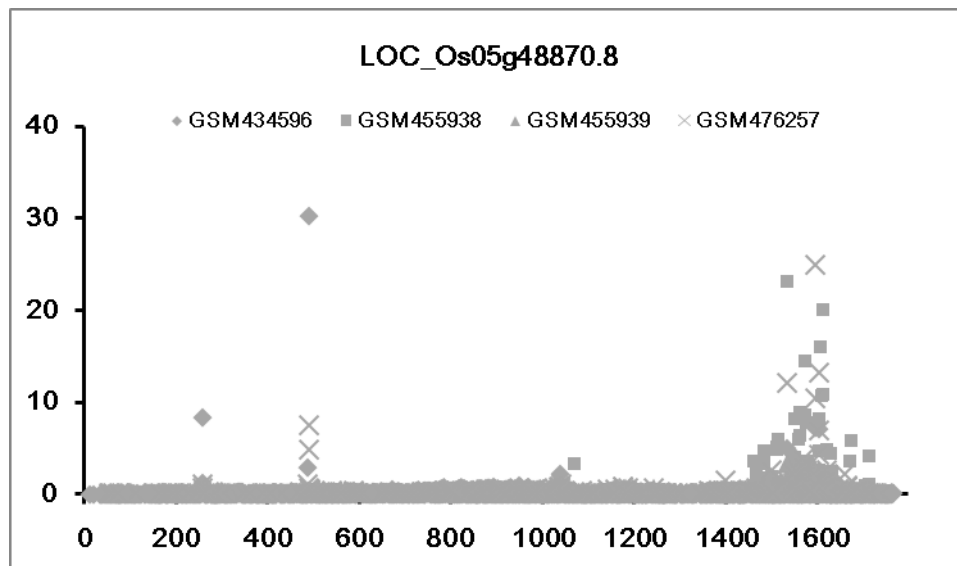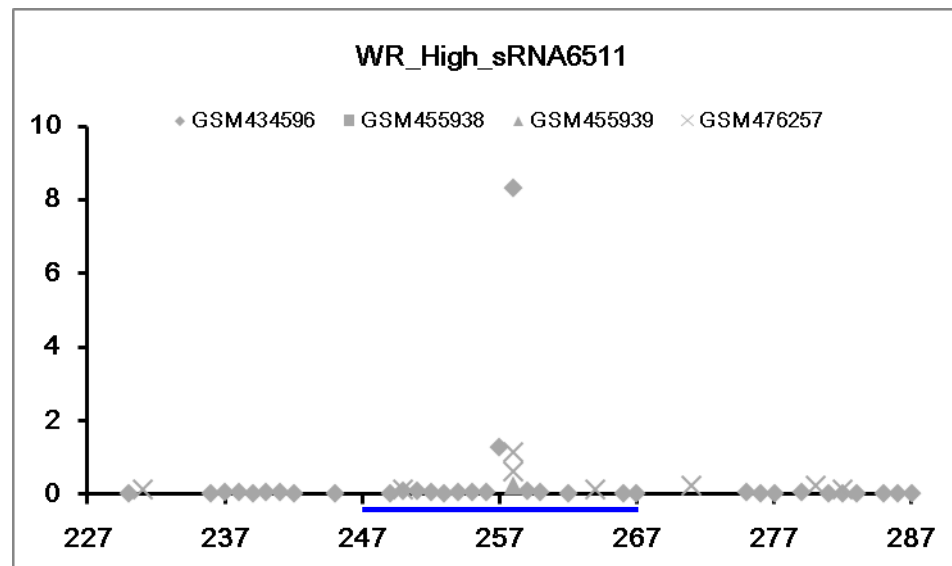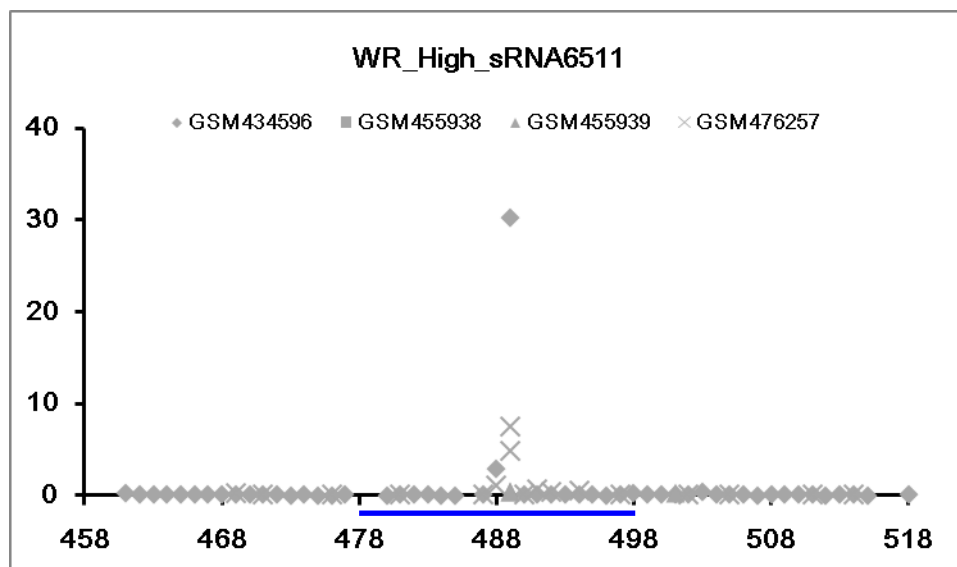

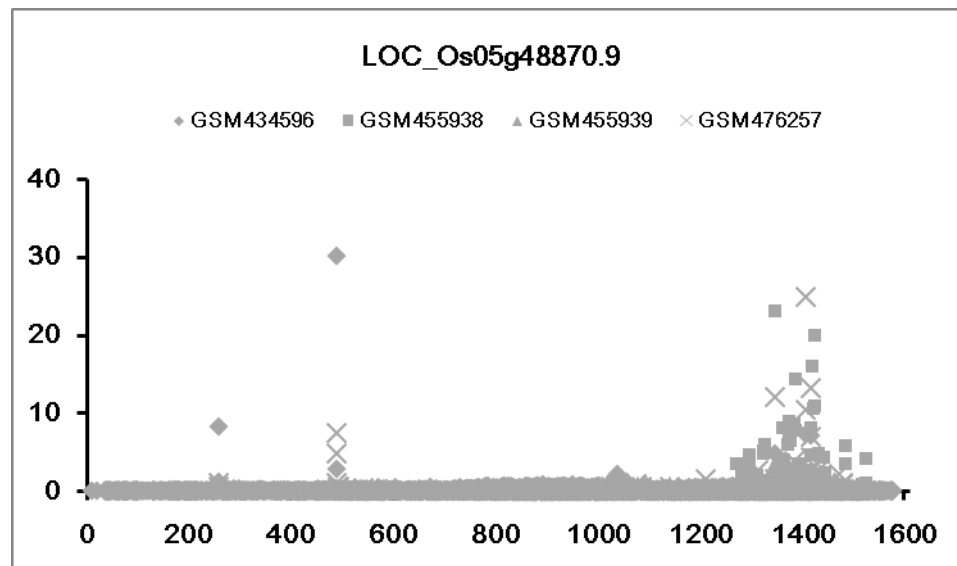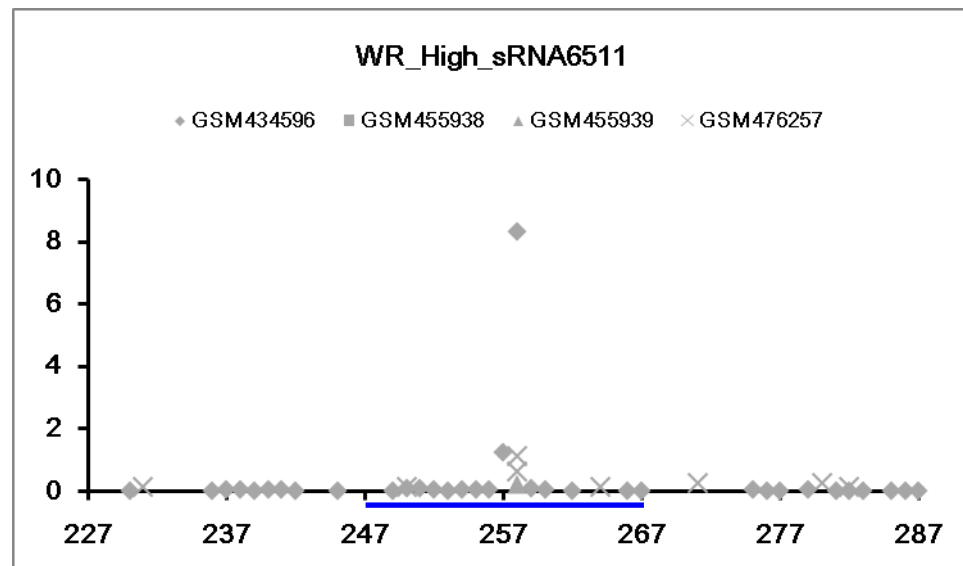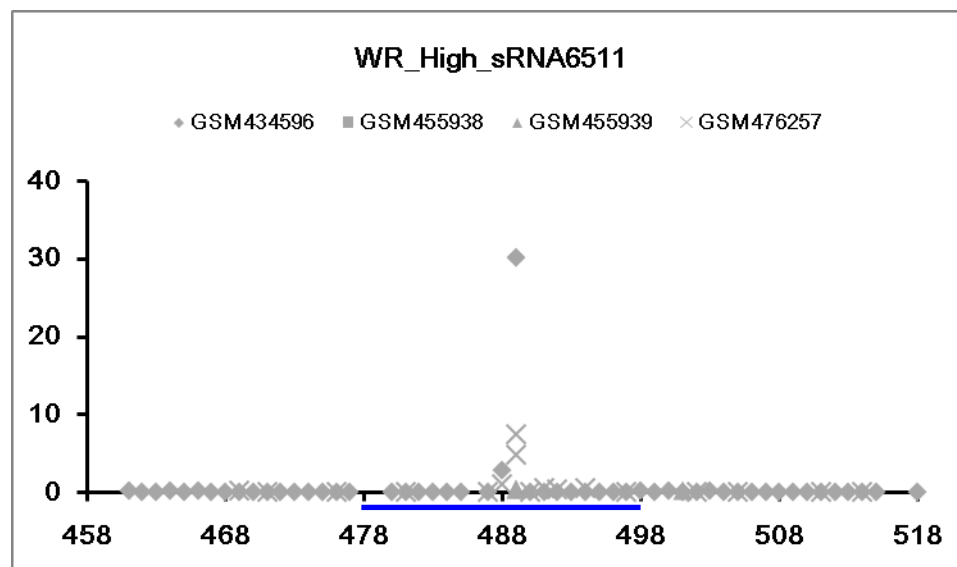

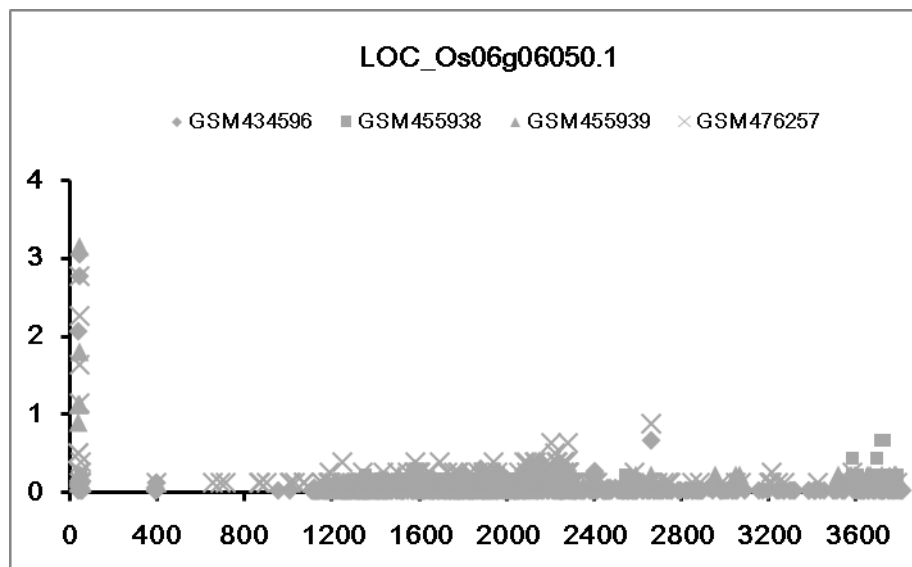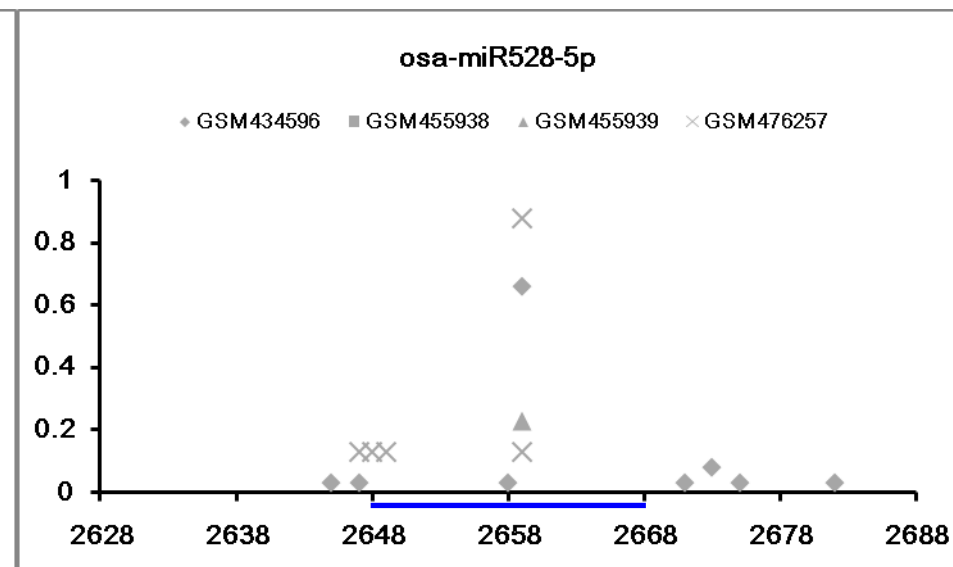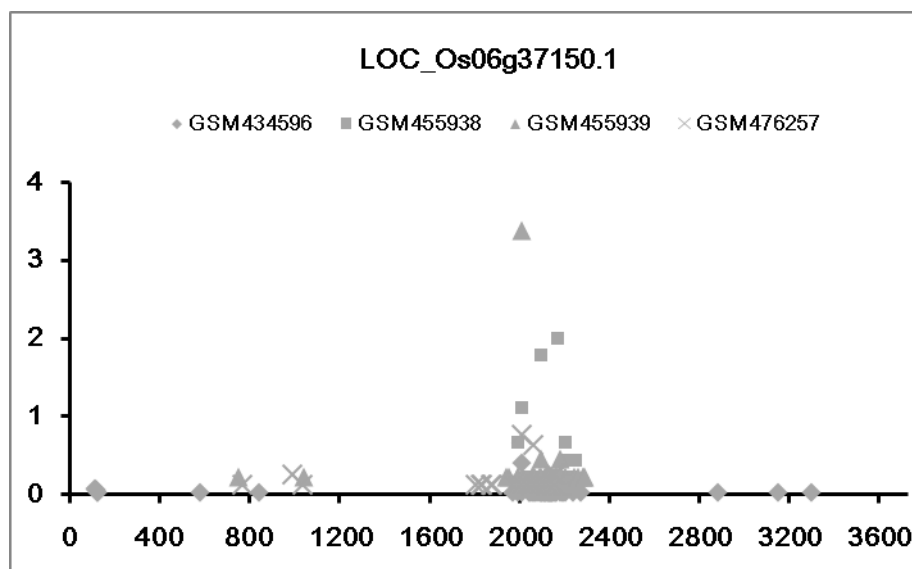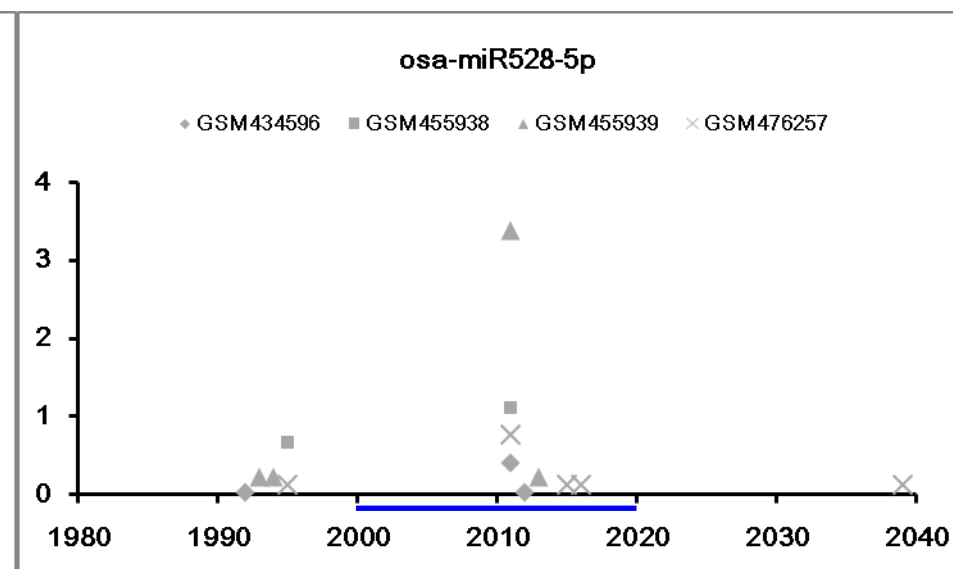

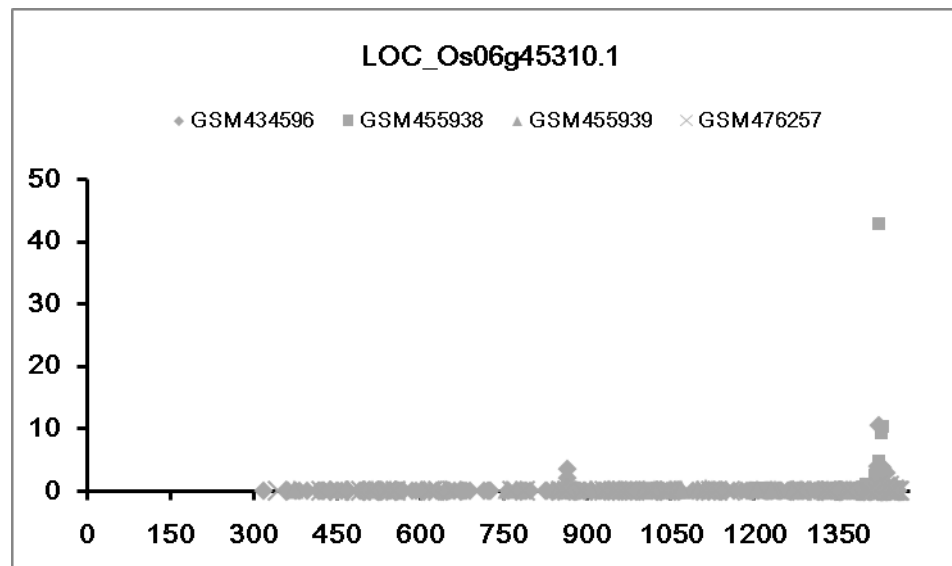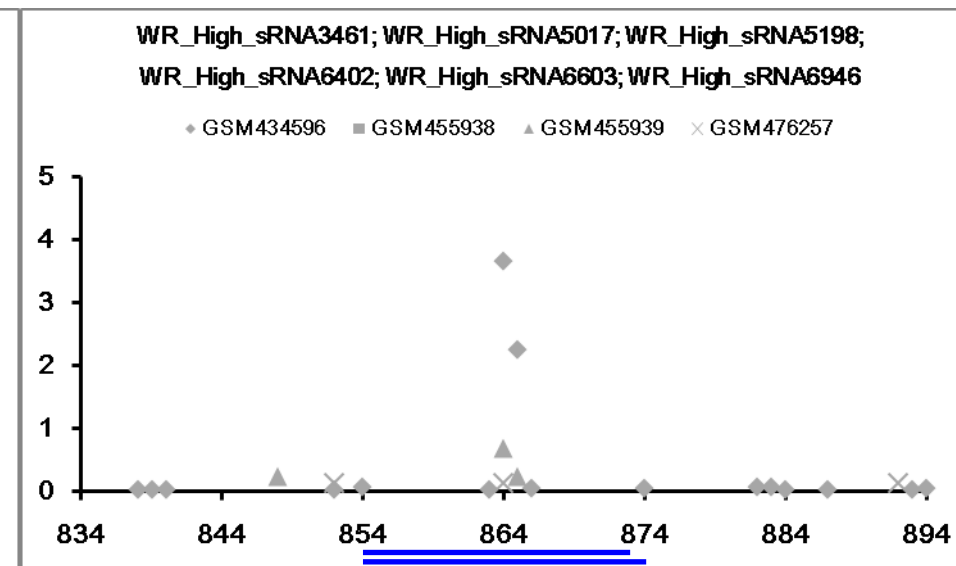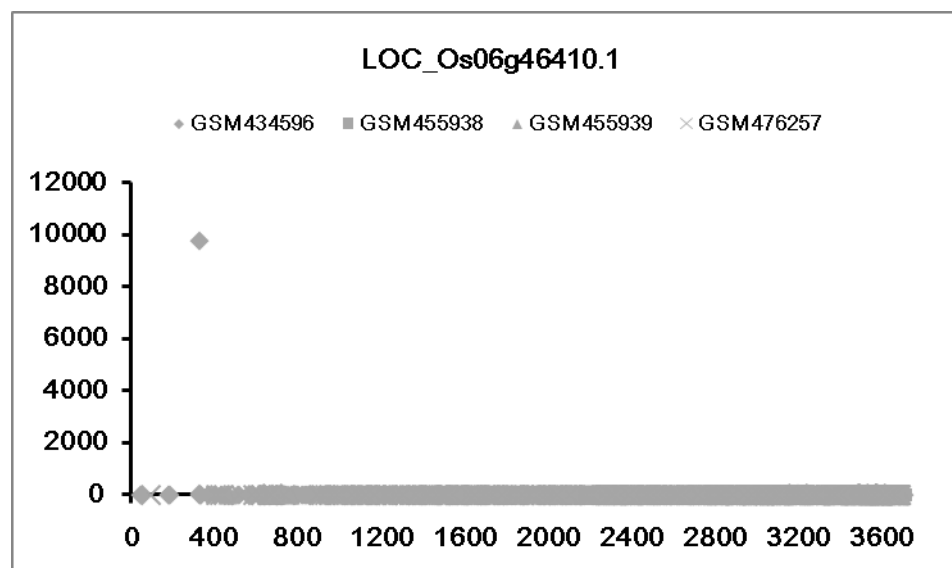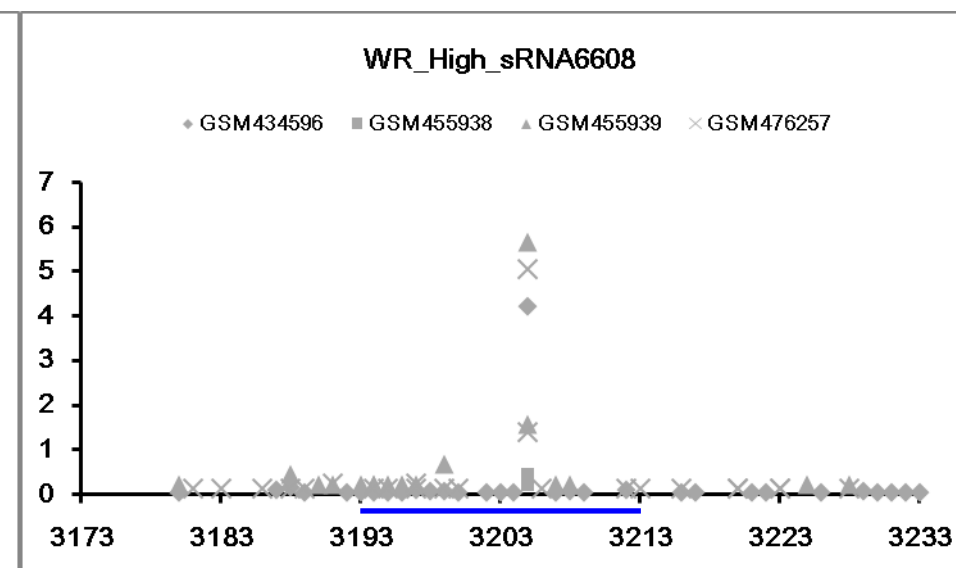

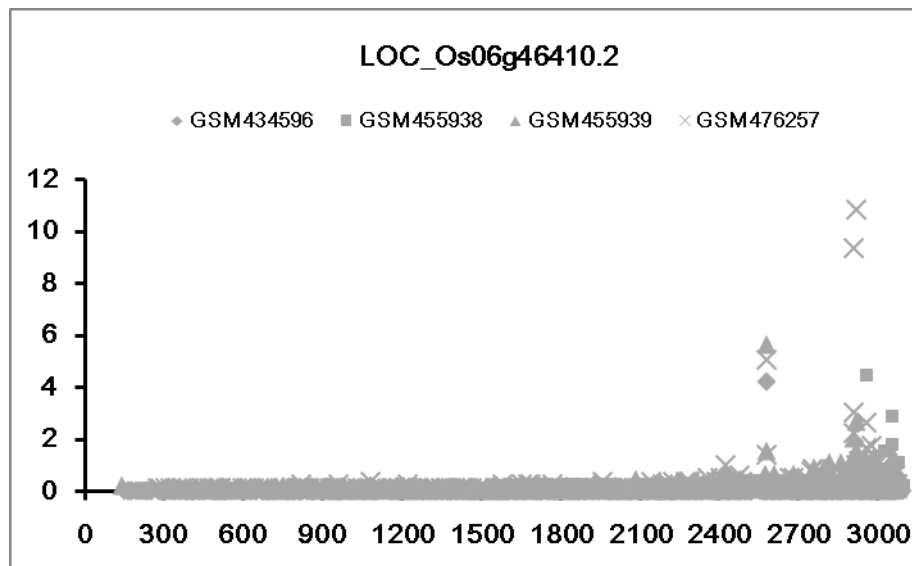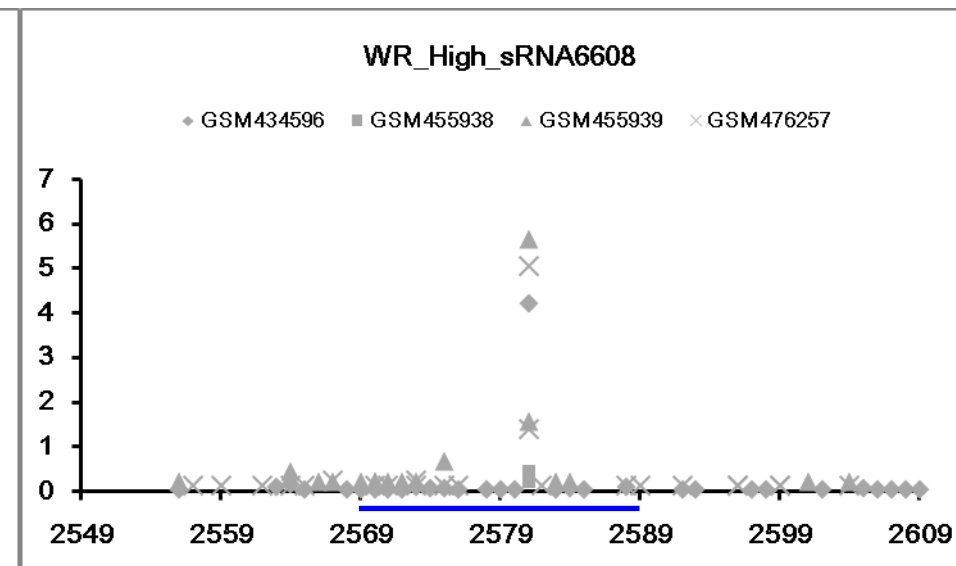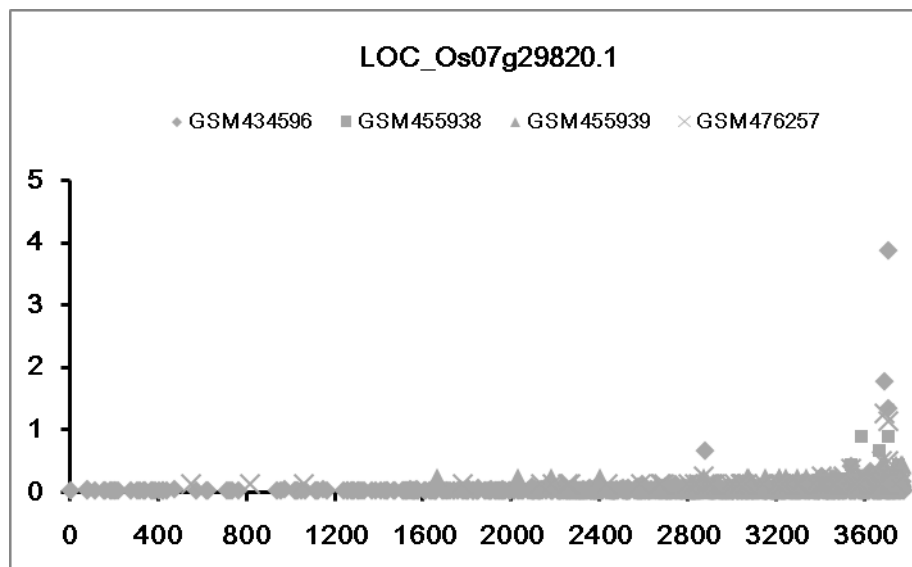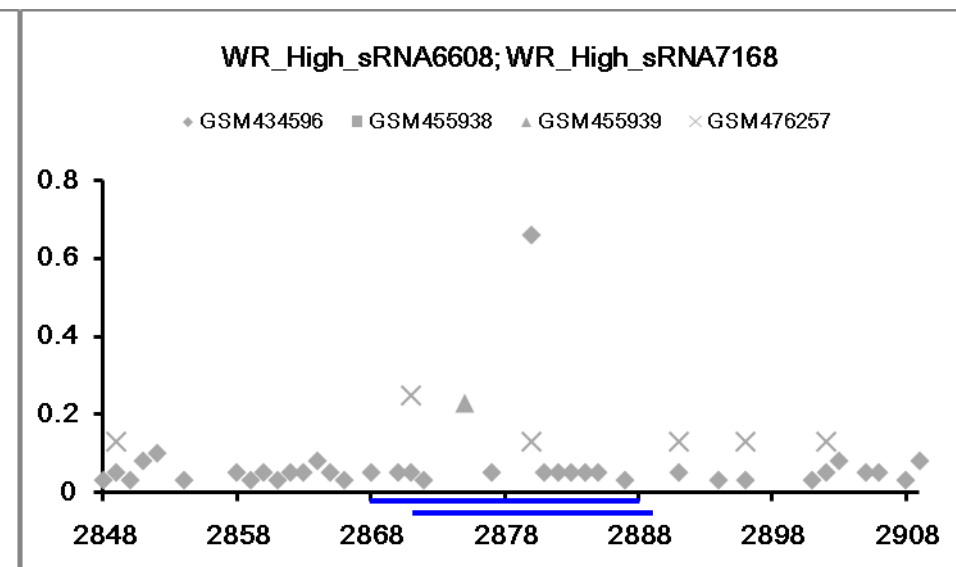

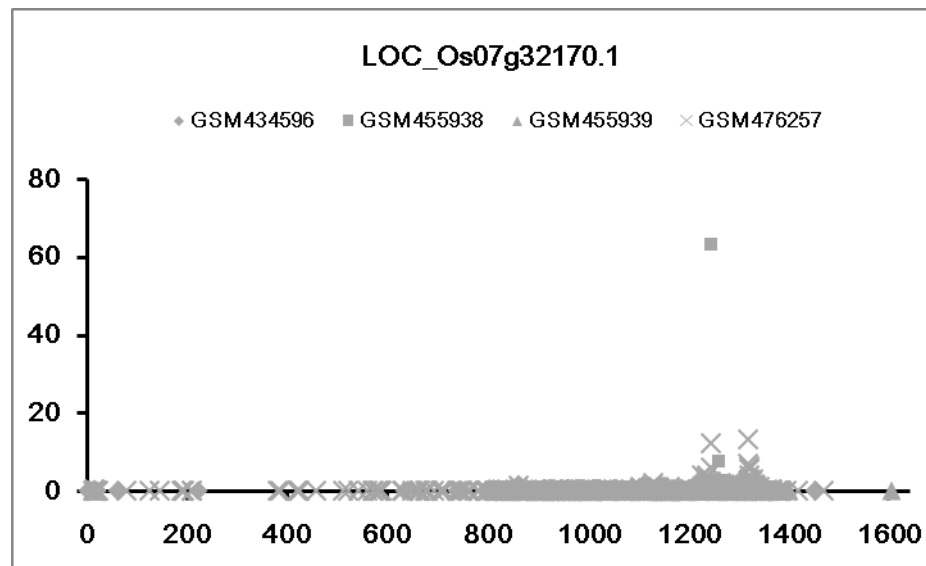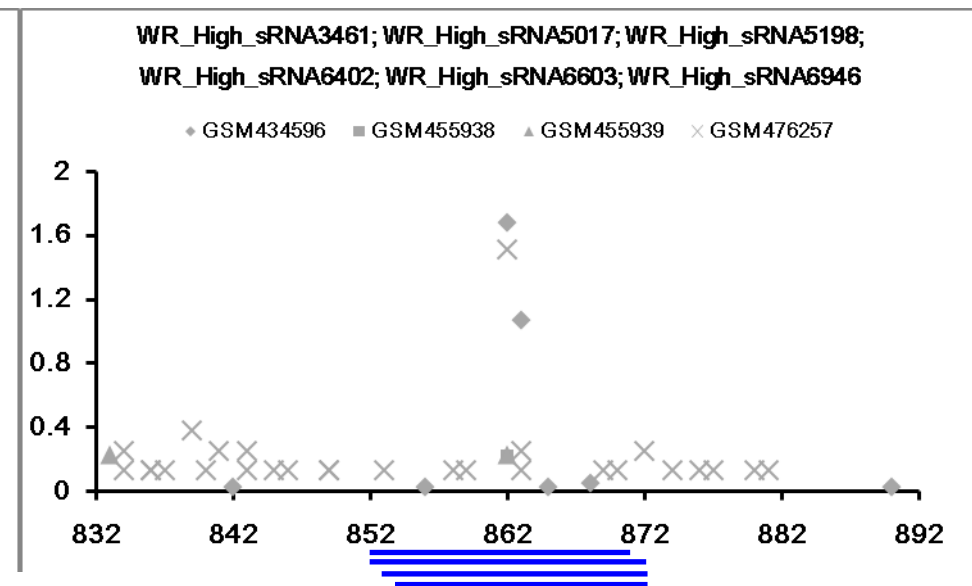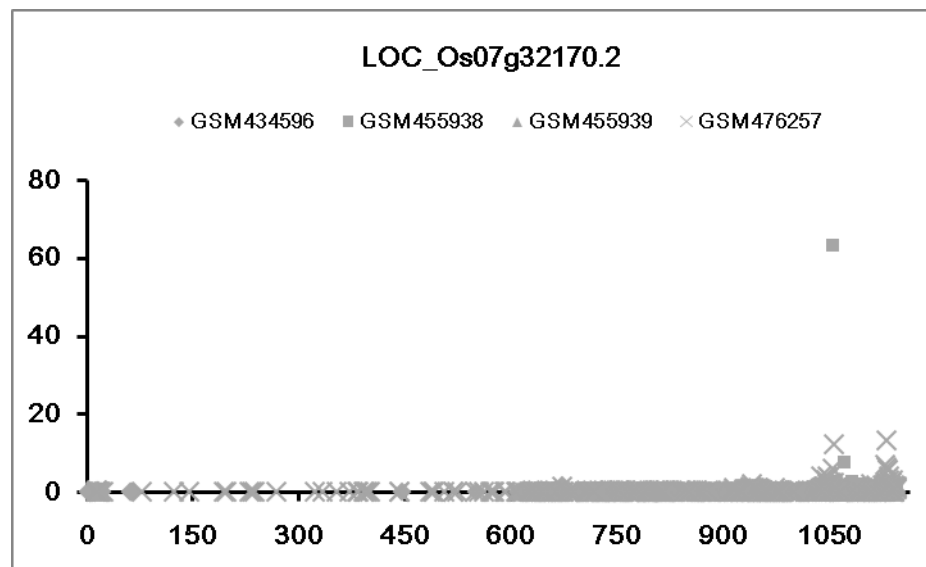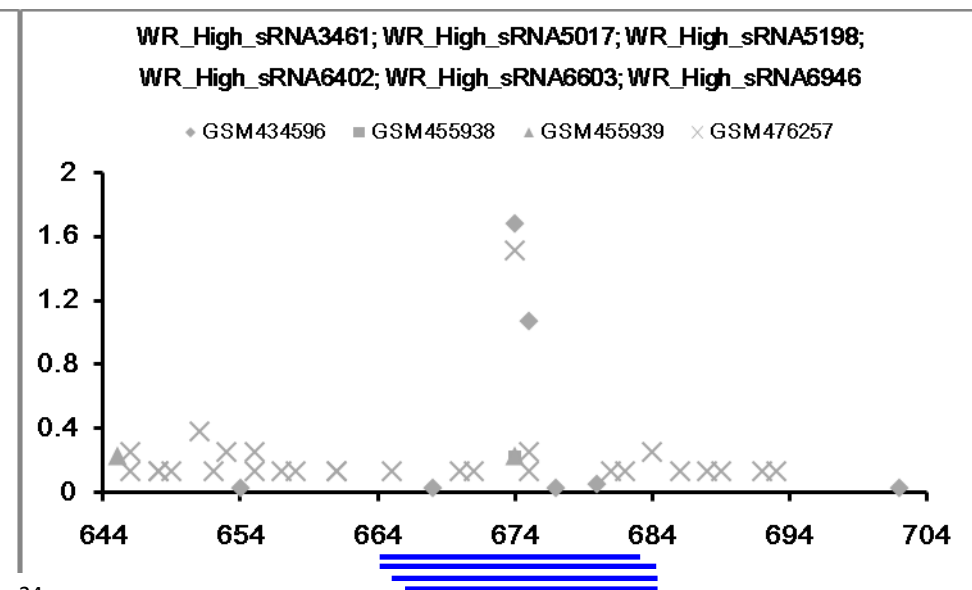

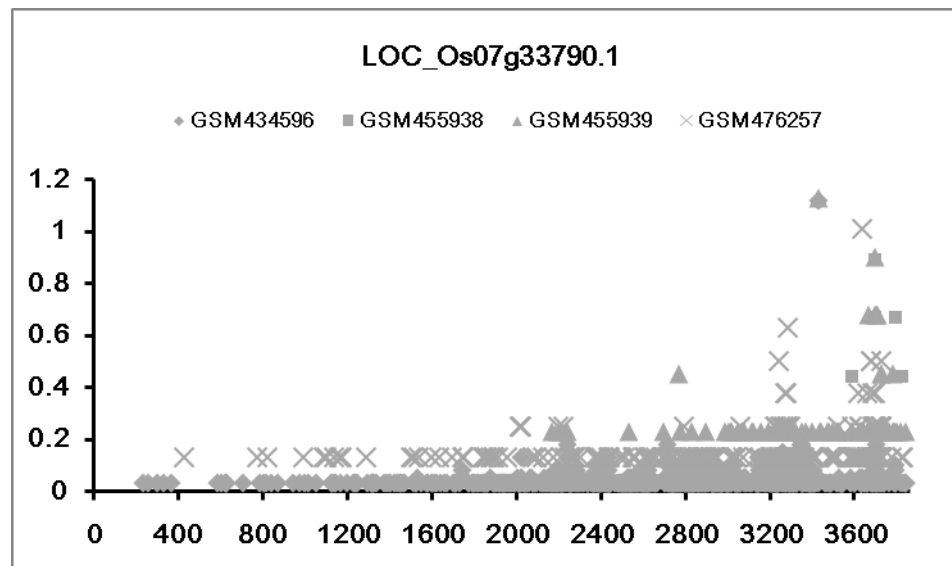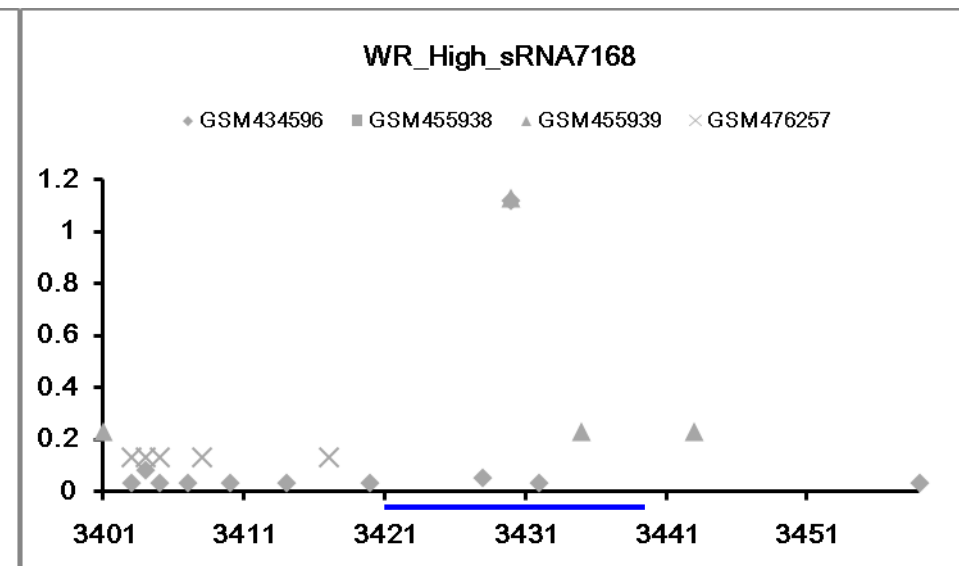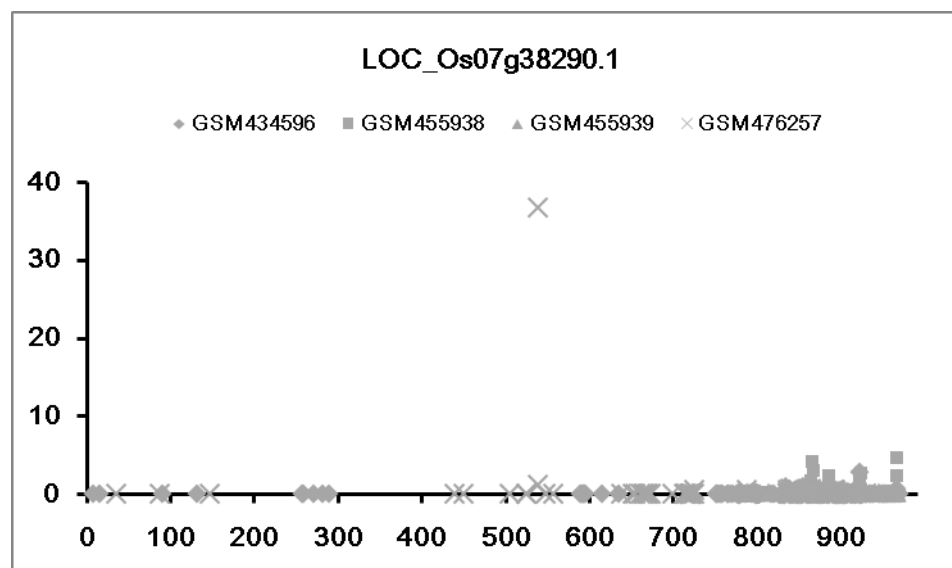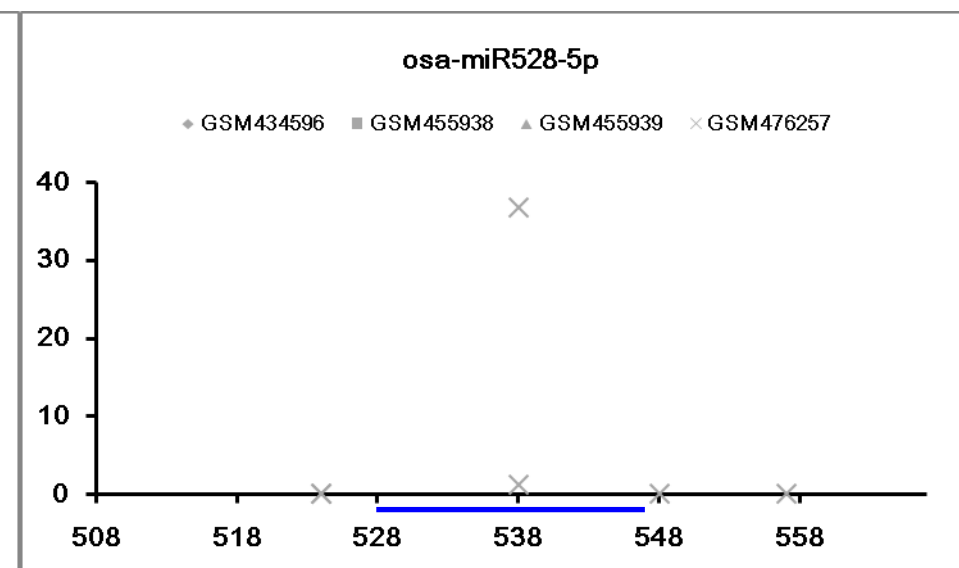

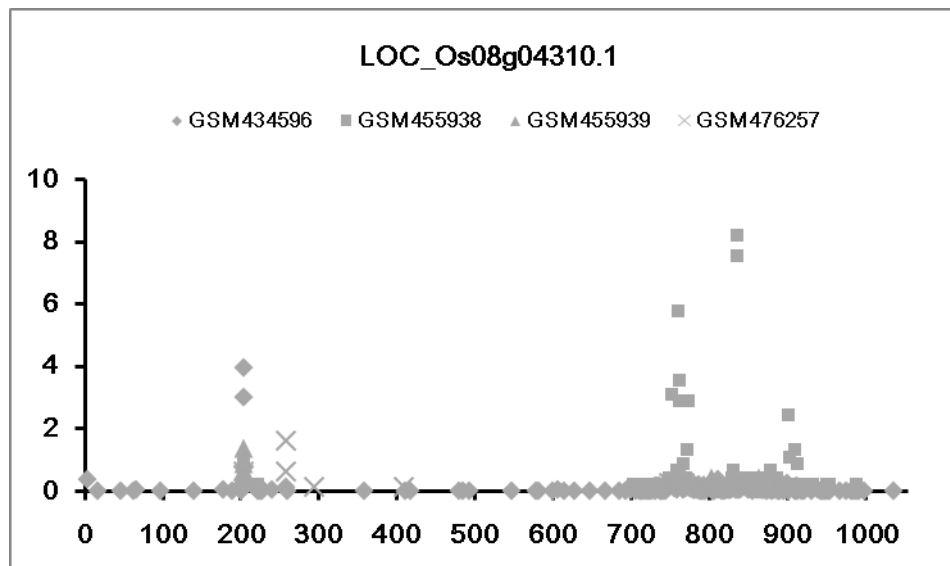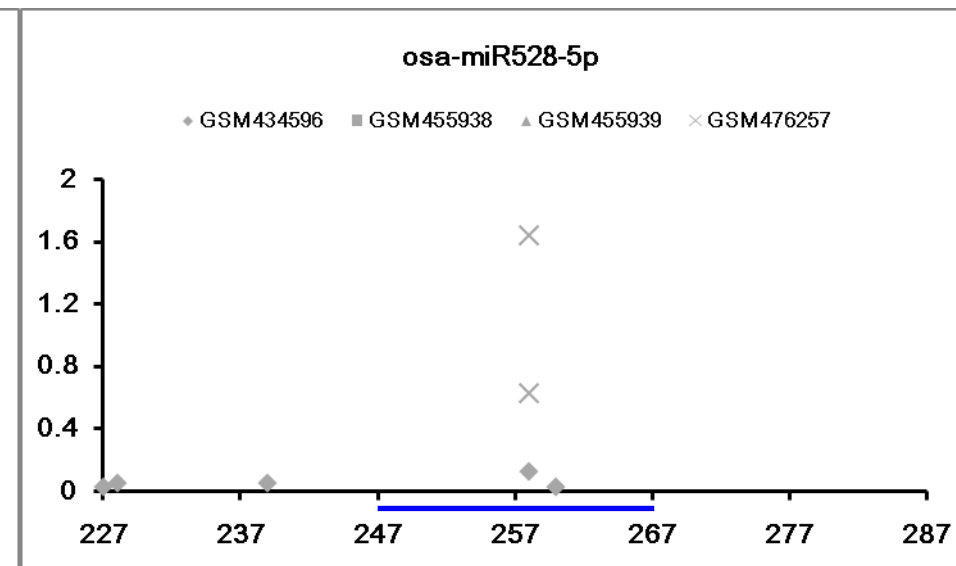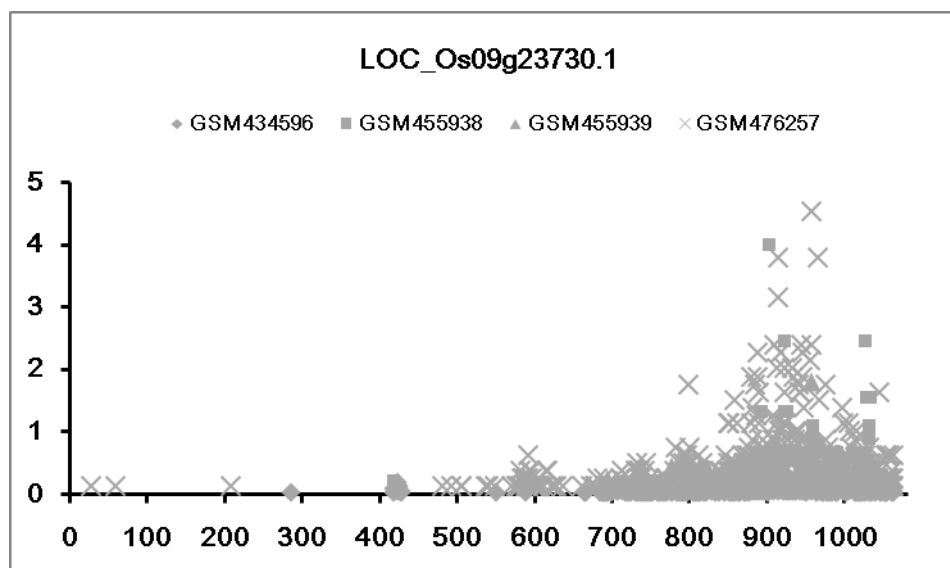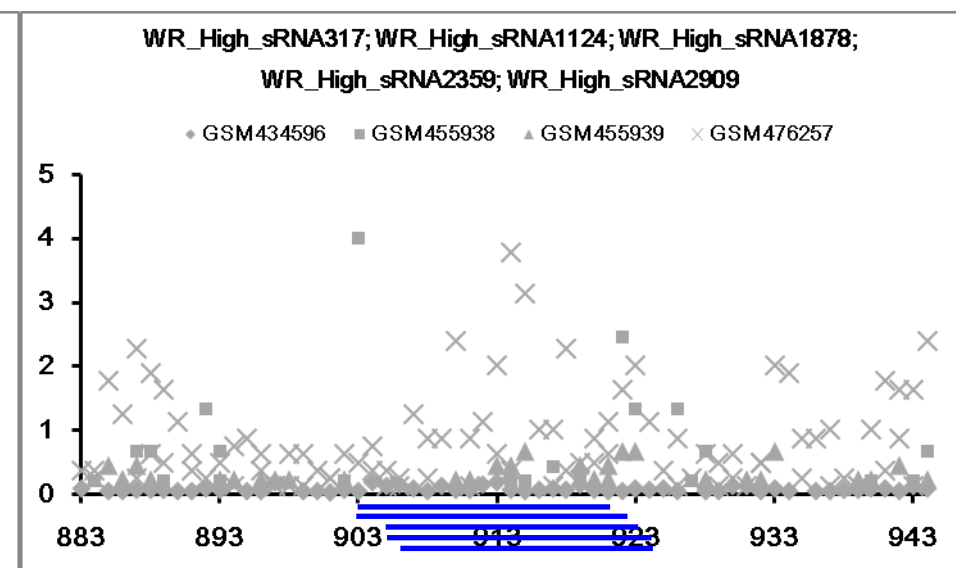

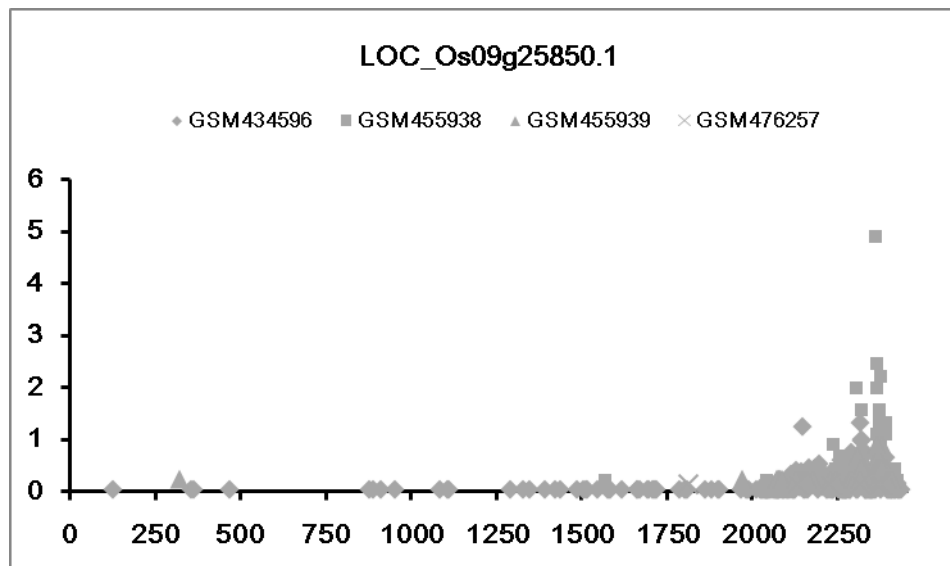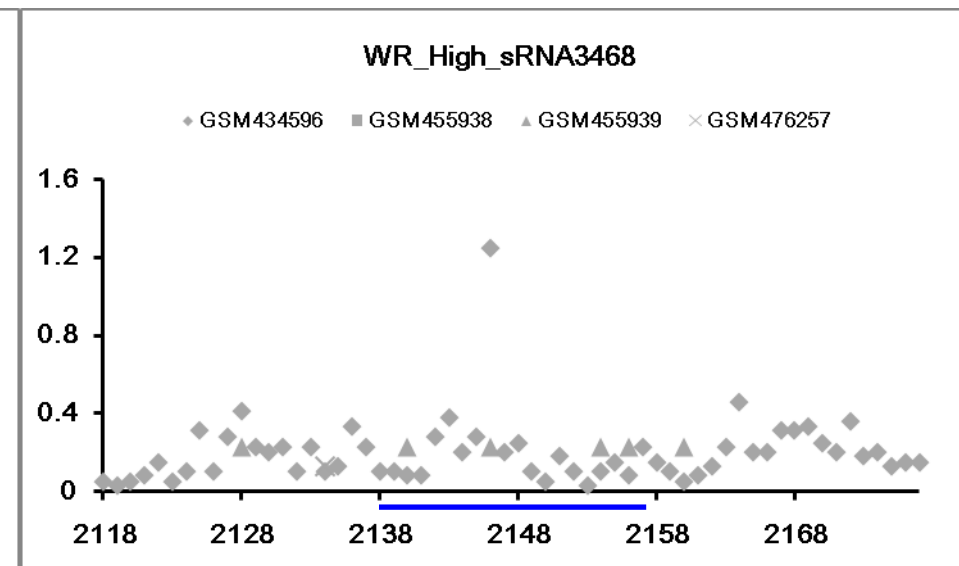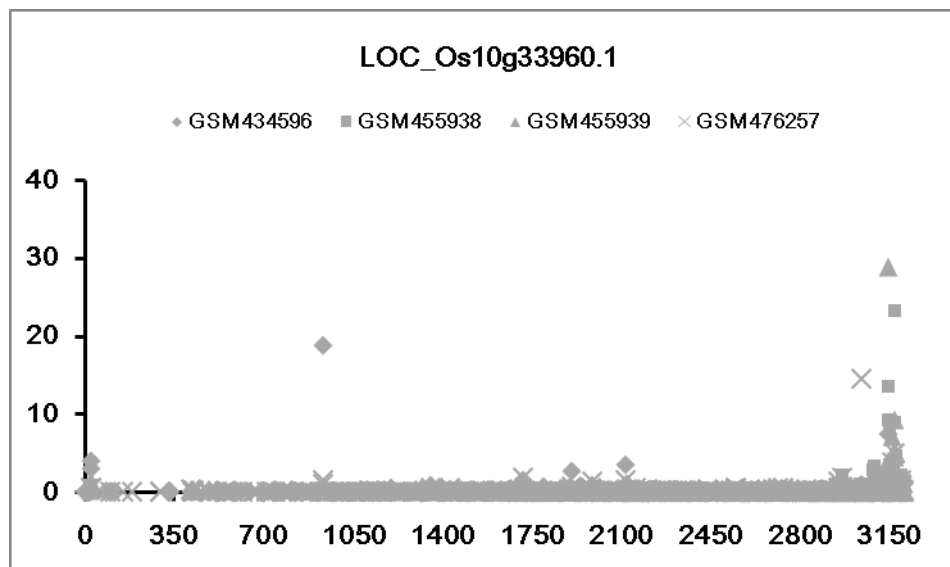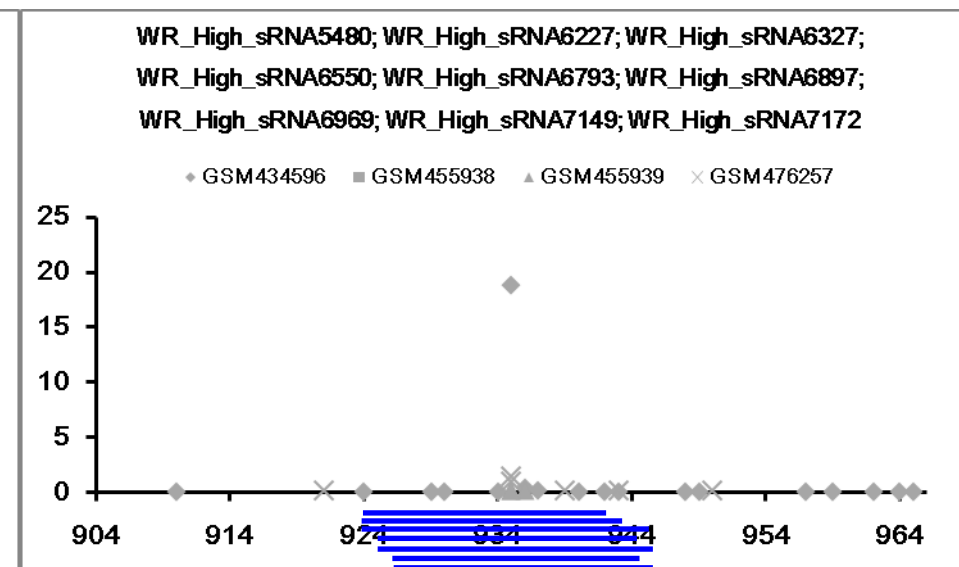

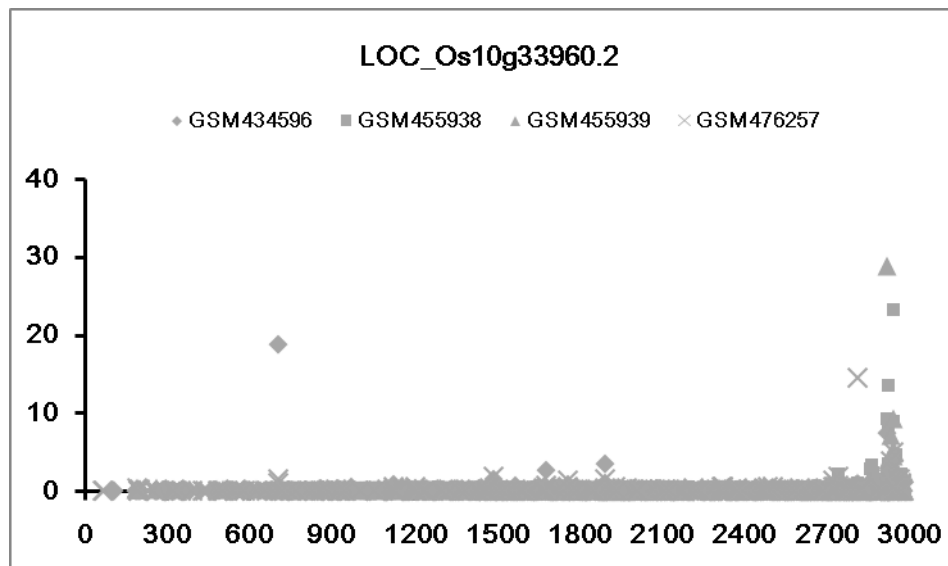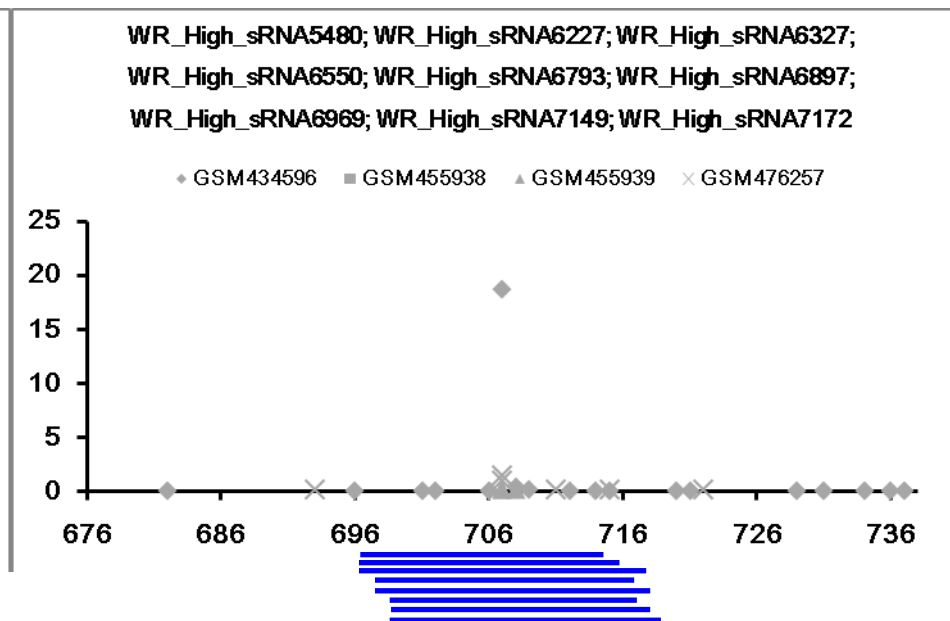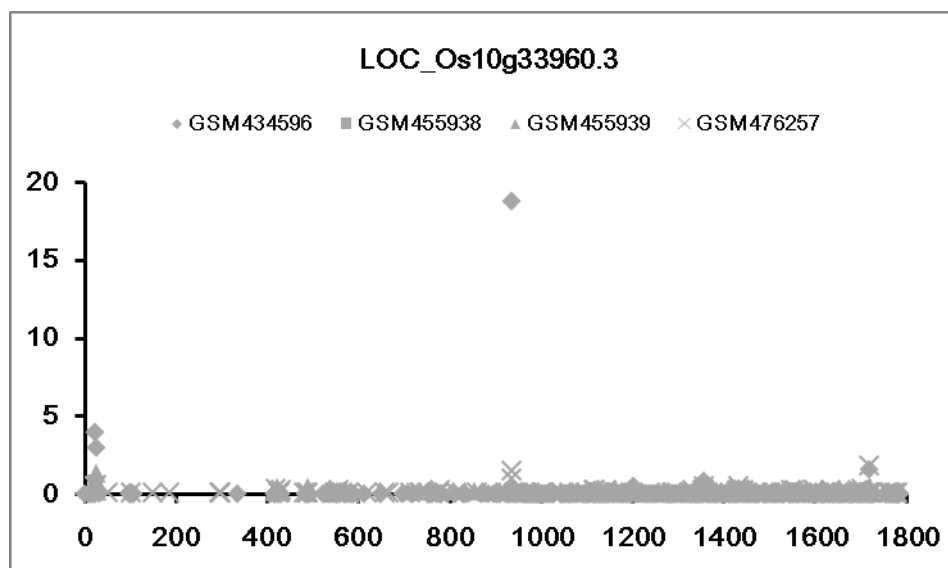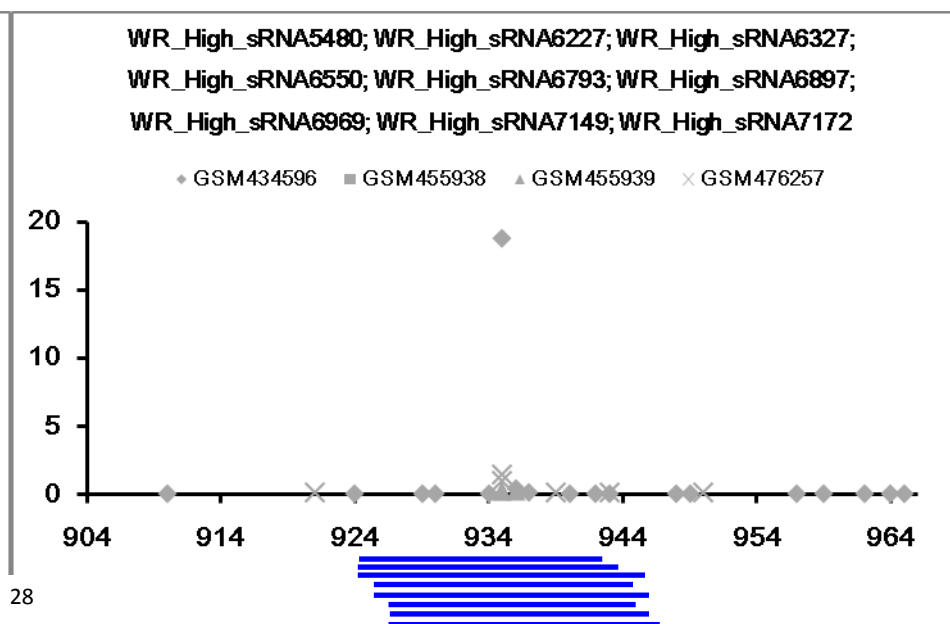

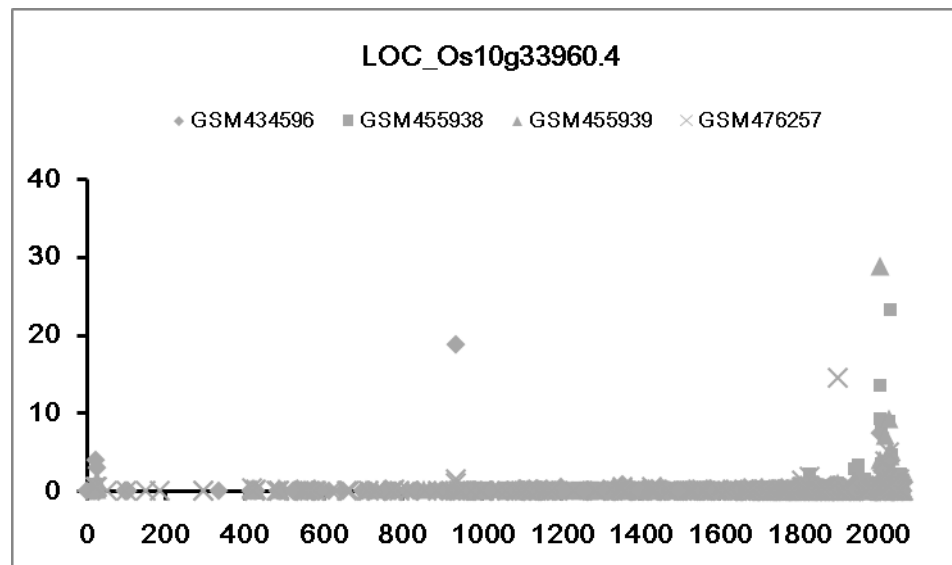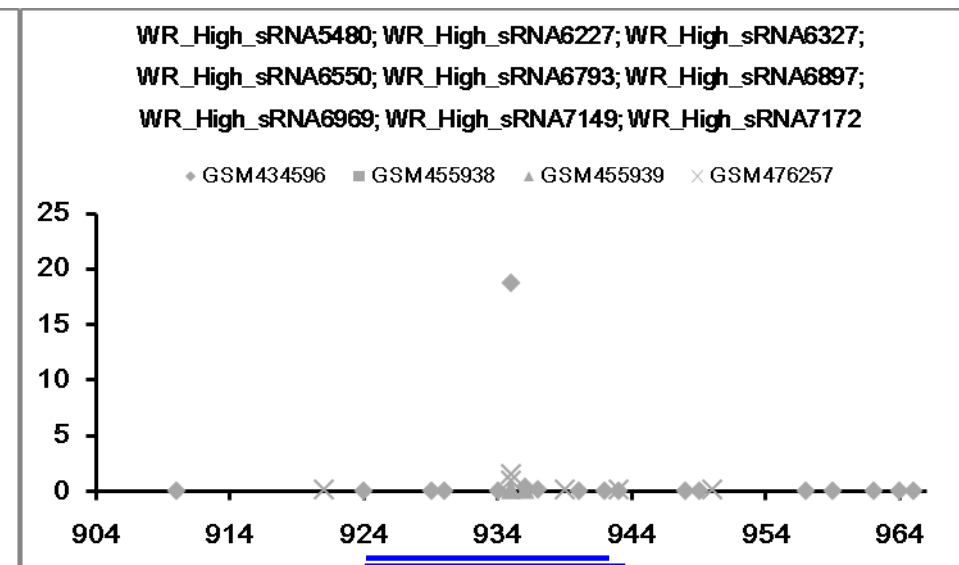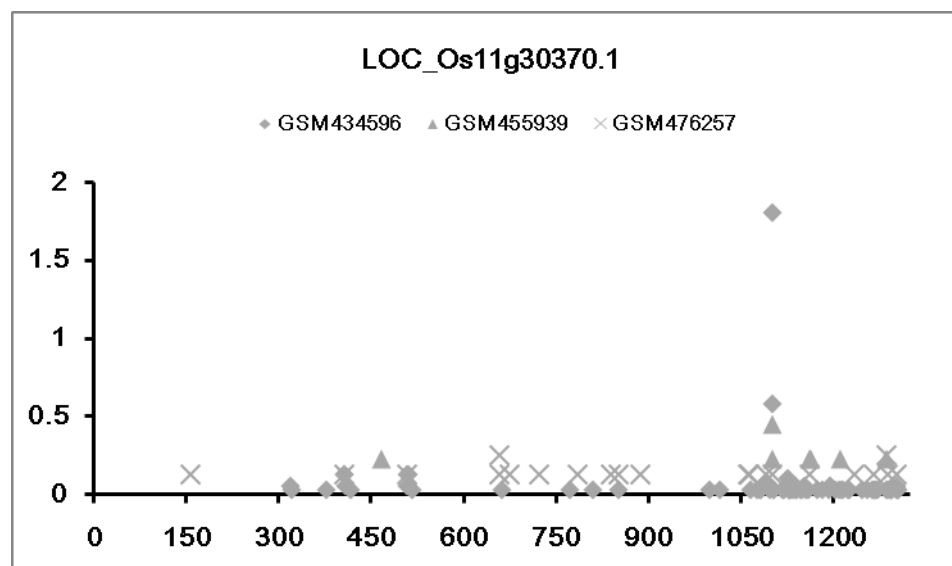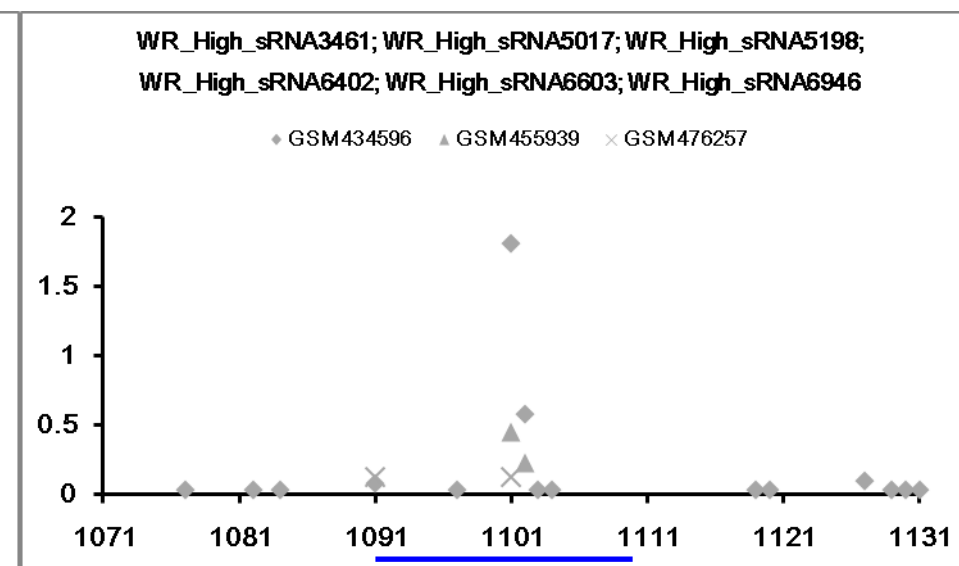

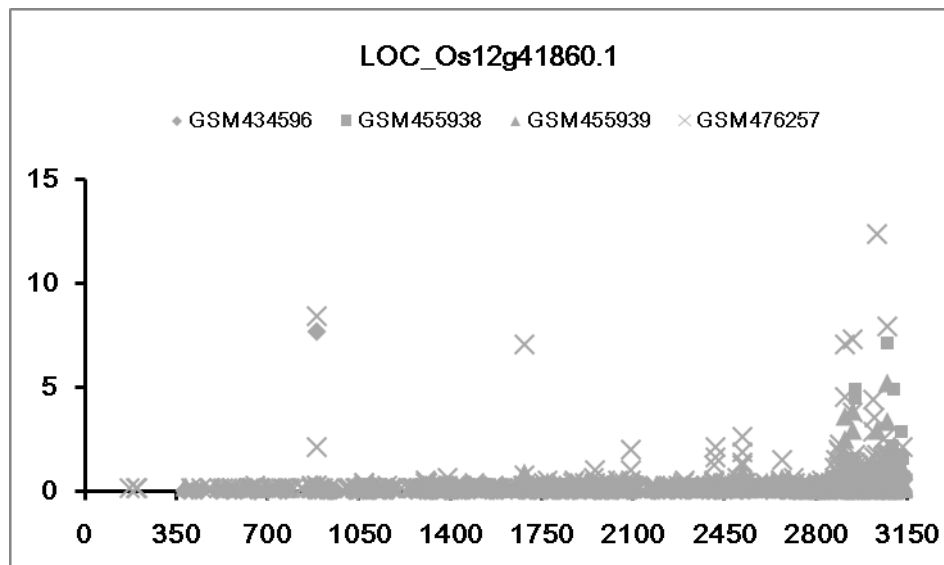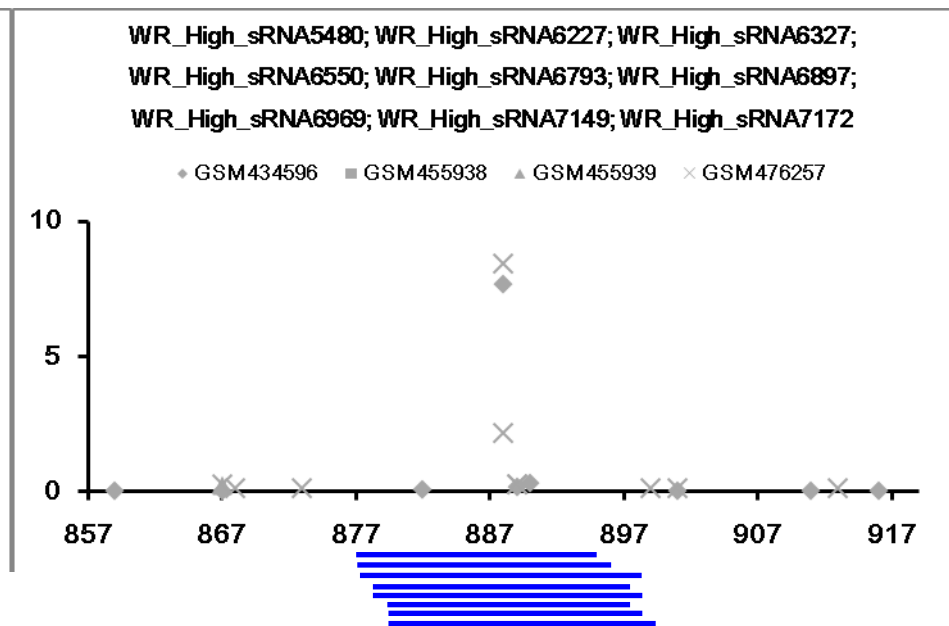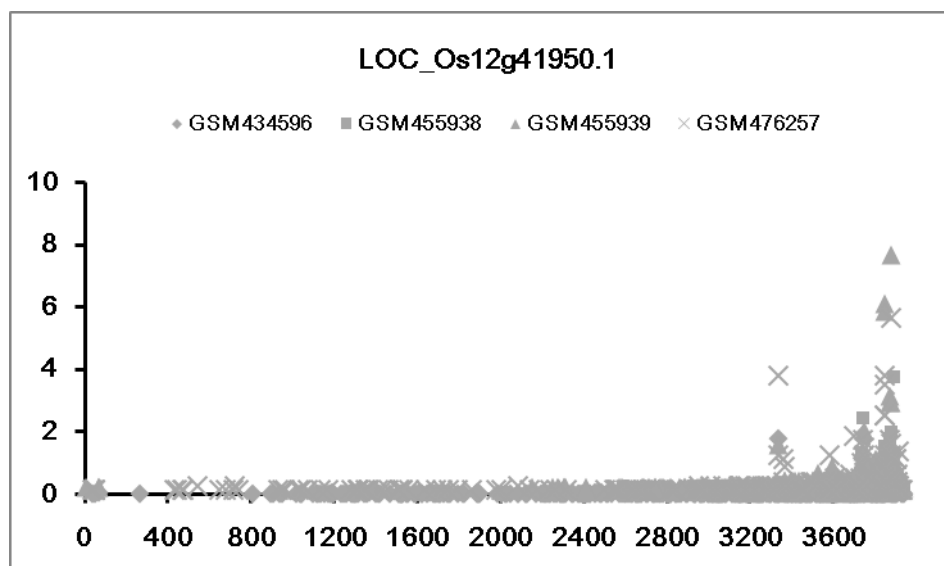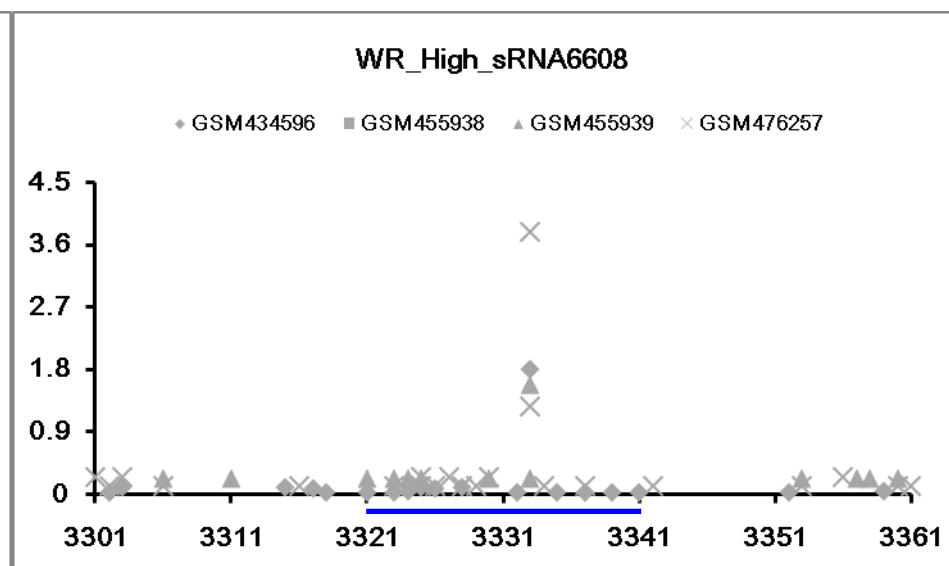

Supplement: Additional file 6: Figure S2 — Degradome sequencing data-based validation of the targets of the AGO1-enriched sRNAs highly expressed in the whole roots of rice. [file 1471-2164-14-510-S6.pdf]

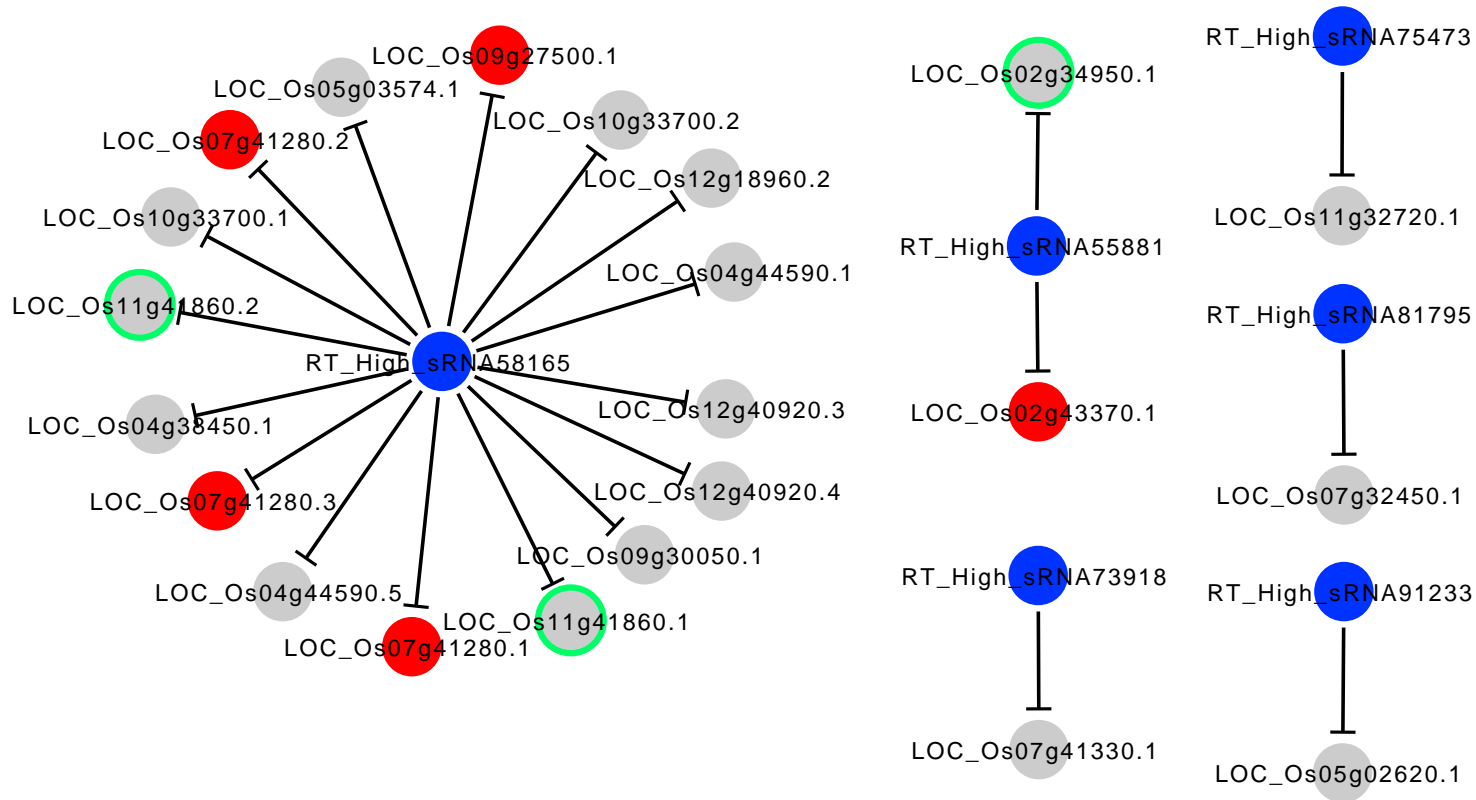

Supplement: Additional file 7: Figure S3 — Network mediated by the AGO1-enriched sRNAs highly expressed in the root tips of rice. [file 1471-2164-14-510-S7.pdf]

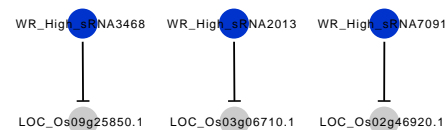

Supplement: Additional file 8: Figure S4 — Network mediated by the AGO1-enriched sRNAs highly expressed in the whole roots of rice. [file 1471-2164-14-510-S8.pdf]
